# Mapping pre-harvest sprouting resistance loci in AAC Innova × AAC Tenacious spring wheat population

**DOI:** 10.1186/s12864-021-08209-6

**Published:** 2021-12-15

**Authors:** Raman Dhariwal, Colin W. Hiebert, Mark E. Sorrells, Dean Spaner, Robert J. Graf, Jaswinder Singh, Harpinder S. Randhawa

**Affiliations:** 1 Lethbridge Research and Development Centre, Agriculture and Agri-Food Canada, 5403 1st Avenue South, Lethbridge, AB T1J 4B1 Canada; 2 Morden Research and Development Centre, Agriculture and Agri-Food Canada, 101 Route 100, Morden, MB R6M 1Y5 Canada; 3 School of Integrative Plant Science, Plant Breeding and Genetics Section, Cornell University, 240 Emerson Hall, Ithaca, NY 14853 USA; 4 Department of Agricultural, Food & Nutritional Science, University of Alberta, Edmonton, AB T6G 2P5 Canada; 5 Department of Plant Science, McGill University, Ste-Anne-de-Bellevue, QC H9X 3V9 Canada

**Keywords:** Seed dormancy, Seed coat color, Phytohormones, Genetic and epigenetic factors, Epistasis interactions

## Abstract

**Background:**

Pre-harvest sprouting (PHS) is a major problem for wheat production due to its direct detrimental effects on wheat yield, end-use quality and seed viability. Annually, PHS is estimated to cause > 1.0 billion USD in losses worldwide. Therefore, identifying PHS resistance quantitative trait loci (QTLs) is crucial to aid molecular breeding efforts to minimize losses. Thus, a doubled haploid mapping population derived from a cross between white-grained PHS susceptible cv AAC Innova and red-grained resistant cv AAC Tenacious was screened for PHS resistance in four environments and utilized for QTL mapping.

**Results:**

Twenty-one PHS resistance QTLs, including seven major loci (on chromosomes 1A, 2B, 3A, 3B, 3D, and 7D), each explaining ≥10% phenotypic variation for PHS resistance, were identified. In every environment, at least one major QTL was identified. PHS resistance at most of these loci was contributed by AAC Tenacious except at two loci on chromosomes 3D and 7D where it was contributed by AAC Innova. Thirteen of the total twenty-one identified loci were located to chromosome positions where at least one QTL have been previously identified in other wheat genotype(s). The remaining eight QTLs are new which have been identified for the first time in this study. Pedigree analysis traced several known donors of PHS resistance in AAC Tenacious genealogy. Comparative analyses of the genetic intervals of identified QTLs with that of already identified and cloned PHS resistance gene intervals using IWGSC RefSeq v2.0 identified *MFT-A1b* (in QTL interval *QPhs.lrdc-3A.1*) and *AGO802A* (in QTL interval *QPhs.lrdc-3A.2*) on chromosome 3A, *MFT-3B-1* (in QTL interval *QPhs.lrdc-3B.1*) on chromosome 3B, and *AGO802D*, *HUB1*, *TaVp1-D1* (in QTL interval *QPhs.lrdc-3D.1*) and *TaMyb10-D1* (in QTL interval *QPhs.lrdc-3D.2*) on chromosome 3D. These candidate genes are involved in embryo- and seed coat-imposed dormancy as well as in epigenetic control of dormancy.

**Conclusions:**

Our results revealed the complex PHS resistance genetics of AAC Tenacious and AAC Innova. AAC Tenacious possesses a great reservoir of important PHS resistance QTLs/genes supposed to be derived from different resources. The tracing of pedigrees of AAC Tenacious and other sources complements the validation of QTL analysis results. Finally, comparing our results with previous PHS studies in wheat, we have confirmed the position of several major PHS resistance QTLs and candidate genes.

**Supplementary Information:**

The online version contains supplementary material available at 10.1186/s12864-021-08209-6.

## Background

Pre-harvest sprouting (PHS) or germination of mature grains on wheat heads before harvest, often caused by cool, wet conditions during the harvest season, may result in significant losses in wheat yield, end-use quality (test weight, milling and baking properties), seed viability and seedling vigor [1–6]. PHS is a global problem which occurs in many countries of the world including Australia, Canada, China, Germany, India, Japan and USA [7, 8]. In Canada, PHS causes significant damage to wheat production in the eastern and northern Prairies. PHS is estimated to cost the wheat industry average losses of US$100 million in Canada and > $1 billion worldwide annually in the years favorable for PHS [9–12].

Continuous wet conditions at ripening triggers a sequence of physiological processes in the seed, which includes the release of hydrolytic enzymes such as α-amylases, lipases, and proteases [1, 13]. Reduced grain test-weight and low falling number are observed in PHS affected samples due to the conversion of starch to glucose by α-amylases [14, 15]. Increased activity of amylases, lipases and proteases affect bread and noodle making quality [1, 15, 16]. Losses in functional baking quality due to PHS may include low flour absorption, reduced dough strength and loaf volume, and poor crumb structure [17, 18]. Additionally, PHS can affect baking properties by making the dough porous, sticky and off-color [1].

PHS is influenced by several environmental and genetic factors [2, 4, 6] and is associated with several developmental, physiological, and morphological features of the seed and spike (reviewed in [1]). These includes seed coat (pericarp) color and permeability, α-amylase activity, level of plant growth hormones (abscisic acid, ABA; gibberellin, GA; auxin), and seed dormancy (reviewed in [1]). The presence of awns, spike shape, openness of florets, glume rigidity and germination inhibitors in the husk and bracts [13, 19, 20], along with glume epicuticular wax, glume adherence and head inclination, etc. [21] also affect PHS resistance [6]. Among all these characteristics, seed dormancy [1, 5–7] and spike morphology [6] are the most important genetic factors influencing PHS resistance [6].

Seed dormancy is believed to be the predominant control of PHS resistance [7] and has received much attention in breeding programs [1]. Seed dormancy prevents germination at early stages after physiological maturity and it dissipates over time so that germination occurs in more favorable conditions to enable the survival of plants in hostile environments [7]. Seed dormancy is primarily seed coat- and embryo-imposed [6, 22].

The seed coat provides dormancy by acting as a physical barrier to imbibition and radicle growth [7] but additionally may stop germination by seed coat inhibitors [6, 23, 24]. Seed coat imposed dormancy mechanisms correlate positively with seed coat color due to phenolic compounds in diverse species [1]. The red grain color due to catechin and proanthocyanidin flavanol pigments [25, 26] in the testa (seed coat) of wheat is also associated with seed dormancy [1, 22, 27]. *R* genes genetically control testa color in wheat and are mapped to the distal region of homeologous group 3 chromosomes [28]. *R* genes act as transcriptional activators of the flavonoid synthesis pathway genes chalcone synthase (*CHS*), chalcone isomerase (*CHI*), flavanone 3-hydroxylase (*F3H*), and dihydroflavonol 4-reductase (*DFR*) [29]. Myb-type transcription factor genes (*Tamyb10-A1*, *Tamyb10-B1* and *Tamyb10-D1*), which are located to the same genetic intervals as the *R* loci, control the red grain color of wheat by up-regulating the flavonoid biosynthesis pathway structural genes *DFR*, *CHI*, *F3H*, and *CHS* [1, 29].

Embryo-imposed dormancy is precisely regulated by seed developmental processes [7]. ABA and its crosstalk with GA and auxin play fundamental roles in regulating embryo-imposed dormancy [1, 7]. A number of genes involved in ABA biosynthesis and signal transduction have been identified to have roles in seed dormancy in diverse species [30]. The *Viviparous-1* (*Vp-1*)/*Abscisic Acid Insensitive3* (*ABI3*) gene, which encodes a dormancy related-transcription factor and is involved in ABA signal transduction, is an important regulator of late embryogenesis in maize and late embryo development in wheat [31–33]. The *TaVp-1* loci are located approximately 30 cM proximal to the *R* genes on the group 3 chromosomes of wheat [29, 34, 35]. A number of other ABA synthesis and signal transduction pathway genes such as wheat homolog of *Mother of FT and TFL1* (*TaMFT-like*/*TaPHS1*), *ABA-induced Wheat Plasma Membrane 19* (*PM19-A1/A2*) [36], wheat homolog of *cytochrome P450 family 707 subfamily A polypeptide 1* gene (*TaCYP707A1*) and *Delay of Germination 1* (*TaDOG1*) have been found associated with seed dormancy [2, 37–42].

Several studies demonstrated that epigenetic modifications through DNA [43] and histone methylation [44, 45] can also influence seed dormancy and PHS resistance [5]. Histone deacetylases have also been found to modulate seed germination and ABA-induced gene expression in *Arabidopsis* [46, 47] and have been found to be modulated by ABA in barley [48]. Recently, the role of *ARGONAUTE* genes of ARG4_9 class, which play key roles in DNA silencing in plants through the RNA dependant DNA methylation (RdDM) pathway, was explored in wheat and barley [5, 43]. An association of DNA methylation and polymorphism in *ARGONAUTE* gene *AGO802B* on chromosome 3B and PHS resistance was demonstrated in embryos of PHS resistant and susceptible cultivars of wheat [5].

All wheat chromosomes possess quantitative trait loci (QTLs) associated with PHS resistance, resulting in a total 110 loci in wheat [6]. QTLs have been repeatedly reported on groups 3 and 4 chromosomes from different wheat genotypes [6], such as the major QTLs *QPhs.pseru-3A/TaPHS1* on chromosome arm 3AS [42, 49, 50] and *Phs1* on chromosome arm 4AL [51, 52]. In addition to genes/QTLs mentioned above, causal/candidate genes from some of the PHS associated QTLs have also been cloned/identified such as *mitogen-activated protein kinase kinase 3* (*TaMKK3-A*) for *Phs1* QTL on chromosome arm 4AL [52], *TaSdr-A1a* [53], and *TaSdr-B1* [7].

In wheat, red-grained cultivars are generally more PHS resistant than those that are white-grained [34]. Using genealogical analysis of 148 red-grained and 63 white-grained North-American spring wheat cultivars with varying level of PHS resistance, Martynov and Dobrotvorskaya [54] found that cultivars with different genetic backgrounds may have different sources of resistance. The genetic resistance in red-grained cultivars came from source genotype groups (i) Crimean, Hard Red Calcutta and Iumillo, (ii) Button, Kenya 9 M-1A-3 and Kenya-U, and (iii) Red Egyptian and Kenya BF4-3B-10 V1, respectively, via donor cultivars (i) Thatcher, (ii) Kenya-Farmer, and (iii) Kenya-58 [54]. The genetic resistance in white-grained cultivars came from genotypes Akakomugi, Crimean, Hard Red Calcutta, Hybrid English, Iumillo, Ostka Galicyjska, Rough Chaff, White Red King and Turco via donor cultivars Frontana, RL2265 and Thatcher [54]. RL4137 is another important PHS-resistant line of Canadian origin and has been included in the parentage of vast majority of PHS resistant North-American red- and white-grained spring wheat accessions [54, 55]. In addition to the above mentioned North American sources, a number of other red- and white-grained genotypes have been reported to possess PHS resistance across the globe. Some of these include Chinese landraces RSP and Chinese Spring [56, 57], French cv Renan [34], Indian breeding line SPR8198 and cv HD2329 [58], Japanese breeding line OS21–5 and cv Zenkoujikomugi [2, 51, 57, 59, 60], and Mexican cv Opata [61].

Domestication and the desire of breeders to develop cultivars using reduced time frames as required in contra-season nurseries and speed breeding, continued selection for uniform and rapid germination and seedling establishment in wheat cultivars has worked against seed dormancy and made modern cultivars susceptible to PHS [38, 62–67]. Therefore, breeding programs have to meet contradictory demands of high level of seed dormancy during harvest time and high level of germination after seeding [2]. To satisfy these demands, different mechanisms controlling PHS resistance and subsequent germination after seeding must be identified [2].

AAC Tenacious is a modern Canadian red-grained, highly PHS resistant spring wheat cultivar [68] which has several North American PHS-resistant sources, such as RL4137, in its parentage. However, the PHS resistance of AAC Tenacious is not yet completely understood. The objectives of the present study were to identify QTLs for PHS resistance in AAC Tenacious using a large doubled haploid (DH) population, compare identified QTLs with the previously reported QTLs, and identify candidate genes using comparative analyses.

## Results

### PHS resistance evaluation

**Fig. 1:**
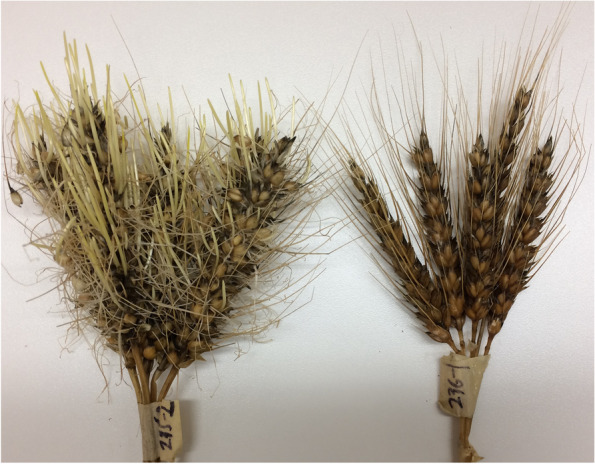
Pre-harvest sprouting (PHS) phenotypes of population parents after 4 days in a mist chamber. PHS-susceptible cultivar AAC Innova is shown on left-hand side while PHS-resistant cultivar AAC Tenacious is shown on right-hand side

**Fig. 2:**
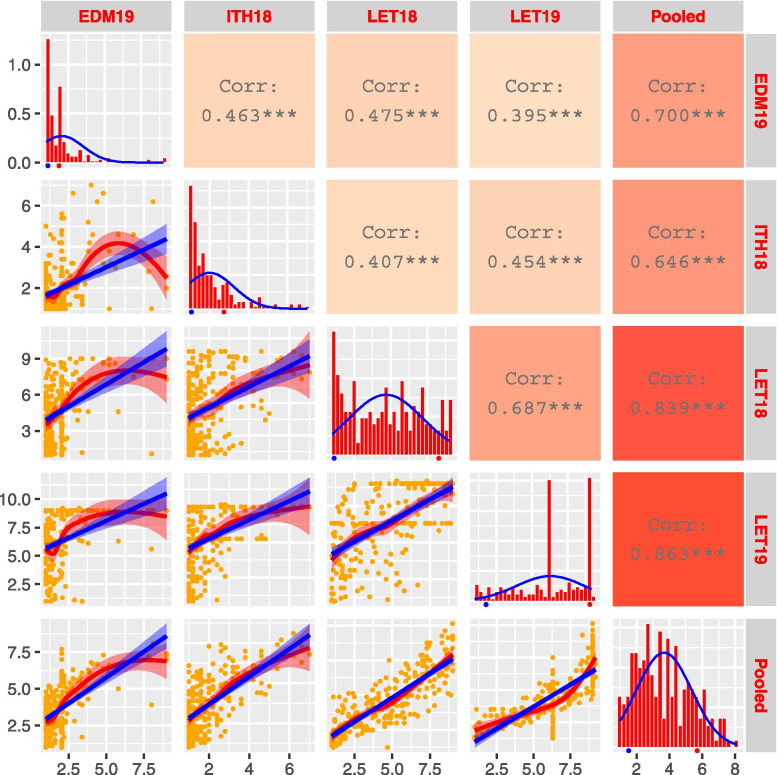
Frequency distribution and correlation scatterplots for pre-harvest sprouting (PHS) score of doubled haploid (DH) lines. Frequency distribution histograms with normal distribution curve (blue line) for PHS of DH lines grown at Edmonton 2019 (EDM19), Ithaca 2018 (ITH18), Lethbridge 2018 (LET18) and Lethbridge 2019 (LET19) as well as pooled data are shown on main diagonal. The means of the parental genotypes AAC Tenacious and AAC Innova are indicated by blue and red dots, respectively, beneath frequency distribution plots. Scatterplots with regression lines, linear (blue) and exponential (red), for each environment pair are shown on the left side of the main diagonal. Orange dots on scatterplots represent PHS score of DH lines. Correlation coefficients (*r*) between each pair of environments, and each environment and the pooled data are displayed on the right side of the main diagonal. Color intensity (light red to dark red) on *r* boxes indicate the depth of association between environments under evaluation

Strong phenotypic variability for sprouting was observed between the parents (Fig. 1), check cultivars (Additional file 1: Fig. S1) and DH lines across environments, except Edmonton 2019 (Figs. 1 and 2, and Additional file 2: Table S1) but the differences were significant in all the environments. In Edmonton 2019, weather conditions at harvest were relatively cold, which delayed physiological maturity. ANOVA also showed significant environment and genotype effects for PHS (Additional file 2: Table S2). The estimated broad-sense heritability of the PHS trait was 0.71.

Parent cultivars showed different PHS phenotypes across environments. The average PHS score for the resistant parent, AAC Tenacious, ranged from 1.0 in Edmonton 2019 and Ithaca 2018 environments to 1.4 in Lethbridge 2019. The average score for the PHS susceptible parent, AAC Innova, ranged from 1.8 in Edmonton 2019 to 8.7 in Lethbridge 2019 (Additional file 2: Table S1). Mean PHS data showed that AAC Tenacious and AAC Penhold were the most resistant cultivars while AAC Brandon, AAC Awesome and AC Andrew were the most susceptible cvs among the parent and check cultivars (Additional file 2: Table S1).

The DH population also differed broadly for PHS, with the resistant and susceptible DHs deviating by the rating score of 7.5 where the mean of population was 3.7 (Additional file 2: Table S1). Population PHS means were within the range of the two parents across environments (Additional file 2: Table S1). However, among the parents, the lowest PHS was seen in Edmonton 2019 (mean 1.4, ranged from 1.0 to 1.8) and the highest PHS was seen in Lethbridge 2019 (mean 5.05, ranged from 1.4 to 8.7) (Additional file 2: Table S1). Moreover, Edmonton 2019 and Ithaca 2018 environments were phenotypically similar, as were Lethbridge 2018 and 2019 (Additional file 2: Table S1). Conversely, Lethbridge 2019 had the highest PHS mean scores while Edmonton 2019, Ithaca 2018 and Lethbridge 2018 had the first, second and third lowest means, respectively (Additional file 2: Table S1).

**Fig. 3:**
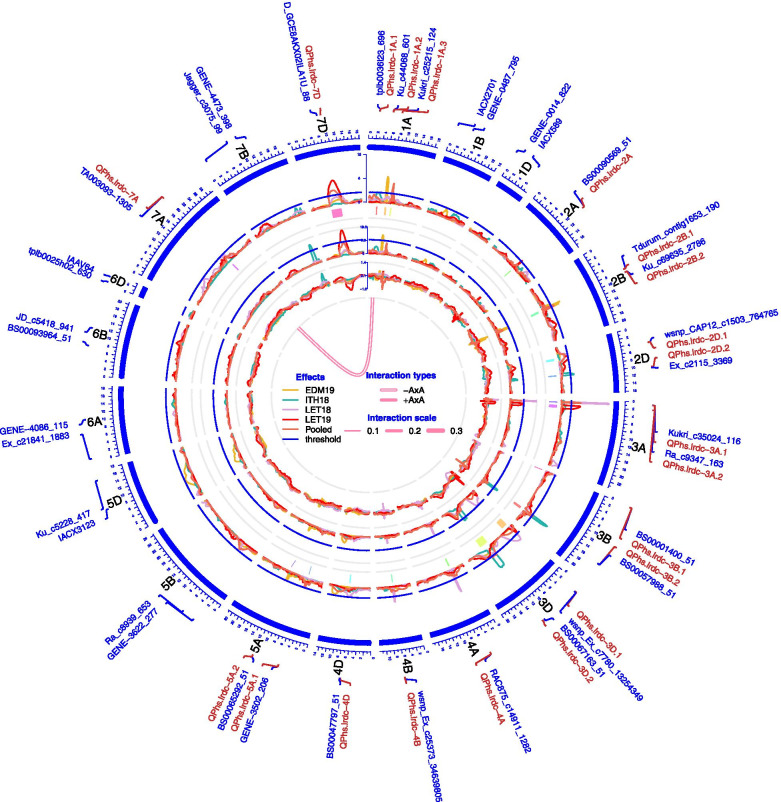
Circos diagram. Complete results of quantitative trait loci (QTL) and epistasis interaction identified for pre-harvest sprouting (PHS) resistance from AAC Innova/AAC Tenacious doubled haploid mapping population using composite interval mapping are shown in Circos diagram. The outermost track shows the 21 chromosomes (1A -7D) arranged in clockwise direction with PHS resistance QTLs (in red color font) and their linked or few randomly selected markers (in blue color font) in 0.1X scale (cM). Three inner tracks and line connections in the middle, respectively, represent the mean LOD score (second track from outside), % phenotypic variation (*R*^*2*^) explained (third track from outside), additive effect (AE) (fourth track from outside) of individual QTLs and epistatic effect (AA) of digenic QTL × QTL interactions (line connection in the middle) for PHS resistance in different environments. In the second and third tracks from outside, blue lines show a LOD threshold of 2.5 and *R*^*2*^ threshold of 10%, respectively. QTL confidence intervals are shown in different colors beneath the QTL scans in second track from outside. LOD score, % *R*^*2*^ and AE peaks for different environments are represented by different colors as shown in the effects legend in the middle of the Circos diagram. A negative QTL × QTL interaction between QTLs *QPhs.lrdc-1A.1* and *QPhs.lrdc-7A*, on chromosomes 1A and 7A, is shown as line connection in the middle of the Circos diagram. The width of the line connection represent the strength of AA effect, as shown in the AA interaction scale

Frequency distribution plots showed a skewed distribution (towards resistance) of sprouting phenotypes in the population across environments except in Lethbridge 2019 (Fig. 3). However, a broader range of genotypes was observed across environments. In Lethbridge 2019, a number of DHs which previously showed less sprouting, revealed relatively high sprouting, perhaps due to some epigenetic changes.

Correlation coefficients (*r*) for the PHS scores between any pair of environments were moderate to high (ranged from 0.40–0.69) with a moderate (0.48) mean correlation coefficient (Fig. 2). No strong trend was observed in correlations between locations within a single year or 2 years, though the highest correlation (0.69) was observed at the Lethbridge location between 2018 and 2019 (Fig. 2). Heritability across the four trial environments was 0.71. Since the correlation between environments was generally lower than broad-sense heritability, these results suggest the existence of a strong environmental influence on genotypes [69].

### Quantitative trait loci for PHS resistance

#### Main-effect QTLs

Composite interval mapping (CIM) analysis was carried out individually for each environment using PHS data of individual environments as well as the pooled (average of all environments) data to identify main effect QTLs for PHS resistance. CIM detected a total of 20 different PHS resistance QTLs on wheat chromosomes 1A, 2A, 2B, 2D, 3A, 3B, 3D, 4A, 4B, 4D, 5A, 7A and 7D (Fig. 3, Table 1 and Additional file 2: Table S3). Conversely, mixed-model based composite interval mapping (MCIM) identified a total of eleven QTLs (Additional file 2: Table S4). These included ten loci which were also detected using CIM and an additional minor QTL, *QPhs.lrdc-2B.2*, on chromosome 2B (Additional file 2: Table S4).

**Table 1:** Details of quantitative trait loci (QTLs) identified for pre-harvest sprouting (PHS) resistance

QTL Name	Chr	Position	Interval_**max**_	LOD_**max**_	Additive effect_**max**_	%***R***^***2***^_**max**_	Environment(s) CIM and MCIM	Closest marker and position			Donor allele
								Name	cM	nt	
*QPhs.lrdc-1A.1*	1A	27.80	27.6–30.8	2.55	0.32	5.0	Ithaca 2018, Pooled	*tplb0036l23_696*	27.59	16,056,699	T
*QPhs.lrdc-1A.2*	1A	60.40	58.4–64.0	6.66	0.63	14.0	Edmonton 2019	*Ku_c44068_601*	60.27	38,215,835	T
*QPhs.lrdc-1A.3*	1A	81.90	79.1–82.7	5.14	0.62	9.0	Lethbridge 2019, Pooled	*Kukri_c25215_124*	81.85	493,658,123	T
*QPhs.lrdc-2A*	2A	106.40	105.4–107.4	2.85	0.63	5.0	Lethbridge 2019	*BS00090569_51*	106.37	657,329,310	T
*QPhs.lrdc-2B.1*	2B	82.00	81.3–82.5^a^	5.01	1.43	10.0	Edmonton 2019, Pooled	*Tdurum_contig1653_190*	82.00	107,712,408	T
*QPhs.lrdc-2B.2*	2B	90.00	86.7–91.6^a^	NA	0.84	2.1	Edmonton 2019, Ithaca 2018, Lethbridge 2018, Lethbridge 2019, Pooled	*Ku_c69635_2786*	89.96	110,037,917	I
*QPhs.lrdc-2D.1*	2D	42.40	38.7–45.3	2.55	0.62	5.0	Ithaca 2018, Lethbridge 2019, Pooled	*wsnp_CAP12_c1503_764765*	42.37	37,927,516	T
*QPhs.lrdc-2D.2*	2D	101.30	98.8–102.2	3.40	0.34	6.0	Ithaca 2018	*Ex_c2115_3369*	104.08	437,187,123	T
*QPhs.lrdc-3A.1*	3A	8.20	6.3–9.3	12.00	1.16	19.0	Edmonton 2019, Ithaca 2018, Lethbridge 2018, Pooled	*Tdurum_contig83209_316*	7.61	1,253,899	T
*QPhs.lrdc-3A.2*	3A	19.60	18.6–27.9	4.82	0.84	9.0	Lethbridge 2019	*Ra_c9347_163*	19.54	35,795,572	T
*QPhs.lrdc-3B.1*	3B	1.70	0.7–2.3	2.89	0.53	4.0	Lethbridge 2018, Pooled	*BS00001400_51*	1.69	860,129	I
*QPhs.lrdc-3B.2*	3B	157.30	156.1–162.8	7.20	0.59	13.0	Ithaca 2018, Lethbridge 2019, Pooled	*BS00057988_51*	157.58	774,477,527	T
*QPhs.lrdc-3D.1*	3D	17.20	12.4–32.9	4.21	0.80	10.0	Lethbridge 2018	*wsnp_Ex_c7780_13254349*	18.67	346,008,587	I
*QPhs.lrdc-3D.2*	3D	122.20	107.6–138.4	6.18	0.48	12.0	Ithaca 2018, Pooled	*BS00067163_51*	117.07	573,581,940	T
*QPhs.lrdc-4A*	4A	45.60	45.3–48.9	6.14	0.78	9.0	Edmonton 2019, Ithaca 2018, Lethbridge 2018, Pooled	*wsnp_Ex_rep_c67799_66488792*	45.53	65,464,862	T
*QPhs.lrdc-4B*	4B	61.00	60.6–63.1	3.83	0.61	6.0	Lethbridge 2018, Pooled	*wsnp_Ex_c25373_34639805*	60.99	481,850,259	T
*QPhs.lrdc-4D*	4D	74.00	72.1–75.6	2.60	0.52	4.0	Lethbridge 2018	*BS00047797_51*	76.15	456,267,708	I
*QPhs.lrdc-5A.1*	5A	57.10	56.4–57.3	2.61	0.36	4.0	Pooled	*GENE-3502_206*	57.04	160,103,231	I
*QPhs.lrdc-5A.2*	5A	123.60	123.6–123.6	2.50	0.37	5.0	Edmonton 2019	*BS00065292_51*	123.50	556,978,174	T
*QPhs.lrdc-7A*	7A	192.20	190.8–193.9	2.65	0.49	4.0	Ithaca 2018, Lethbridge 2018, Lethbridge 2019, Pooled	*TA003093–1305*	192.01	637,205,996	T
*QPhs.lrdc-7D*	7D	89.20	70.5–106.6	6.21	1.20	18.0	Lethbridge 2019, Pooled	*D_GCE8AKX02ILA1U_88*	79.82	56,638,676	I

Note - *Chr* chromosome, *Interval*_*max*_ QTL interval (cM) calculated using markers identified in composite interval mapping (CIM) or mixed-model based composite interval mapping (MCIM) based on all the environments; ‘cM’ and ‘nt’ positions are based on AAC Innova/AAC Tenacious linkage map and IWGSC RefSeq v.2 physical map/genome, respectively

^a^QTL interval based on MCIM results only; LOD_max_, Additive effect_max_ and %*R*^*2*^_max_: highest score reported in any single environment or pooled data, additive effect is shown as absolute value; NA: QTL detected using MCIM only and no LOD score was calculated; Donor allele: T – AAC Tenacious, I – AAC Innova

Phenotypic variation (*R*^*2*^) explained by twenty main-effect loci detected using CIM ranged from 4.0% (*QPhs.lrdc-3B.1*, *QPhs.lrdc-4D*, *QPhs.lrdc-5A.1* and *QPhs.lrdc-7A*) to 19.0% (*QPhs.lrdc-3A.1*) (Fig. 3 and Table 1). The LOD score of individual QTLs ranged from 2.50 (*QPhs.lrdc-5A.2*) to 12.00 (*QPhs.lrdc-3A.1*) and the additive effect (AE) ranged from 0.32 (*QPhs.lrdc-1A.1*) to 1.43 (*QPhs.lrdc-2B.1*) (Fig. 3 and Table 1). Only seven of the total identified loci (located on chromosomes 1A, 2B, 3A, 3B, 3D, and 7D; Table 1) explained ≥10% *R*^*2*^ for PHS (Fig. 3 and Table 1) and were considered major QTLs. However, based on the LOD score (≥5.0), the AE (≥1.0) and the *R*^*2*^ (≥10.0) values, three QTLs (*QPhs.lrdc-2B.1*, *QPhs.lrdc-3A.1* and *QPhs.lrdc-7D*) were narrowed down to be highly effective and major QTLs.

Notably, where in each individual environment there was at least one major QTL detected (Fig. 3 and Table 1), together four QTLs (*QPhs.lrdc-2B.2*, *QPhs.lrdc-3A.1*, *QPhs.lrdc-4A* and *QPhs.lrdc-7A*) were identified in at least three environments as well as in the pooled data (Fig. 3 and Table 1). While PHS resistance alleles at around three quarters of the total detected loci were contributed by AAC Tenacious, AAC Innova, the susceptible parent, also contributed resistance alleles at six QTLs, which included two major loci, *QPhs.lrdc-3D.1* and *QPhs.lrdc-7D* (Fig. 3 and Table 1).

#### Digenic epistasis interaction

Two of the above mentioned main effect QTLs on chromosomes 1A (*QPhs.lrdc-1A.1*) and 7A (*QPhs.lrdc-7A*) were found to be involved in digenic epistasis interaction (Fig. 3, Table 1 and Additional file 2: Tables S4 and S5). Notably, although these QTLs did not contribute much (*R*^*2*^: 4 to 5% and AE: 0.32 to 0.49) individually, their epistatic interaction indicates that the parental two-locus genotypes had additional negative effect on sprouting (AA value: − 0.24, phenotypic variance: 4.9) (Additional file 2: Table S5).

#### Combined effect of major PHS resistance QTLs on sprouting

Pooled PHS and single nucleotide polymorphism (SNP) genotyping data of all the DH lines were analyzed for the linked markers (*Ku_c44068_601*, *Tdurum_contig1653_190*, *Tdurum_contig83209_316*, *BS00057988_51*, *wsnp_Ex_c7780_13254349*, *BS00067163_51*, and *D_GCE8AKX02ILA1U_88*) for all major QTLs (*QPhs.lrdc-1A.2*, *QPhs.lrdc-2B.1*, *QPhs.lrdc-3A.1*, *QPhs.lrdc-3B.2*, *QPhs.lrdc-3D.1*, *QPhs.lrdc-3D.2* and *QPhs.lrdc-7D*) detected in this study. PHS data of DH lines having the same genotypic profile for each group of markers were pooled, and mean PHS and standard deviation were estimated. Mean PHS of each group of DH lines, or unique line with a single QTL and combination of QTLs, were plotted as bar plots and line graph (Additional file 3: Fig. S2). DHs across environments showed a gradual increase in PHS resistance with increasing number of QTLs (Additional file 3: Fig. S2). Notably, a highly resistant phenotype was observed only when at least five major QTLs were present together (Additional file 3: Fig. S2). Conversely, some lines were relatively more susceptible than other lines in their group even in the presence of resistance alleles at five QTLs, which indicates that other factors also influence PHS resistance.

**Fig. 4:**
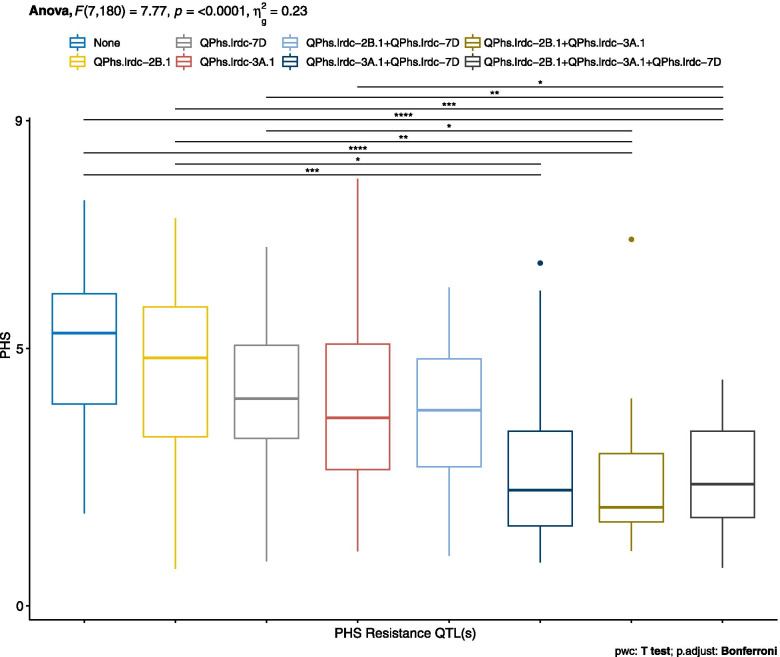
Boxplot distributions of pre-harvest sprouting (PHS) score in doubled haploid (DH) population. All DH lines produced from the cross AAC Innova/AAC Tenacious were grouped into eight different genotypic (QTL) classes based on three major QTLs *QPhs.lrdc-2B.1*, *QPhs.lrdc-3A.1* and *QPhs.lrdc-7D*. Effects of positive alleles of single QTL and their combinations on average PHS score are represented alongside negative alleles at all three loci using the pooled phenotypic data (average of all environments). Statistically significant differences among QTLs/QTL combinations were calculated by ANOVA, pairwise T test with Bonferroni corrections and shown by asterisk. Quartiles and medians are represented by boxes and continuous lines, respectively. Whiskers extend to the farthest points that are not outliers, whilst outliers are shown as dots

To identify the most effective QTL and to assess the specific effect of QTLs, only three main major and effective QTLs, namely *QPhs.lrdc-2B.1*, *QPhs.lrdc-3A.1* and *QPhs.lrdc-7D*, were selected. Based on the genotyping profile of these QTLs, the DH lines were categorized into eight different genotypic classes (Additional file 2: Table S6), irrespective of the PHS resistance alleles at other detected/undetected loci. Mean PHS of each group of DH lines for each individual QTL and group of QTLs was plotted as boxplots (Fig. 4). It was observed that while individually, *QPhs.lrdc-3A.1* contributed maximum PHS resistance, a gradual decrease in sprouting was observed with increasing number of QTLs (Fig. 4) indicating the cumulative AE. However, statistically significant differences in mean PHS of the susceptible vs resistant groups were observed only when at least two QTLs were present, particularly *QPhs.lrdc-3A.1* and one other QTL (Fig. 4).

#### QTL genome locations and comparisons with previously identified QTLs/genes

Based on all the SNP markers mapped to the QTL regions in this study, physical positions of all the markers on the wheat reference genome (IWGSC RefSeq v2.0) were detected (Additional file 2: Tables S7, S8). This led to the identification of physical intervals of all the QTLs on wheat chromosomes (Table 2). Results from a total of 32 previously published studies and various numbers of other genes from different online sources (Additional file 2: Table S9) were assessed to check if they overlap physical intervals (on reference genome) of QTLs detected in this study. We found that 13 of the 21 main effect-loci identified in this study appeared to shared chromosome positions where at least one QTL has been previously identified in other wheat genotype(s) (Table 2). The remaining eight QTLs appear to be new and were identified for the first time in this study. These new QTLs also include two major QTLs, *QPhs.lrdc-2B.1* and *QPhs.lrdc-3B.2*, and a most stable but minor QTL, *QPhs.lrdc-2B.2,* which was identified across environments and in the pooled data. AAC Tenacious contributed resistance at these two major QTLs, while AAC Innova at minor QTL *QPhs.lrdc-2B.2* (Tables 1 and 2).

**Table 2:** Details of previously identified pre-harvest sprouting resistance quantitative trait loci (QTLs) and candidate genes

QTL identified from AAC Innova/AAC Tenacious population				Previously identified QTL(s)^**c**^					Candidate gene(s)^**e**^	
Name	Chr	Genetic interval_**max**_^**a**^	Physical interval_**max**_^**b**^	QTL(s)/marker(s)	Genotype	Origin	Reference	Ped^**d**^	Name	Position^**f**^
*QPhs.lrdc-1A.1*	1A	27.6–30.8	15,899,537–25,067,873	None	–	–	–	–	None	–
*QPhs.lrdc-1A.2*	1A	58.4–64.0	30,503,486–83,683,139	*QPhs.ccsu-1A.1*	HD2329	India	[58]	T	None	–
*QPhs.lrdc-1A.3*	1A	79.1–82.7	473,329,664–499,633,978	*Xbarc145*	AM panel	Others	[70]	NI	None	–
*QPhs.lrdc-2A*	2A	105.4–107.4	651,599,654–681,046,848	*QPhs.ccsu-2A.3*	SPR8198	India	[58]	NI	None	–
				*Qphs.hwwg-2A.1*	Danby	USA	[12]	T		
*QPhs.lrdc-2B.1*	2B	81.3–82.5	107,349,822–112,188,108	None	–	–	–	–	None	–
*QPhs.lrdc-2B.2*	2B	86.7–91.6	105,533,857–118,803,206	None	–	–	–	–	None	–
*QPhs.lrdc-2D.1*	2D	38.7–45.3	35,052,295–64,499,689	*Qphs.sau-2D*	RSP	China	[56]	NS	*Ppd-D1*	36,205,433
*QPhs.lrdc-2D.2*	2D	98.8–102.2	437,187,123–475,829,319	None	–	–	–	–	None	–
*QPhs.lrdc-3A.1*	3A	6.3–9.3	1,241,792–12,114,082	*QPhs.pseru-3A/TaPHS1*	Rio Blanco	USA	[42, 49, 50]	T	*MFT-A1b*	7,362,511
				*QPhs.ocs-3A.1, QDor-3A, MFT-3A*	Zenkoujikomugi	Japan	[2, 57, 59]	NS		
				*Qphs.hwwg-3A.1*	Danby	USA	[12]	T		
				*wsnp_Ex_rep_c67702_66370241*, *wsnp_Ra_c2339_4506620*, *Xbarc57.2*	AM panel	Others	[70]	NI		
*QPhs.lrdc-3A.2a*	3A	18.6–27.9	17,351,806–527,206,323	*QPhs.ocs-3A.1*, *QPhs.ocs-3A.2*	Zenkoujikomugi	Japan	[60]	NS	*AGO802A*	239,707,025
				*QPhs.ccsu-3A.1*	SPR8198	India	[58]	NI		
*QPhs.lrdc-3B.1*	3B	0.7–2.3	858,443–6,086,589	*QGi.crc-3B*	AC Domain	Canada	[71]	TI	*MFT-3B-1*	4,443,008
*QPhs.lrdc-3B.2*	3B	156.1–162.8	774,475,703–775,489,185	None	–	–	–	–	None	–
*QPhs.lrdc-3D.1*	3D	12.4–32.9	4,376,769–529,546,644	*QPhs.cnl-3D.1*	Cayuga	USA	[72]	T	*AGO802D*, *HUB1*, *TaVp1-D1*	181,668,030, 477,351,285, 526,085,089
				*QGi.crc-3D*	AC Domain	Canada	[71]	TI		
				*Xbarc376*	AM panel	Others	[70]	NI		
*QPhs.lrdc-3D.2*	3D	107.6–138.4	566,481,133–598,343,827	*QPhs.inra-3D*	Renan	France	[34]	T	*TaMyb10-D1*	572,151,928
				*QGi.crc-3D*	AC Domain	Canada	[71]	T		
				*TaMyb10-D1*	AM panel	Others	[70]	NI		
*QPhs.lrdc-4A*	4A	45.3–48.9	56,469,956–543,554,202	*Phs1*	OS21–5	Japan	[51]	T	None	–
				*Phs1*	Leader	Canada	[52]	T		
				*QPhs.ocs-4A.1, QDor-4A*;	Zenkoujikomugi	Japan	[57, 59]	NS		
				Sprouting QTL	Opata	Mexico	[61]	T		
*QPhs.lrdc-4B*	4B	60.6–63.1	439,276,911–569,339,659	*QPhs.ocs-4B.1*	Chinese Spring	China	[57]	NS	None	–
*QPhs.lrdc-4D*	4D	72.1–75.6	398,807,986–456,267,808	None	–	–	–	–	None	–
*QPhs.lrdc-5A.1*	5A	56.4–57.3	160,103,131–399,974,559	*Qphs.hwwg-5A.1*	Danby	USA	[12]	TI	None	–
*QPhs.lrdc-5A.2*	5A	123.6–123.6	556,976,254–558,357,114	None	–	–	–	–	None	–
*QPhs.lrdc-7A*	7A	190.8–193.9	620,094,248–639,730,768	None	–	–	–	–	None	–
*QPhs.lrdc-7D*	7D	70.5–106.6	46,061,671–102,506,349	*QFn.crc-7D*	AC Domain	Canada	[73]	TI	None	–

Note - *Chr* Chromosome

^a,b^Genetic (AAC Innova/AAC Tenacious) and physical/genomic (IWGSC RefSeq v.2) intervals of QTL calculated based on markers identified in all the environments

^c^Previously identified QTL(s) located in the genomic interval of respective QTL identified in this study

^d^Pedigree information: T – AAC Tenacious shared pedigree with donor cultivar/genotype of respective QTL(s), TI – both AAC Tenacious and AAC Innova shared pedigree with donor cultivar/genotype of respective QTL(s), NS – neither shared with AAC Tenacious nor with AAC Innova, NI – no information or unknown pedigree

^e^cloned PHS resistance or other candidate gene(s) located in physical/genomic interval of respective QTL

^f^physical/genomic positions of candidate gene(s) based on IWGSC RefSeq v.2; −- not applicable

Comparative analyses of the genomic intervals of QTLs detected in this study with that of previously identified and cloned PHS resistance genes identified several candidate genes in QTL regions (Table 2). These include *Ppd-D1b* (in QTL interval *QPhs.lrdc-2D.1*), *MFT-A1b* (in QTL interval *QPhs.lrdc-3A.1*) and *AGO802A* (in QTL interval *QPhs.lrdc-3A.2*) on chromosome 3A, *MFT-3B-1* (in QTL interval *QPhs.lrdc-3B.1*) on chromosome 3B, and *AGO802D* and *TaVp1-D1* (in QTL interval *QPhs.lrdc-3D.1*) and *TaMyb10-D1* (in QTL interval *QPhs.lrdc-3D.2*) on chromosome 3D (Table 2).

**Fig. 5:**
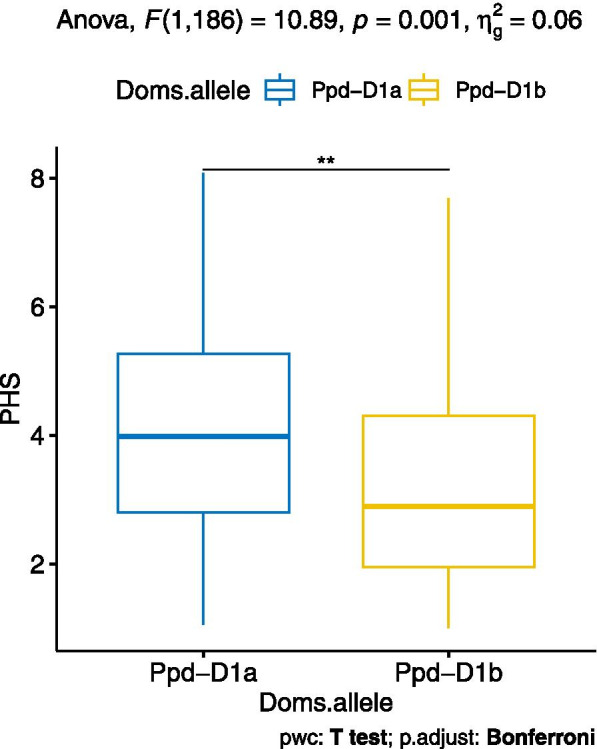
Boxplot distributions of pre-harvest sprouting (PHS) score in population divided into photoperiod-insensitive vs -sensitive groups. All DH lines produced from the cross AAC Innova/AAC Tenacious were grouped into two genotypic classes, photoperiod-insensitive (*Ppd-D1a*) and -sensitive (*Ppd-D1b*), based on the domestication allele of the photoperiod gene *Ppd-D1*. *Ppd-D1* is an important candidate gene for *QPhs.lrdc-2D.1* QTL on chromosome 2D. Effects of domestication alleles of *Ppd-D1* on average PHS score are presented using the pooled phenotypic data (average of all environments). Photoperiod-sensitive allele *Ppd-D1b* significantly reduced PHS in AAC Innova/AAC Tenacious DH population. Statistically significant difference between alleles is calculated by ANOVA, pairwise T test with Bonferroni corrections and shown by asterisk. Quartiles and medians are represented by boxes and continuous lines, respectively. Whiskers extend to the farthest points that are not outliers

One of the above candidate genes, *Ppd-D1*, a photo-response and domestication gene, was assessed for its association with PHS resistance and days to anthesis (DTA). Genetically, *Ppd-D1* was mapped to *QPhs.lrdc-2D.1* interval within 1.61 cM of the closely linked SNP marker *wsnp_CAP12_c1503_764765* (Table 1 and Additional file 2: Table S7). It was observed that the AAC Tenacious derived photoperiod-sensitive allele *Ppd-D1b* significantly reduced pre-harvest sprouting in AAC Innova/AAC Tenacious population, irrespective of other genes/QTLs (Fig. 5). On the other hand, DTA showed weak negative association (*r* − 0.20) with PHS resistance.

A detailed AAC Tenacious pedigree chart with information of different PHS-resistant sources was generated (Additional file 4: Fig. S3). Interestingly, AAC Tenacious has several PHS-resistant bread wheat landraces/genotypes [Akakomugi (landrace, Japan), Button (cultivar, Kenya), Crimean (landrace, USA), Frontana (cultivar, Brazil), Hard Red Calcutta (landrace, India), Kenya-Farmer (cultivar, Kenya), Kenya 9 M-1A-3 (breeding line, Kenya), Kenya-U (breeding line, Kenya), Ostka Galicyjska (landrace, Poland), RL2265 (breeding line, Canada), RL4137 (breeding line, Canada), Thatcher (cultivar, USA) and Turco (landrace, Brazil)] and a durum cultivar Iumillo (USA) in its parentage as progenitors (Additional file 4: Fig. S3). A number of pedigrees (Additional file 5) of the cultivars/genotypes including AAC Innova and that previously reported to possesses PHS resistance QTL(s)/gene(s) in the same chromosomal regions where QTLs have been reported in this study were also searched. It was observed that AAC Tenacious or AAC Innova shared their pedigrees with at least 9 (out of all the cultivars/genotypes with known pedigrees) different PHS-resistant cultivars/genotypes (AC Domain, Leader, Renan, HD2329, OS21–5, Opata, Cayuga, Danby and Rio Blanco) from six different countries (Canada, France, India, Japan, Mexico and the USA) (Table 2). AAC Tenacious and AAC Innova do not share their pedigrees with two resistant cultivars/genotypes, RSP (from China) and Zenkoujikomugi (from Japan) and susceptible landrace Chinese Spring (from China) (Table 2).

## Discussion

PHS is a serious threat to wheat production in many growing areas, particularly where late seasonal rainfall occurs during harvest. In recent years, it has become more frequent due to uncertain weather conditions linked with climate change [53]. Integrating PHS resistance in modern wheat cultivars is a major breeding objective in many countries including Australia, Canada, China, Japan and USA [53]. Seed dormancy is considered the dominant factor in controlling PHS resistance in cereals [7]; however, highly dormant seed is considered to be a limiting factor in obtaining uniform germination and early seedling establishment [62]. Thus, to meet the contradictory demands of PHS resistance and proper germination when required, breeding programs need to incorporate alternate mechanisms into modern cultivars [2] such as moderate dormancy in combination with modified spike morphology.

AAC Tenacious is a highly PHS resistant, tall, photoperiod-sensitive and red-grained Canadian wheat cultivar [68]. It also possesses a gibberellic acid (GA)-sensitive tall plant height allele *Rht-B1a* and the brassinosteroid-sensitive tall plant height allele *Rht8a* [74] at *Xgwm261* locus [75]. Above features make AAC Tenacious assessment important, not only for red-grain associated factors, but also for alternate physiological mechanisms including photoperiodic response. To this objective, AAC Tenacious was crossed with the white-grained, semi-dwarf, soft-textured and photoperiod-insensitive Canadian wheat cv AAC Innova [76] to develop a diverse DH mapping population.

Parents of this population showed contrasting PHS phenotypes and the DH population was skewed toward resistance in the three out of four environments (Fig. 2), suggesting the involvement of multiple large-effect genes/QTLs responsible for PHS resistance. As indicated by the phenotypic analysis, this population segregated for seven major QTLs along with 14 minor QTLs (Table 1). This can be explained by the parentage of AAC Tenacious which is composed of several PHS-resistant sources (Additional file 4: Fig. S3) such as Thatcher, Hard Red Calcutta, Ostka Galicyjska, RL4137, etc. [54]. Moreover, it is known that seed dormancy is controlled by multiple genes/QTLs distributed over all 21 wheat chromosomes [59]. The contributions of such multiple PHS resistance QTLs from a single cultivar have also been reported previously [58].

Together, 21 PHS resistance loci were identified in the present study including 15 from AAC Tenacious and six from AAC Innova (Table 1). These also included seven major QTLs, each individually explained ≥10% phenotypic variation (PV) (Table 1). Notably, most of the other QTLs, that explained < 10% PV, were detected in single environments and considered to be small effect [77] suggestive [78] QTLs. Major PHS resistance QTLs were *QPhs.lrdc-1A.2*, *QPhs.lrdc-2B.1*, *QPhs.lrdc-3A.1*, *QPhs.lrdc-3B.2*, *QPhs.lrdc-3D.1*, *QPhs.lrdc-3D.2* and *QPhs.lrdc-7D* (Fig. 3 and Table 1). Broadly, only three QTLs (*QPhs.lrdc-2B.1*, *QPhs.lrdc-3A.1* and *QPhs.lrdc-7D*) were considered highly effective QTLs based on the high LOD score, AE and the explained PV. Interestingly, AAC Tenacious contributed resistance alleles at all these three loci.

On the other hand, four QTLs (*QPhs.lrdc-2B.2*, *QPhs.lrdc-3A.1*, *QPhs.lrdc-4A* and *QPhs.lrdc-7A*) were detected in at least three environments as well as in the pooled data. These QTLs are considered the most stable QTLs identified in this study; however, *QPhs.lrdc-3A.1* is the only major QTL (explained up to 19.0% PV) among the four loci. Remaining 17 loci were detected in either ≤2 environments (with or without pooled data) or just in the pooled data. These results suggest a high environmental effect on expression of PHS resistance, which is expected for a quantitative trait [58] influenced by several environmental and genetic factors [2, 4, 6].

Despite the number of QTLs identified previously from different genotypes (reviewed in [1]), 8 QTLs (*QPhs.lrdc-1A.1*, *QPhs.lrdc-2B.1*, *QPhs.lrdc-2B.2*, *QPhs.lrdc-2D.2*, *QPhs.lrdc-3B.2*, *QPhs.lrdc-4D*, *QPhs.lrdc-5A.2* and *QPhs.lrdc-7A*) identified in this study are reported for the first time (Table 2). These include a relatively stable major QTL *QPhs.lrdc-3B.2* (detected in Ithaca 2018, Lethbridge 2019 and the pooled data) derived from AAC Tenacious and do not seem to be homoeo-QTL or paralogues. This reinforces the importance of AAC Tenacious in dissecting PHS resistance.

All the important QTLs are discussed first in greater details followed by others below. *QPhs.lrdc-3A.1*, a very important QTL, explained the most PV (up to 19.0%) of PHS trait and had the highest LOD score of 12.0. The AAC Tenacious allele at this locus had 1.16 AE which reduces sprouting by around 13.0%. This QTL was detected in Edmonton 2019, Ithaca 2018, Lethbridge 2018 and the pooled data, and is considered one of the most stable QTL identified in this study. Interestingly, a number of QTLs, such as *QPhs.pseru-3A*/*TaPHS1*, *QPhs.ocs-3A.1*, *QDor-3A*, *Qphs.hwwg-3A.1*, from cultivars like Rio Blanco and Danby (USA) and Zenkoujikomugi (Japan) [2, 12, 42, 49, 50, 57, 59], and a number of markers, such as *wsnp_Ex_rep_c67702_66370241*, *wsnp_Ra_c2339_4506620*, and *Xbarc57.2*, from diverse winter wheat association mapping panels [70] have been mapped to the same overlapping region as *QPhs.lrdc-3A.1*. Notably, AAC Tenacious shares its pedigree with US cvs Rio Blanco and Danby, but Japanese cv Zenkoujikomugi is unrelated to AAC Tenacious. Unexpectedly, the presence of this QTL in different cultivars with related/unrelated pedigrees showed the robustness and usefulness of this QTL for breeding PHS resistant wheat in different genetic backgrounds. A causal gene, *MFT-A1b*/*TaPHS1* (*Mother of FT* and *TFL1*), has also been cloned from this region previously [2]. Comparative analysis showed that this QTL region, along with a 3B QTL region are syntenic to chromosomal regions harbouring *TaMFT*-like genes. *TaMFT* is a homologue of the Arabidopsis *MFT* gene which controls embryo-imposed seed dormancy and also regulates ABA and GA signal transduction [2, 79]. These genes are members of the plant phosphatidylethanolamine binding protein (PEBP) family and are phylogenetically related to subfamilies, *FLOWERING LOCUS T* (*FT*)-like and *TERMINAL FLOWER1* (*TFL1*)-like [80]. Where these genes show seed-specific expression [80], their ancestral relative *FT* and *TFL1*, two flowering genes, act as molecular switches for reproductive development [81] in Arabidopsis, thus implying *QPhs.lrdc-3A.1* to be a very important QTL.

Two other important QTLs detected on homeologous group 3 chromosomes were *QPhs.lrdc-3D.1* and *QPhs.lrdc-3D.2*. Both of these QTLs explained similar PV (up to 10.0 and 12.0%, respectively) and had similar LOD scores (up to 4.21 and 6.18, respectively), but they differed significantly for AE (up to 0.80 and 0.48, respectively). Moreover, while the resistance allele at *QPhs.lrdc-3D.1* was contributed by AAC Innova, resistance allele at *QPhs.lrdc-3D.2* was contributed by AAC Tenacious. *QPhs.lrdc-3D.1* was mapped to the same interval as at least three previously reported QTLs, including PHS resistance QTL *QPhs.cnl-3D.1* from US cv Cayuga [72], germination index QTL *QGi.crc-3D* from Canadian cv AC Domain [71], and a QTL at marker locus *Xbarc376* in a germplasm line [70]. Interestingly, AAC Innova also shares its pedigree with AC Domain and US cv Cayuga, which have common PHS-resistant source landraces in their lineage like Hard Red Calcutta.

We have located homologs of three important genes, namely *AGO802D*, *Reduced dormancy4* (*RDO4*)/*Histone Monoubiquitination1* (*HUB1*) and *Viviparous-1 (Vp1)*, in the physical interval of *QPhs.lrdc-3D.1*. All these candidate genes are known to influence seed dormancy through ABA-synthesis and -signal transduction pathway [1, 5, 31, 33, 43, 45]. Furthermore, the 3B homolog of *AGO802D* in wheat and barley, and *HUB1* in Arabidopsis are believed to be involved in epigenetic changes that have a role in seed dormancy [5, 43, 45]. The role of ARGONAUTE (AGO) proteins in the DNA silencing through the RNA dependant DNA methylation pathway have previously been linked with seed dormancy in wheat [5]. Singh et al. [5] located three *AGO802* genes on group 3 chromosomes of wheat and found that *AGO802-B* on chromosome 3B was associated with seed dormancy in six Canadian wheat cultivars/genotypes. During the present study, we found that the QTL intervals *QPhs.lrdc-3A.2* (on chromosome 3A) and *QPhs.lrdc-3D.1* (on chromosome 3D) are syntenic to the physical interval of *AGO802-A* and *AGO802-D*, respectively. However, we could not locate *AGO802-B* to the PHS resistance QTL interval on 3B. It would be useful if the role of the two *AGO* genes could be confirmed in the PHS resistance of AAC Tenacious. Another important candidate gene is histone methyltransferase *RDO4/HUB1,* which positively regulates expression of *Delay of germination 1* (*DOG1*), a gene which encodes a member of a plant specific protein family with a domain shared by the D class bZIP DNA-binding proteins [45, 82]. *Vp1* is another candidate in this QTL interval that encodes a transcription factor that regulates late embryo development in bread wheat [1]. It has been previously linked with seed dormancy and PHS resistance (reviewed in [1]). Expression of *Vp1* in wheat embryos has been positively correlated with ABA sensitivity and degree of seed dormancy [31, 33]. Splicing of the *Vp1* gene in wheat resulted in susceptibility to PHS [33]. The *TaVp1* genes were previously mapped around 30 cM away from *R* loci on group 3 chromosomes [29, 34, 35]. *Vp1* could be an important regulator of PHS/seed dormancy in this QTL region of AAC Tenacious.

Second 3D QTL, *QPhs.lrdc-3D.2*, mapped to the 3D genomic interval where at least three QTLs have been previously mapped from different cultivars. These include PHS resistance QTL *QPhs.inra-3D* from French cv Renan [34], germination index QTL *QGi.crc-3D* from Canadian cv AC Domain [71] and *TaMyb10-D1* using diverse germplasm [70]. AAC Tenacious shares its pedigree with AC Domain and the French cv Renan, both of which had Thatcher as a common ancestor. Moreover, the grain color gene *TaMyb10-D1* was also located to the genomic interval of this QTL. It seems that *QPhs.lrdc-3D.2* was associated with the expression of *TaMyb10-D1* that regulates the key enzymes in the flavonoid pathway [58].

The seed coat restrict germination by its mechanical resistance to radicle protrusion or being impermeable to water and/or oxygen [83]. Seed coat properties, particularly the presence of phenolic compounds, positively correlate with seed coat color (reviewed in [1]). Red-grained wheat genotypes exhibit a wide range of seed dormancy and are more resistant to PHS than white-grained cultivars [84]. Grain color (GC) was found to be associated with seed dormancy and PHS resistance in many wheat cultivars previously and is controlled by the *R-1* genes located on long arms of chromosomes 3A, 3B, and 3D (reviewed in [1]), [84, 85]. *Myb*-type transcription factor loci (*Tamyb10-A1*, *Tamyb10-B1*, and *Tamyb10-D1*), which act as transcriptional activators for flavonoid synthesis pathway genes, have previously been found associated with seed dormancy and PHS resistance and are located in the same regions as the *R* genes [1, 27, 29, 84, 85]. Himi et al. [85] also confirmed the three *Tamyb10–1* genes on chromosomes 3AL, 3BL, and 3DL as candidate genes underlying the *R-1* loci for wheat grain color. Since the AAC Innova/AAC Tenacious DH population also segregated for grain color, *TaMyb10-D1* could be an important gene in *QPhs.lrdc-3D.2* region.

Another QTL identified during this study is *QPhs.lrdc-4A*. Though it explained 9.0% PHS PV but was detected in Edmonton 2019, Ithaca 2018, Lethbridge 2018 and the pooled data. It had an AE up to 0.78 and a LOD score up to 6.14 (Table 1). The AAC Tenacious allele at this QTL reduced PHS by around 8.7%. A number of QTLs, such as the major QTL *Phs1* from Canadian cv. Leader and Japanese line OS21–5 [51, 52], *QPhs.ocs-4A.1* and *QDor-4A* from Japanese cv. Zenkoujikomugi [57, 59], and a sprouting QTL from Mexican cv. Opata [61] have been mapped to the same region as of *QPhs.lrdc-4A*. AAC Tenacious shares its pedigree with Leader, OS21–5 and Opata, but not with Zenkoujikomugi. The major 4A QTL *Phs1* in wheat is an ortholog of *SD2*(*Qsd2-AK*) in barley [52, 86]. Torada et al. [52] identified a *mitogen-activated protein kinase kinase 3* (*MKK3*) gene (or *TaMKK3-A*) as a candidate gene for the seed dormancy QTL *Phs1* on chromosome 4A in wheat. Abe et al. [86] developed a triple (for all homeologous loci)-knockout mutant of the *Qsd1*, another dormancy locus in barley, using CRISPR/Cas9 in wheat cv Fielder which also showed longer dormancy than the wild-type plants. However, a BLAST search of the complete mRNA sequence (GenBank: LC091369.1) of candidate gene *TaMKK3-A* resulted in no perfect match on chromosome 4A of IWGSC RefSeq v2.0 of wheat. Additional experiments are required to confirm the association of *TaMKK3-A* with *QPhs.lrdc-4A*.

Four other loci of great importance identified in this study are *QPhs.lrdc-1A.2*, *QPhs.lrdc-2B.1*, *QPhs.lrdc-3B.2* and *QPhs.lrdc-7D*. Out of these, *QPhs.lrdc-1A.2* explained up to 14.0% PV of PHS and also had a high LOD score of 6.7 (Table 1). Although the AE of this QTL was only 0.63, it still reduced PHS by around 7.0%. It mapped to the same interval where at least one QTL, *QPhs.ccsu-1A.1*, has been previously identified and mapped from Indian bread wheat cv HD2329 [58]. HD2329 also shared its pedigree with AAC Tenacious and traces back to different common cultivars such as Thatcher, Marquis, Hard Red Calcutta, Frontana, etc.

*QPhs.lrdc-2B.1* explained 10.0% of PHS PV, had a maximum AE (up to 1.43) on PHS and was detected in Edmonton 2019 and the pooled data (Table 1). The AAC Tenacious allele at this QTL reduced PHS by around 16.0%. Interestingly, this QTL is being reported for the first time and does not seem to be homoeo-QTL or paralogue.

*QPhs.lrdc-3B.2* explained up to 13.0% PV and had an AE of 0.59 detected at a high LOD score of 7.20. The resistance allele at this QTL was contributed by AAC Tenacious and reduced PHS up to 6.5%. Like *QPhs.lrdc-2B.1*, it is a new PHS resistance QTL being reported for the first time. It was detected in Ithaca 2018, Lethbridge 2019, and the pooled data, and like *QPhs.lrdc-2B.1*, is considered a new, major and relatively stable QTL. Resistance allele at this QTL was contributed by AAC Tenacious.

*QPhs.lrdc-7D* explained up to 18.0% PV and had a LOD score > 6.0 and an AE of 1.20. Interestingly, the resistance allele at this locus was contributed by AAC Innova and it was detected in Lethbridge 2019 and the pooled data. The AAC Innova allele at this locus reduced sprouting by around 13.0%. A falling number QTL, namely *QFn.crc-7D*, in the same region of this QTL on chromosome 7D has been previously reported from the Canadian wheat cultivar AC Domain [73]. The discovery of this QTL in AAC Innova is not unexpected as both AAC Innova and AC Domain share their early Canadian wheat lineage through the PHS resistance source cv Hard Red Calcutta [54].

QTLs *QPhs.lrdc-1A.3* (AE: up to 0.62, LOD score: up to 5.14 and PVE: up to 9.0%) and *QPhs.lrdc-3A.2* (AE: up to 0.84, LOD score: up to 4.82 and PVE: 9.0%) are also important. QTLs/markers have been previously repeatedly mapped in genomic regions of these QTLs utilizing diverse germplasm, and Indian and Japanese lines/cvs with either no information or unrelated pedigrees (Table 2) [58, 60, 70]. This indicates that the identified QTLs can be utilized in different genetic backgrounds/geographical areas for improving PHS as an adaptive trait.

In addition to the above-mentioned QTLs, a number of other QTLs such as *QPhs.lrdc-2A*, *QPhs.lrdc-2D.1*, *QPhs.lrdc-3B.1*, *QPhs.lrdc-4B* and *QPhs.lrdc-5A.1* had relatively less effect on PHS resistance (Table 1) and were considered minor suggestive loci [77, 78]. However, PHS resistance QTLs/genes have been previously identified in genomic regions of these QTLs from different genotypes (Table 2) such as SPR8198 (India, unknown pedigree), Danby (USA, shared pedigree with AAC Tenacious), RSP (China; PHS resistance derived from *Aegilops tauschii* [87]), AC Domain (Canada, shared pedigree with AAC Tenacious) and Chinese Spring (China, susceptible [57, 88] cultivar with unrelated pedigree) [12, 56–58, 71]. Therefore, these regions can also be of regional and/or global utility. For instance, *Ppd-D1*, a photoperiod response and domestication gene, was located to the genomic interval of *QPhs.lrdc-2D.1*. Genotyping of the whole DH population with functional marker of domestication/photoperiod response gene *Ppd-D1* showed that AAC Innova had a photoperiod-insensitive allele *Ppd-D1a*, while AAC Tenacious had the photoperiod-sensitive allele *Ppd-D1b* [75]. It was observed that the AAC Tenacious derived photoperiod-sensitive allele *Ppd-D1b* significantly reduced pre-harvest sprouting in the AAC Innova/AAC Tenacious population, irrespective of other genes/QTLs (Fig. 5). This gene can be utilized to improve PHS resistance using marker-assisted selection in wheat cultivars for geographical areas where longer photoperiods occur over wheat growing season.

Knowing that the maturity date, which can be affected by *Ppd-D1* [89], would affect the PHS resistance [90], it is intriguing to know whether PHS resistance in *QPhs.lrdc-2D.1* region is a function of maturity date or a direct effect of *Ppd-D1*. However, we did not record the maturity date on this population, DTA data was available from one [75] ﻿of our previous studies on this population and used for correlation coefficient (*r*) analysis with PHS data. DTA showed weak negative (*r* − 0.20) association with PHS. Moreover, our group recently mapped a DTA QTL [75] to same chromosomal region as *QPhs.lrdc-2D.1* in AAC Innova/AAC Tenacious population. Although it is difficult to draw firm conclusions about the effect of maturity date utilizing available datasets without further investigation, our results and previous findings [75] suggest that PHS resistance at *QPhs.lrdc-2D.1* is perhaps influenced by DTA and the later conditions this population was exposed to during grain development stages. It is known that a semi-dominant mutation in the promoter region of the *Ppd-D1* gene, which transforms long day wheat to day neutral (photoperiod insensitive) and provides adaptation to a wide range of environments, was widely used in the “green revolution” [91]. Our results are in agreement with previous observations that domestication played a role in the loss of seed dormancy and changes in photoperiod sensitivity, two of the several common features of “domestication syndrome” [63, 66, 92–94].

## Conclusion

This study showed the complexity of PHS resistance in AAC Tenacious. Multiple PHS resistance loci, including some major QTLs, were identified from AAC Tenacious in comparison to only a few from AAC Innova. However, AAC Innova also contributed two major QTLs with most of the QTLs being unstable (detected in single environment) except minor QTL *QPhs.lrdc-2B.2*. Therefore, pyramiding of major PHS resistance loci from both parents as source cultivars could significantly improve PHS resistance in future wheat cultivars. Moreover, around two-third (13) of identified loci were mapped to the chromosomal regions of previously identified QTLs. These common regions included some QTLs detected repeatedly during previous studies, such as *QPhs.lrdc-3A.1* and *QPhs.lrdc-4A* regions on chromosomes 3A and 4A, respectively. The tracing of pedigrees of AAC Tenacious and other sources complements the validation of QTL analysis results. Some of the PHS resistance QTLs have been cloned previously and a few of those, as discussed above, have also been physically located in the QTL intervals of the present study. By comparing our results with previous PHS studies in wheat, we have confirmed the position of many major PHS resistance QTLs and candidate genes. Despite the presence of such a great reservoir of important QTLs/genes in AAC Tenacious, many identified QTLs were detected in unique environments. This might be contributed by the high level of environmental effect, which requires the validation of environment-specific QTLs first before employing them in breeding programs.

## Method

### Plant material

A spring wheat recombinant doubled haploid mapping population (224 lines) was developed by crossing AAC Innova [76] as the female with AAC Tenacious as the male, followed by haploid induction using the wheat-maize pollination technique [95] at the Agriculture and Agri-Food Canada, Lethbridge Research and Development Centre (LeRDC), Lethbridge, AB, Canada. AAC Innova is a PHS susceptible, white-grained, semi-dwarf, soft white spring type cultivar which belongs to Canada Western Special Purpose market class. AAC Tenacious is a highly PHS resistant, red-grained, tall, hard red spring type cultivar which belongs to Canada Prairie Spring market class. AAC Innova originated from the cross AC Andrew/N9195 made at LeRDC in 2001 and developed using a modified bulk breeding technique [76]. AAC Tenacious was developed from the cross HY665/BW346 made at the Agriculture and Agri-Food Canada, Cereal Research Centre (CRC), Winnipeg, Manitoba during the winter of 2003–2004 [68].

A number of soft-white and hard-red spring wheat cvs belonging to different market classes and with varying levels of PHS resistance were used as checks for comparisons (Table 3).

**Table 3:** Details of check cultivars used for comparison of pre-harvest sprouting (PHS) resistance

Cultivar	Type	Pedigree	Origin	Reference
AAC Awesome	Soft white spring	93FHB37/2*Andrew//SWS366	AAFC-LeRDC	[96]
AAC Chiffon	Soft white spring	AC Reed/SWS53	AAFC-LeRDC	[97]
AAC Indus	Soft white spring	Sadash/SWS340	AAFC-LeRDC	[98]
AC Andrew	Soft white spring	Dirkwin/SC8021V2//Treasure/Blanca	AAFC-LeRDC	[99]
Sadash	Soft white spring	SWS207/SWS208//SWS214	AAFC-LeRDC	[100]
AAC Foray	Hard red spring	CPS03hnF4 5123.032/5701PR	AAFC-CRC	[101]
Cardale	Hard red spring	McKenzie/Alsen	AAFC-CRC	[102]
Conquer	Hard red spring	HY639/99 EPWA-Mdg61	AAFC-CRC	[103]
Enchant	Hard red spring	97-M-27/AC Vista	AAFC-CRC	[104]
Lillian	Hard red spring	BW621*3/90B07-AU2B	AAFC-CRC AAFC-SCRDC	[105]
Vesper	Hard red spring	Augusta/Hard White Alpha//3*AC Barrie/BW150*2//Tp/Tm/3/2*Superb/4/94B35-R5C/5/Superb	AAFC-CRC	[106]
AAC Brandon	Hard red spring	Superb/CDC Osler//ND744	AAFC-SCRDC	[107]
AAC Penhold	Hard red spring	700PR/HY644-BE//HY469	AAFC-SCRDC	[108]
Carberry	Hard red spring	Alsen/Superb	AAFC-SCRDC	[109]
Stettler	Hard red spring	Prodigy/Superb	AAFC-SCRDC	[110]
CDC Stanley	Hard red spring	W95132/AC Barrie	CDC-UofS	[111]

Note: *AAFC*- Agriculture and Agri-Food Canada, *CRC* Cereal Research Centre (CRC), Winnipeg, Manitoba, *LeRDC* Lethbridge Research and Development Centre, Lethbridge, Alberta, *SCRDC* Swift Current Research and Development Centre, Swift Current, Saskatchewan, *CDC* Crop Development Centre, University of Saskatchewan, Saskatoon, Saskatchewan

Seeds of cultivars used as checks and parents of the mapping population were accessed from Spring Wheat Breeding core collection at AAFC-LeRDC. DH lines produced in this study are preserved at AAFC-LeRDC and available upon request. All other cultivars used in this study are preserved at Plant Gene Resources of Canada (PGRC) seed genebank based at AAFC’s Saskatoon Research and Development Centre, Saskatoon, Saskatchewan, Canada.

### Trial environments and pre-harvest sprouting assessment

The recombinant doubled haploid lines, their parents and check cultivars were grown in field conditions in four environments: (i) University of Alberta, Edmonton, Canada in 2019 (EDM 2019), (ii) Cornell University, Ithaca, USA in 2018 (ITH 2018), (iii) LeRDC, Lethbridge, Canada in 2018 (LET 2018), and (iv) LeRDC, Lethbridge, Canada in 2019 (LET 2019). PHS resistance assessment at Cornell University, Ithaca, USA was carried out following Anderson et al. [112] and Munkvold et al. [72]. At the Edmonton and Lethbridge locations, PHS resistance assessment was carried out following Anderson et al. [112] and Paterson et al. [113] with some modifications. Mature heads of each genotype (recombinant DHs, parents and check cultivars) were harvested from the field trials at physiological maturity (+ 1 week), when most of the nodes collapsed in the plot. For each genotype, 15 heads (as 3 bundles, each of 5) were harvested. Harvested heads were spread out on benches in a greenhouse and left for 2 days at room temperature to dry. The dried heads were then stored at − 20 °C until assessments were undertaken.

For PHS resistance assessments, heads were removed from the − 20 °C cold room in the morning and kept at room temperature for 2 h followed by soaking in double-distilled water in plastic containers for another 2 h. After soaking, head bundles of DH lines along with their parents and checks were mounted upright on black plastic trays fixed on wire grid in a mist-chamber where they were moistened thoroughly from fixed spray nozzles. The mist-chamber was set at: 100% relative humidity, 25 °C and no light.

Sprouting was visually assessed on a daily basis in the mist chamber. When the sprouting distinguished both parents and the check cultivars by a maximum difference (when susceptible parent AAC Innova and check cultivars largely stopped sprouting new grains), head bundles were removed from the mist chamber and assessed for PHS. On average, the maximum difference was seen on 5th day. Thus, the wet head bundles were removed from the mist-chamber on the morning of day 5, and each bundle was assessed for the number of heads with different numbers of sprouts as follows:

*a* = # heads with 0 sprouts

*b* = # heads with 1 sprout

*c* = # heads with 2 sprouts

*d* = # heads with 3–5 sprouts

*e* = # heads with > 6–10 sprouts

*f* = # heads with 10+ sprouts

*g* = # total heads evaluated (5 in this case)

Using the numbers calculated above for bundles (reps), the bundle (rep) pre-harvest sprouting resistance (PHSR_n_) scores were calculated using weighted parameters given in DePauw et al. [114] as follows:

$$PHSRn=\frac{(a)1+(b)2+(c)3+(d)5+(e)7+(f)9}{g}$$

Genotype PHSR score was calculated by averaging individual bundle scores as follows:

$$PHSR=\frac{\left({PHSR}_1\right)+\left({PHSR}_2\right)+\left({PHSR}_3\right)}{3}$$

Using the above formula, the best PHS resistant line was rated as PHSR score 1 while the worst as PHSR score 9.

### Statistical analysis

All the statistical analyses were carried out using various software packages in R (version 3.2.3) [115], the software environment for statistical computing and graphics. For the ANOVA model, DHs, their parents and check cultivars were considered fixed effects, while environments were considered random effects. Mixed ANOVA and post-hoc tests, and visualization of results in graphical forms were carried out using R packages tidyverse (version 1.2.1) [116], ggpubr (version 0.4.0) [117] and rstatix (version 0.6.0) [118] following Kassambara [119]. Type-II analysis of variance of PHS data was calculated both within and across environments using the agricolae (version 1.2–4) package [120]. To counter the missing values, type-III analysis of variance was calculated using Satterthwaite’s method with the package ‘lmerTest’ [121]. Correlations and regression analyses among environments and scatterplots were calculated using the R package GGally [122].

### Quantitative trait loci analysis

QTL analysis was carried out using the previously developed AAC Innova/AAC Tenacious linkage map [75] from 188 DH lines and phenotypic data collected from four environments mentioned above following Dhariwal et al. [123]. Briefly, main effect QTLs were identified using the composite interval mapping (CIM) approach with the regression method forwards and backwards cofactor (*p* = 0.05) implemented in QTL Cartographer (version 1.6) [124, 125]. QTL thresholds were estimated using 1000 permutations at a significance level of *p* = 0.05. QTLs detected in at least one environment or the pooled data but had LOD score ≥ 2.5 were also reported. LOD peak spanning chromosome interval above the threshold/selected score were used to determine QTL intervals. LOD score thresholds were 3.1 at Edmonton 2019, 3.0 at Ithaca 2018, 3.4 at Lethbridge 2018, 3.4 at Lethbridge 2019, and 3.3 for the pooled data. Marker intervals that harboured two or more QTLs within 10.0 cM were considered as a single QTL region, while the rest of the QTL intervals (> 10.0 cM apart) were considered unique QTL regions [75]. Mixed-model based composite interval mapping (MCIM) to map main effect QTLs and two-locus QTL analysis to map epistatic QTLs were carried out using QTLNetwork (version 2.0) [126] following Dhariwal et al. [127]. QTL analyses results were represented as a Circos diagram using the R package OmicCircos (version 1.14.0) [128].

### Assignment of physical intervals and mapping of candidate genes

Wheat 90 K iSelect SNP assay probe sequences of all the SNP markers that mapped into QTL intervals in this study were BLAST searched (with at least 99% identity and 100% query coverage) against the wheat reference genome (IWGSC RefSeq v2.0) sequence using NCBI standalone BLAST program [129] to identify the physical interval on wheat chromosomes. Primer/probe sequences of flanking/linked markers of QTLs, identified previously from different genotypes during different studies as well as the gDNA/cDNA sequences of cloned PHS or dormancy related genes (Additional file 2: Table S9), were also BLAST searched against the wheat reference genome to identify common/shared QTL regions and candidate genes among different studies.

### Validation and assessment of the effect of *Ppd-D1* on PHS resistance

Associations of days to anthesis (DTA) and the two alleles (insensitive, *Ppd-D1a* and sensitive, *Ppd-D1b*) of domestication/photoperiodic response gene *Ppd-D1* with PHS resistance were assessed using correlation coefficient and boxplot analyses, respectively.

### Pedigree and genealogical analysis

Pedigrees of AAC Tenacious, AAC Innova, and other source cultivars were traced using the information from literature sources extracted from the ‘GRIS: Genetic Resources Information System for Wheat and Triticale’ database [130] (accessed: November 1–25, 2020), ‘UK wheat varieties pedigree’ dataset [131, 132], and publications by Martynov and Dobrotvorskaya [54], Osanai et al. [133] and Garlinge [134]. A color coded complete pedigree of AAC Tenacious was graphically generated using Helium Pedigree Visualization Framework [135].

## Supplementary Information

**Additional file 1: Figure S1.** Pre-harvest sprouting (PHS) phenotypes of check cultivars after 4 days in a mist chamber. Spike bundles from left to right of resistant (Cardale, Enchant, Stettler, AAC Tenacious, Penhold, CDC Stanley), intermediate (Vesper, AAC Indus, Carberry, Lillian, AAC Chiffon, AAC Brandon, AAC Foray) and susceptible (Sadash, AAC Innova, AAC Awesome, Conquer, AC Andrew) cultivars are shown in upper, middle and lower rows, respectively.

**Additional file 2: Tables S1 to S9.** Details of pre-harvest sprouting data, quantitative trait loci, associated markers, genetic/genomic positions, and previous studies.

**Additional file 3: Figure S2.** Effects of pre-harvest sprouting (PHS) resistance quantitative trait loci (QTLs) on sprouting. Effects of QTLs *QPhs.lrdc-1A.2*, *QPhs.lrdc-2B.1*, *QPhs.lrdc-3A.1*, *QPhs.lrdc-3B.2*, *QPhs.lrdc-3D.1*, *QPhs.lrdc-3D.2* and *QPhs.lrdc-7D*, respectively, shown as 1A.2, 2B.1, 3A.1, 3B.2, 3D.1, 3D.2 and 7D, are presented as bar plot and line graph.

**Additional file 4: Figure S3.** Complete pedigree of wheat cultivar AAC Tenacious. Different cultivars/landraces and their crosses in pedigree are shown in circles. Female and male parents are shown by pink and blue line connections. Circle colors dark green, light green, dark red, light red, dark amber and light amber represents pre-harvest sprouting (PHS) resistant progenitors of AAC Tenacious. Blue, light blue and gray circles represents other cultivars and crosses in the pedigree.

**Additional file 5.** Pedigree information of different wheat genotypes of different origin.

## Data Availability

Linkage map of AAC Innova/AAC Tenacious doubled haploid mapping population of spring wheat, which is used for the present study, has been published previously by authors (Dhariwal et al. [75]; 10.3390/ijms21124497). Linkage map is publically available as supplementary information of the published paper [75] and can either be downloaded from the link: https://www.mdpi.com/1422-0067/21/12/4497#supplementary or requested from the corresponding author. All other data generated or analysed during this study are included in this published article and its supplementary information files.

## Abbreviations

AA: Additive × additive effect or epistasis interaction effect
AAC: Prefix to cultivar names Agriculture Canada (Agriculture and Agri-Food Canada)
AAFC: Agriculture and Agri-Food Canada
ABA: Abscisic acid
ABI3: Abscisic acid insensitive3
AC: Prefix to cultivar names Agriculture Canada (Agriculture and Agri-Food Canada)
AGO: Argonaute
ANOVA: Analysis of variance
BLAST: Basic local alignment search tool
bZIP: Basic leucine zipper domain
CDC: Crop Development Centre
cDNA: Complementary deoxyribonucleic acid
CHI: Chalcone isomerase
CHS: Chalcone synthase
CIM: Composite interval mapping
cM: Centimorgan, a linkage map unit
CPS: Canada Prairie Spring wheat class
CRC: Cereal Research Centre, Winnipeg, Manitoba
CRISPR/Cas9: Clustered regularly interspaced short palindromic repeats and CRISPR-associated protein 9
CWSP: Canada Western Special Purpose wheat class
cv: Cultivar
cvs: Cultivars
CYP: Cytochromes P450 superfamily of enzymes
DH: Doubled haploid
DNA: Deoxyribonucleic acid
*DOG1*: Delay of germination 1
Dor: Dormancy
EDM: Edmonton
Fn: Falling number
*FT*: Flowering locus T
F3H: Flavanone 3-hydroxylase
GA: Gibberellic acid or gibberellin
GC: Grain color/colour
gDNA: Genomic deoxyribonucleic acid
GI: Germination index
*HUB1*: Histone monoubiquitination1
ITH: Ithaca
IWGSC: International Wheat Genome Sequencing Consortium
LET: Lethbridge
LG: Linkage group
LOD: Logarithm of the odds
LeRDC: Lethbridge Research and Development Centre, AB, Canada
lrdc: Lethbridge Research and Development Centre, AB, Canada
MCIM: Mixed-model based composite interval mapping
*MFT*: Mother of FT and TFL1
MKK: Mitogen-activated protein kinase kinase
MR: Moderately resistant
MS: Moderately susceptible
*Myb*: Myeloblastosis family of transcription factor gene
NCBI: National Center for Biotechnology Information
PEBP: Plant phosphatidylethanolamine binding protein family
PHS: Pre-harvest sprouting
PHSR: Pre-harvest sprouting resistance
PHT: Plant height
PM: Plasma membrane
*Ppd*: Photoperiod responsive
PV: Phenotypic variation
PVE: Phenotypic variation explained
*QPhs*: Quantitative trait loci for pre-harvest sprouting
QTL: Quantitative trait loci
R: Resistant/Resistant gene/Resistance
*r*: Correlation coefficient
*R*^*2*^: Phenotypic variation
RdDM: RNA dependant DNA methylation pathway
RDO: Reduced dormancy
RefSeq: Reference Sequence
*Rht*: Reduced height gene
RSP: Name of a Chinese wheat landrace
S: Susceptible
SCRDC: Swift Current Research and Development Centre, Swift Current, Saskatchewan
SD: Seed dormancy
*Sdr*: Seed dormancy gene
SNP: Single nucleotide polymorphism
SSR: Simple sequence repeat
*Ta*: *Triticum aestivum*
*TFL1*: Terminal Flower1
UofS: University of Saskatchewan, Saskatoon, Saskatchewan
USA: The United States of America
USD: United States Dollar
v: Version
*Vp*: Viviparous
