# Supplementary figures and images for: Mapping pre-harvest sprouting resistance loci in AAC Innova × AAC Tenacious spring wheat population
Source: BMC Genomics. 2021 Dec 15;22:900. doi: 10.1186/s12864-021-08209-6 (PMC8675488; doi:10.1186/s12864-021-08209-6)

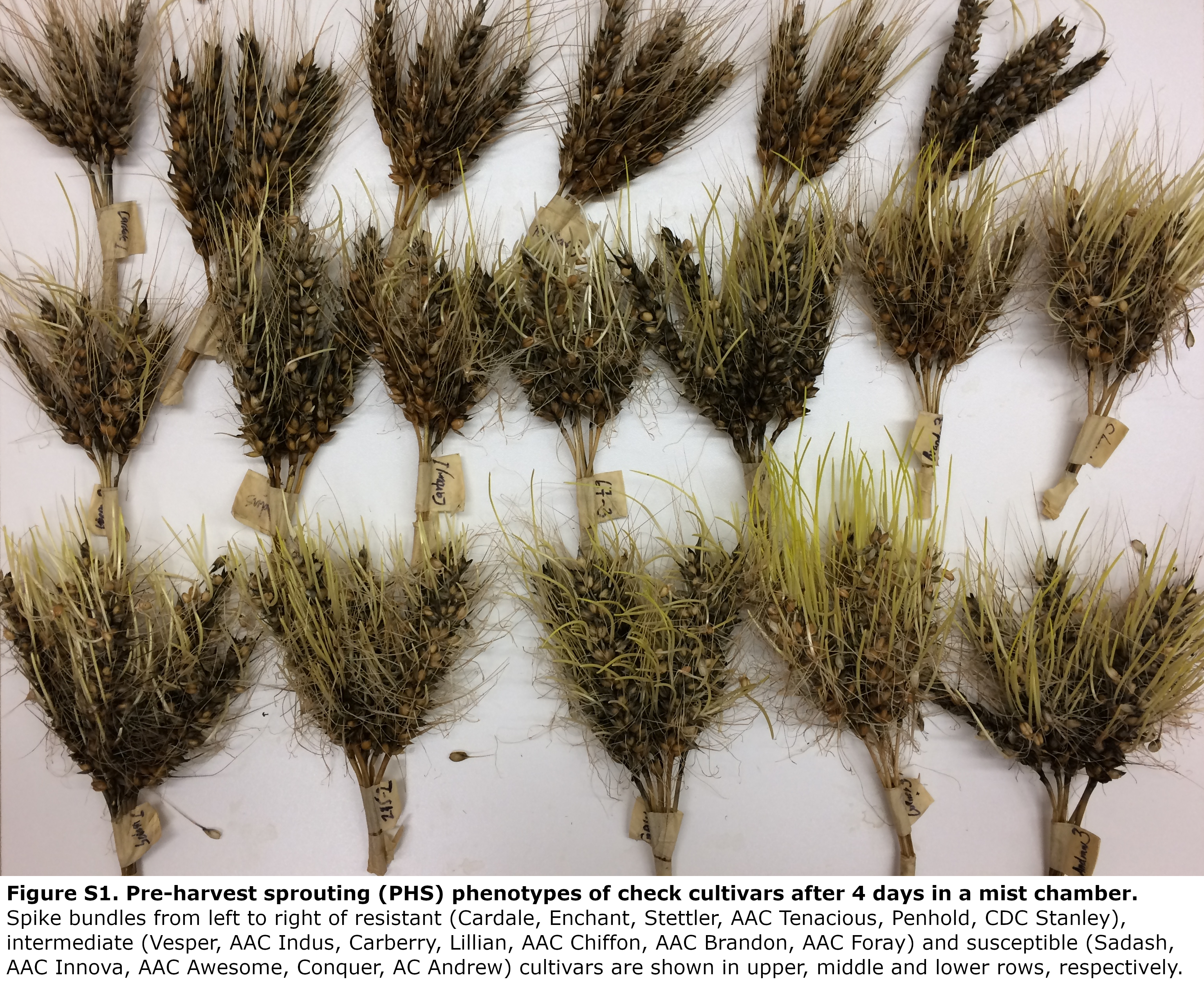

Supplement: Supplementary file 1 — Additional file 1: Figure S1. Pre-harvest sprouting (PHS) phenotypes of check cultivars after 4 days in a mist chamber. Spike bundles from left to right of resistant (Cardale, Enchant, Stettler, AAC Tenacious, Penhold, CDC Stanley), intermediate (Vesper, AAC Indus, Carberry, Lillian, AAC Chiffon, AAC Brandon, AAC Foray) and susceptible (Sadash, AAC Innova, AAC Awesome, Conquer, AC Andrew) cultivars are shown in upper, middle and lower rows, respectively. [file 12864_2021_8209_MOESM1_ESM.jpg]

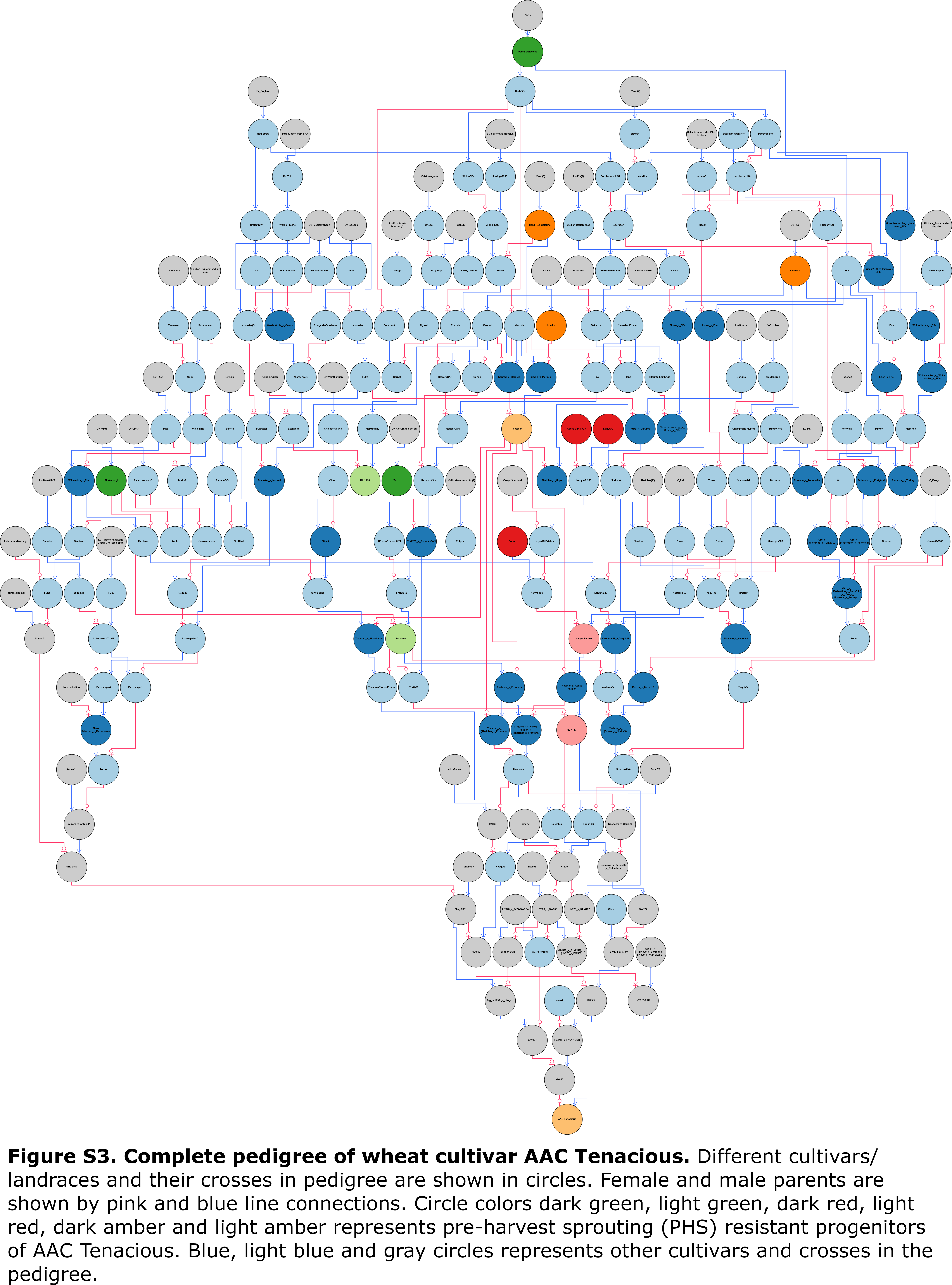

Supplement: Supplementary file 4 — Additional file 4: Figure S3. Complete pedigree of wheat cultivar AAC Tenacious. Different cultivars/landraces and their crosses in pedigree are shown in circles. Female and male parents are shown by pink and blue line connections. Circle colors dark green, light green, dark red, light red, dark amber and light amber represents pre-harvest sprouting (PHS) resistant progenitors of AAC Tenacious. Blue, light blue and gray circles represents other cultivars and crosses in the pedigree. [file 12864_2021_8209_MOESM4_ESM.tif]
