# Supplementary material for: Mapping pre-harvest sprouting resistance loci in AAC Innova × AAC Tenacious spring wheat population
Source: BMC Genomics. 2021 Dec 15;22:900. doi: 10.1186/s12864-021-08209-6 (PMC8675488; doi:10.1186/s12864-021-08209-6)
Supplement: Supplementary file 3 — Additional file 3: Figure S2. Effects of pre-harvest sprouting (PHS) resistance quantitative trait loci (QTLs) on sprouting. Effects of QTLs QPhs.lrdc-1A.2, QPhs.lrdc-2B.1, QPhs.lrdc-3A.1, QPhs.lrdc-3B.2, QPhs.lrdc-3D.1, QPhs.lrdc-3D.2 and QPhs.lrdc-7D, respectively, shown as 1A.2, 2B.1, 3A.1, 3B.2, 3D.1, 3D.2 and 7D, are presented as bar plot and line graph. [file 12864_2021_8209_MOESM3_ESM.pdf]

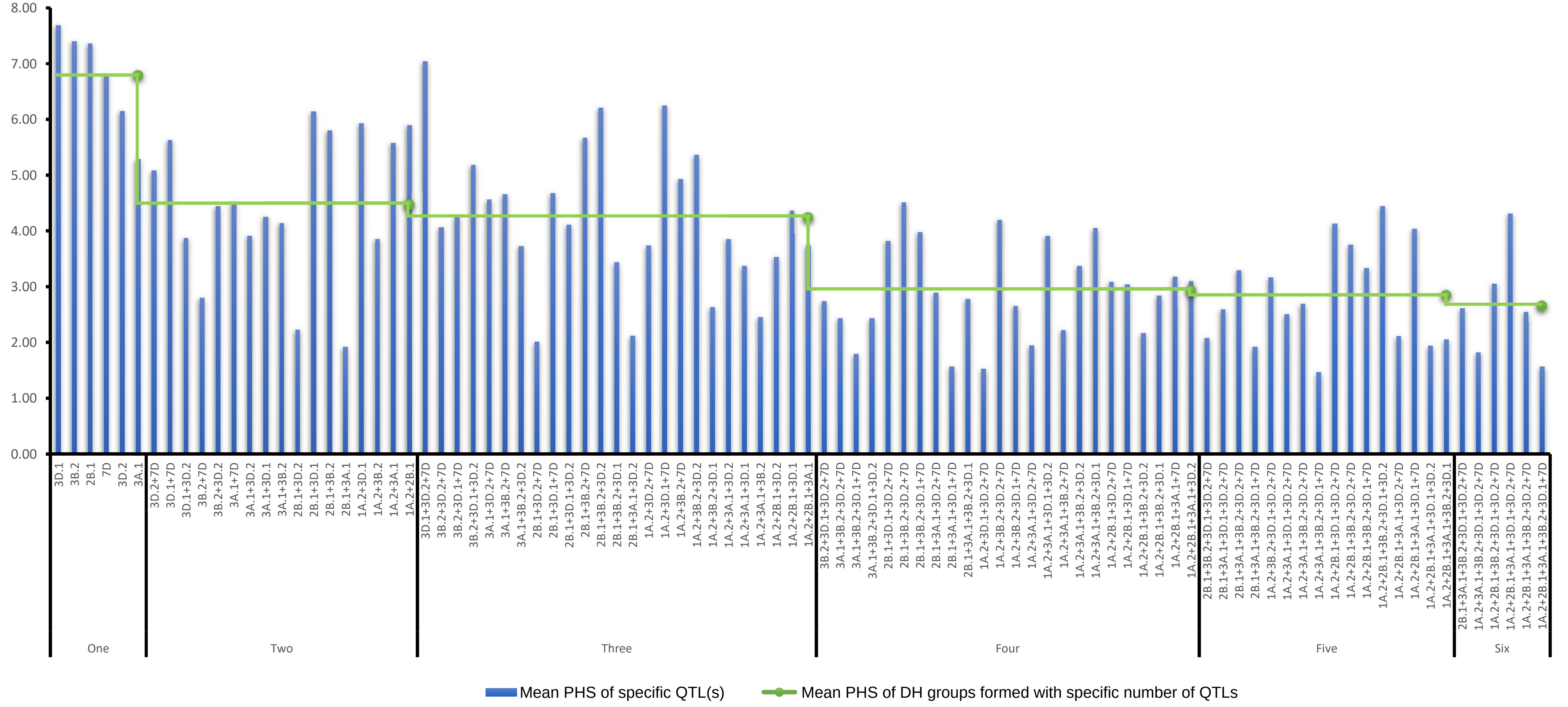

Figure S2. Effects of pre-harvest sprouting (PHS) resistance quantitative trait loci (QTLs) on sprouting. Effects of QTLs *QPhs.lrdc-1A.2, QPhs.lrdc-2B.1, QPhs.lrdc-3A.1, QPhs.lrdc-3B.2, QPhs.lrdc-3D.1, QPhs.lrdc-3D.2* and *QPhs.lrdc-7D*, respectively, shown as 1A.2, 2B.1, 3A.1, 3B.2, 3D.1, 3D.2 and 7D, are presented as bar plot and line graph.
