# Supplementary material for: Mapping pre-harvest sprouting resistance loci in AAC Innova × AAC Tenacious spring wheat population
Source: BMC Genomics. 2021 Dec 15;22:900. doi: 10.1186/s12864-021-08209-6 (PMC8675488; doi:10.1186/s12864-021-08209-6)
Supplement: Supplementary file 5 — Additional file 5. Pedigree information of different wheat genotypes of different origin. [file 12864_2021_8209_MOESM5_ESM.pdf]

Pedigrees of different wheat genotypes of different origin.

Downloaded from: wheatpedigree.net/

Osanai et al. 2015. **Euphytica (2005) 143: 301-307.**

Garlinge, J. (2005), **2005 Crop variety sowing guide for Western Australia. Department of Agriculture and Food, Western Australia, Perth. Bulletin 4655.**

on: 11/19/2020

- AC-DOMAIN
  - ND-585
    - RL-4205
          - RED-EGYPTIAN
          - 6 * MARQUIS
        - 2 * THATCHER
        - 6 * PEMBINA
          - SELKIRK
          - THATCHER
          - TRANSFER
          - 3 * THATCHER
        - 6 * PEMBINA
          - SELKIRK
          - THATCHER
    - WALDRON
      - ND-81
        - ND-34
            - MIDA,USA
            - LEE
          - KENYA-FARMER
        - LEE
          - TIMSTEIN
          - HOPE
      - JUSTIN
        - ND-40-2
          - NS-3880-227
          - ND-4
        - CONLEY
          - LEE
          - RL-2563
    - 3 * BUTTE
      - WISCONSIN-261
          - 4 * WISCONSIN-250-6-10-2-2-1
            - WISCONSIN-244
            - HENRY
          - KENYA-184
            - RELIANCE
            - KENYA-73
            - 7 * WISCONSIN-250-2-3-3-1
            - KENYA-184
              - P-54
              - 7 * HENRY
            - PENJAMO-T-62
        - POLK
            - FRONTANA
              - NEWTHATCH
              - KENYA-58
            - SURPRESA
            - THATCHER
        - ND-480
          - ND-264
              - II-143-2C-2C-1C
              - LEE
            - ND-138
          - JUSTIN
            - ND-40-2
            - CONLEY
  - RL-4471
    - RL-4137
        - KENYA-FARMER
          - KENYA-192
            - KENYA-73-D-2-I-1-L
            - BUTTON
          - AUSTRALIA-27
            - 2 * BOBIN
            - GAZA
        - 6 * THATCHER
            - KANRED
            - MARQUIS
            - IUMILLO
            - MARQUIS
      - RL-2520
          - 2 * REDMAN
          - RL-2265
            - EXCHANGE
            - MCMURACHY
        - FRONTANA
          - MENTANA
            - AKAKOMUGI
              - WILHELMINA
              - RIETI
          - FRONTEIRA
            - ALFREDO-CHAVES-6-21
            - POLYSSU
    - ND-499
      - WALDRON
        - ND-81
          - ND-34
              - MIDA,USA
              - LEE
            - KENYA-FARMER
          - LEE
            - TIMSTEIN
            - HOPE
        - JUSTIN
          - ND-40-2
            - NS-3880-227
            - ND-4
          - CONLEY
            - LEE
            - RL-2563
          - JUSTIN
            - ND-40-2
            - CONLEY
              - ND-122
              - CONLEY
              - CONLEY
              - ND-259

- 2 * JUSTIN
  - ND-40-2
    - NS-3880-227
    - ND-4
  - CONLEY
    - LEE
    - RL-2563

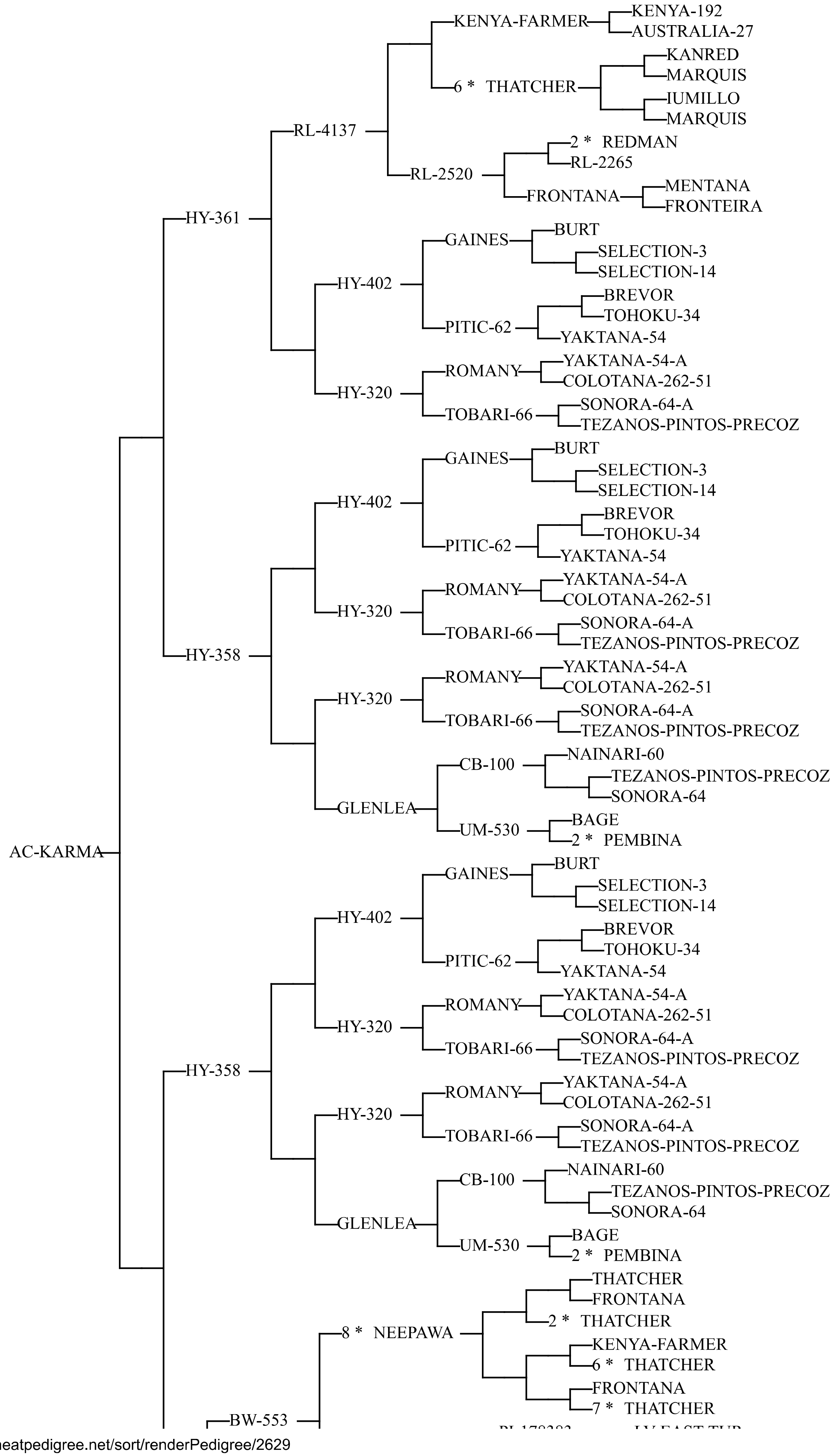
AC-KARMA
HY-361
RL-4137
KENYA-FARMER
KENYA-192
AUSTRALIA-27
6 * THATCHER
KANRED
MARQUIS
IUMILLO
MARQUIS
RL-2520
2 * REDMAN
RL-2265
FRONTANA
MENTANA
FRONTEIRA
HY-402
GAINES
BURT
SELECTION-3
SELECTION-14
PITIC-62
BREVOR
TOHOKU-34
YAKTANA-54
HY-320
ROMANY
YAKTANA-54-A
COLOTANA-262-51
TOBARI-66
SONORA-64-A
TEZANOS-PINTOS-PRECOZ
HY-358
HY-402
GAINES
BURT
SELECTION-3
SELECTION-14
PITIC-62
BREVOR
TOHOKU-34
YAKTANA-54
HY-320
ROMANY
YAKTANA-54-A
COLOTANA-262-51
TOBARI-66
SONORA-64-A
TEZANOS-PINTOS-PRECOZ
HY-320
ROMANY
YAKTANA-54-A
COLOTANA-262-51
TOBARI-66
SONORA-64-A
TEZANOS-PINTOS-PRECOZ
GLENLEA
CB-100
NAINARI-60
TEZANOS-PINTOS-PRECOZ
SONORA-64
UM-530
BAGE
2 * PEMBINA
HY-358
HY-402
GAINES
BURT
SELECTION-3
SELECTION-14
PITIC-62
BREVOR
TOHOKU-34
YAKTANA-54
HY-320
ROMANY
YAKTANA-54-A
COLOTANA-262-51
TOBARI-66
SONORA-64-A
TEZANOS-PINTOS-PRECOZ
HY-320
ROMANY
YAKTANA-54-A
COLOTANA-262-51
TOBARI-66
SONORA-64-A
TEZANOS-PINTOS-PRECOZ
GLENLEA
CB-100
NAINARI-60
TEZANOS-PINTOS-PRECOZ
SONORA-64
UM-530
BAGE
2 * PEMBINA
BW-553
8 * NEEPAWA
THATCHER
FRONTANA
2 * THATCHER
KENYA-FARMER
6 * THATCHER
FRONTANA
7 * THATCHER

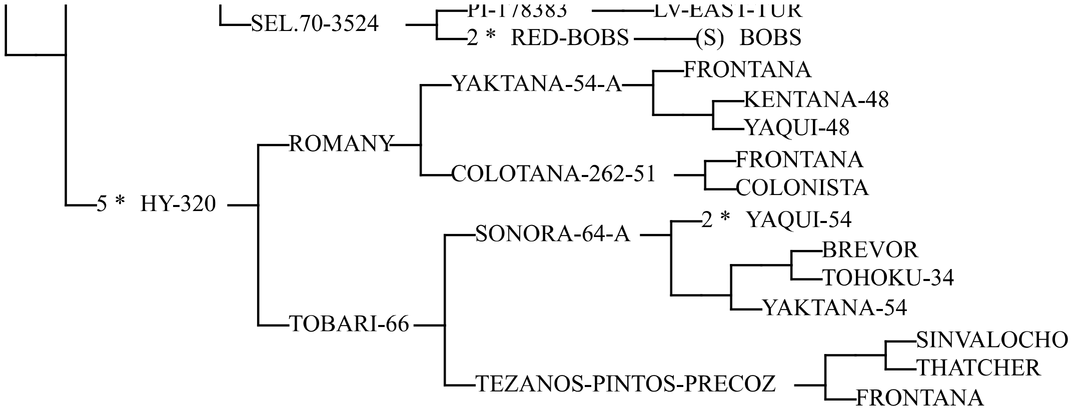
SEL.70-3524
PI-178383
LV-EAST-TUR
2 * RED-BOBS
(S) BOBS
5 * HY-320
ROMANY
YAKTANA-54-A
FRONTANA
KENTANA-48
YAQUI-48
COLOTANA-262-51
FRONTANA
COLONISTA
TOBARI-66
SONORA-64-A
2 * YAQUI-54
BREVOR
TOHOKU-34
YAKTANA-54
TEZANOS-PINTOS-PRECOZ
SINVALOCHO
THATCHER
FRONTANA

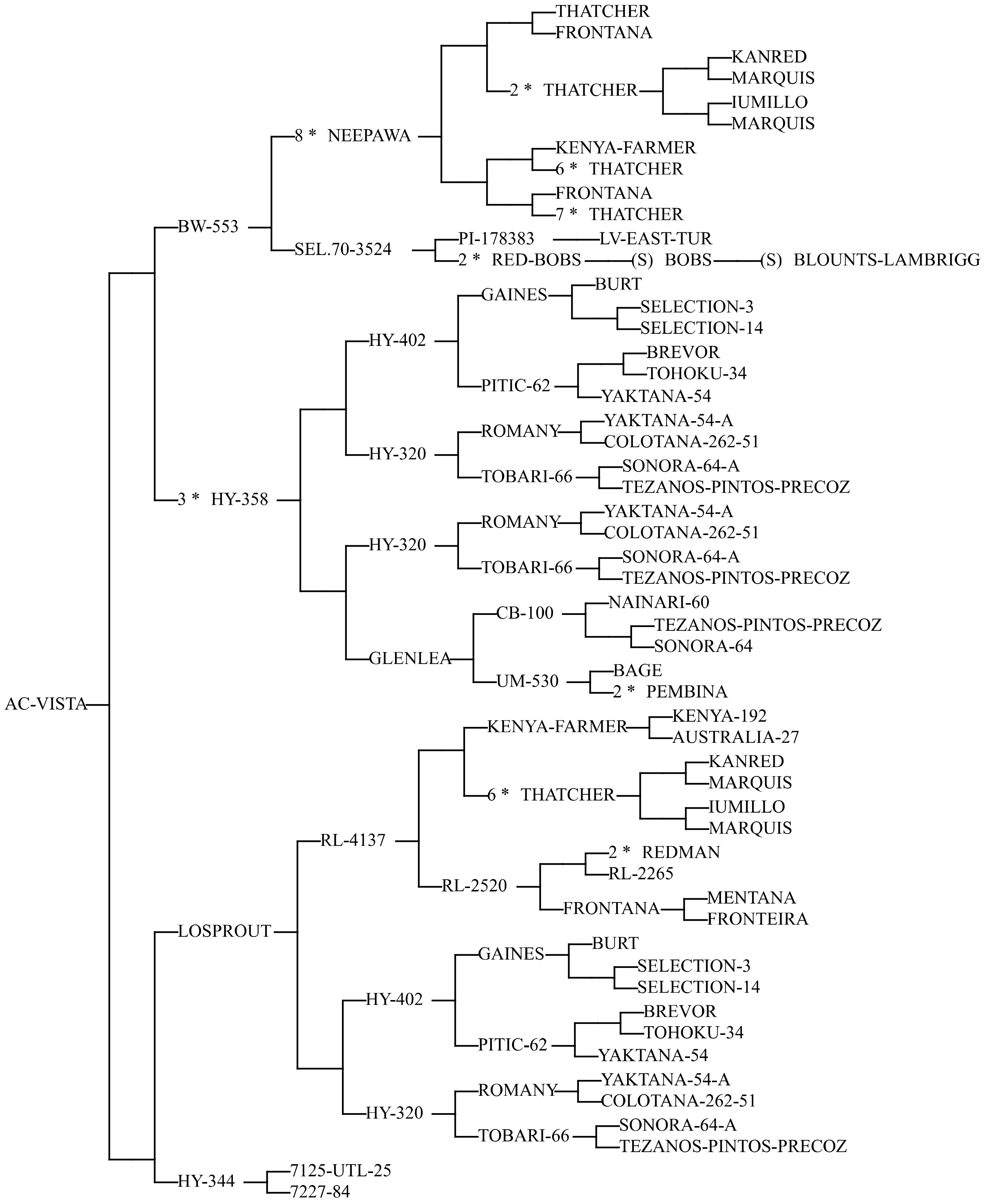
THATCHER
FRONTANA
2 * THATCHER
KANRED
MARQUIS
IUMILLO
MARQUIS
8 * NEEPAWA
KENYA-FARMER
6 * THATCHER
FRONTANA
7 * THATCHER
BW-553
SEL.70-3524
PI-178383
LV-EAST-TUR
2 * RED-BOBS
(S) BOBS
(S) BLOUNTS-LAMBRIGG
GAINES
BURT
SELECTION-3
SELECTION-14
HY-402
BREVOR
TOHOKU-34
PITIC-62
YAKTANA-54
ROMANY
YAKTANA-54-A
COLOTANA-262-51
HY-320
TOBARI-66
SONORA-64-A
TEZANOS-PINTOS-PRECOZ
3 * HY-358
ROMANY
YAKTANA-54-A
COLOTANA-262-51
HY-320
TOBARI-66
SONORA-64-A
TEZANOS-PINTOS-PRECOZ
CB-100
NAINARI-60
TEZANOS-PINTOS-PRECOZ
SONORA-64
GLENLEA
UM-530
BAGE
2 * PEMBINA
AC-VISTA
KENYA-FARMER
KENYA-192
AUSTRALIA-27
6 * THATCHER
KANRED
MARQUIS
IUMILLO
MARQUIS
RL-4137
2 * REDMAN
RL-2265
RL-2520
FRONTANA
MENTANA
FRONTEIRA
LOSPROUT
GAINES
BURT
SELECTION-3
SELECTION-14
HY-402
BREVOR
TOHOKU-34
PITIC-62
YAKTANA-54
ROMANY
YAKTANA-54-A
COLOTANA-262-51
HY-320
TOBARI-66
SONORA-64-A
TEZANOS-PINTOS-PRECOZ
HY-344
7125-UTL-25
7227-84

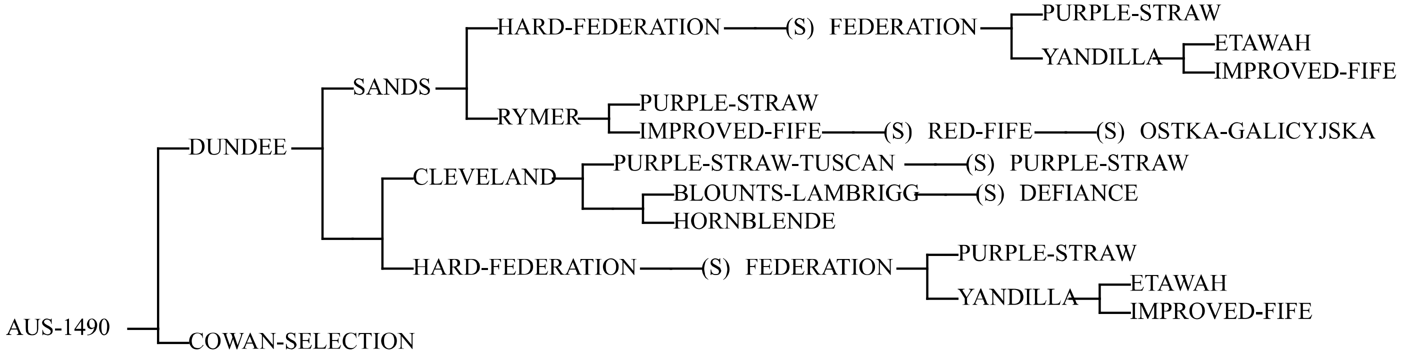
AUS-1490
DUNDEE
COWAN-SELECTION
SANDS
HARD-FEDERATION
(S) FEDERATION
PURPLE-STRAW
YANDILLA
ETAWAH
IMPROVED-FIFE
RYMER
PURPLE-STRAW
IMPROVED-FIFE
(S) RED-FIFE
(S) OSTKA-GALICYJSKA
CLEVELAND
PURPLE-STRAW-TUSCAN
(S) PURPLE-STRAW
BLOUNTS-LAMBRIGG
(S) DEFIANCE
HORNBLENDE
HARD-FEDERATION
(S) FEDERATION
PURPLE-STRAW
YANDILLA
ETAWAH
IMPROVED-FIFE

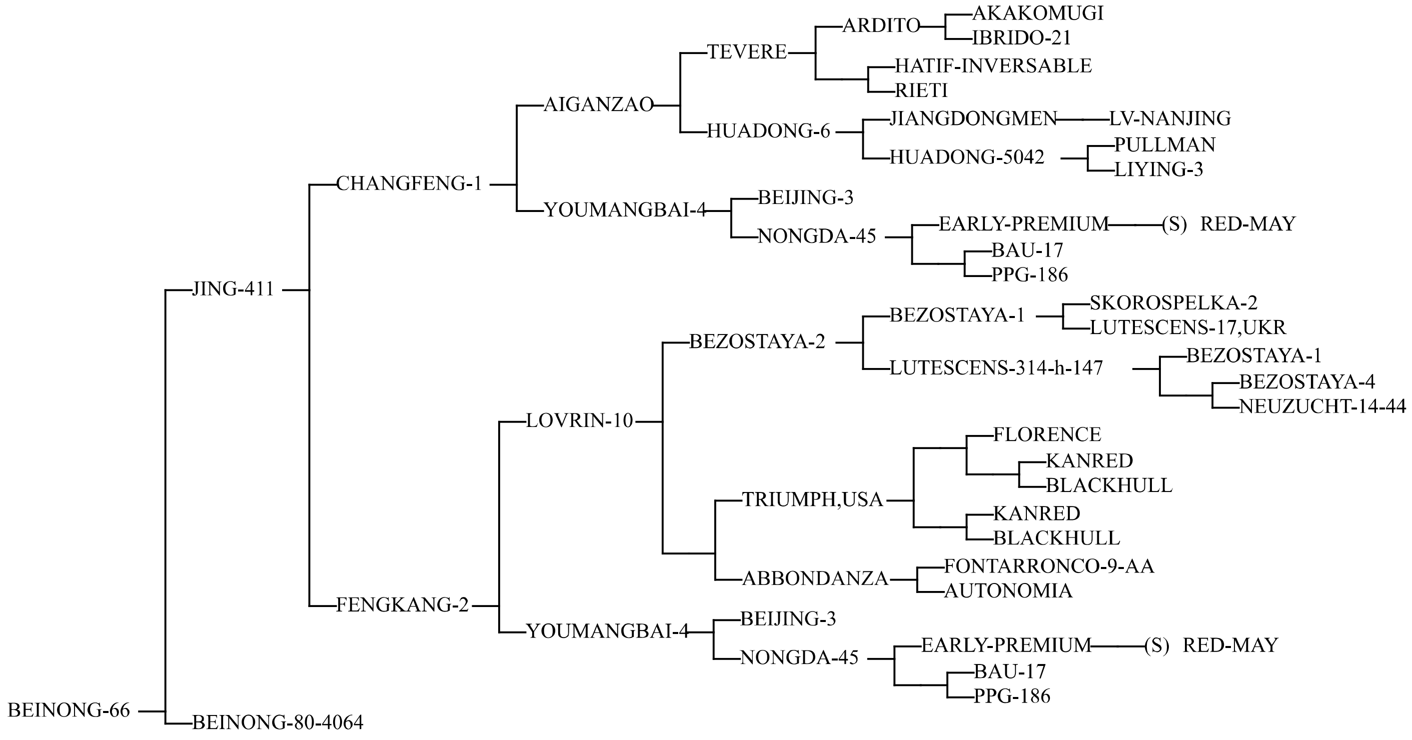
BEINONG-66
JING-411
BEINONG-80-4064
CHANGFENG-1
FENGKANG-2
AIGANZAO
YOUMANGBAI-4
TEVERE
HUADONG-6
ARDITO
AKAKOMUGI
IBRIDO-21
HATIF-INVERSABLE
RIETI
JIANGDONGMEN
LV-NANJING
HUADONG-5042
PULLMAN
LIYING-3
BEIJING-3
NONGDA-45
EARLY-PREMIUM
(S) RED-MAY
BAU-17
PPG-186
LOVRIN-10
BEZOSTAYA-2
BEZOSTAYA-1
SKOROSPELKA-2
LUTESCENS-17,UKR
LUTESCENS-314-h-147
BEZOSTAYA-1
BEZOSTAYA-4
NEUZUCHT-14-44
TRIUMPH,USA
FLORENCE
KANRED
BLACKHULL
KANRED
BLACKHULL
ABBONDANZA
FONTARRONCO-9-AA
AUTONOMIA
YOUMANGBAI-4
BEIJING-3
NONGDA-45
EARLY-PREMIUM
(S) RED-MAY
BAU-17
PPG-186

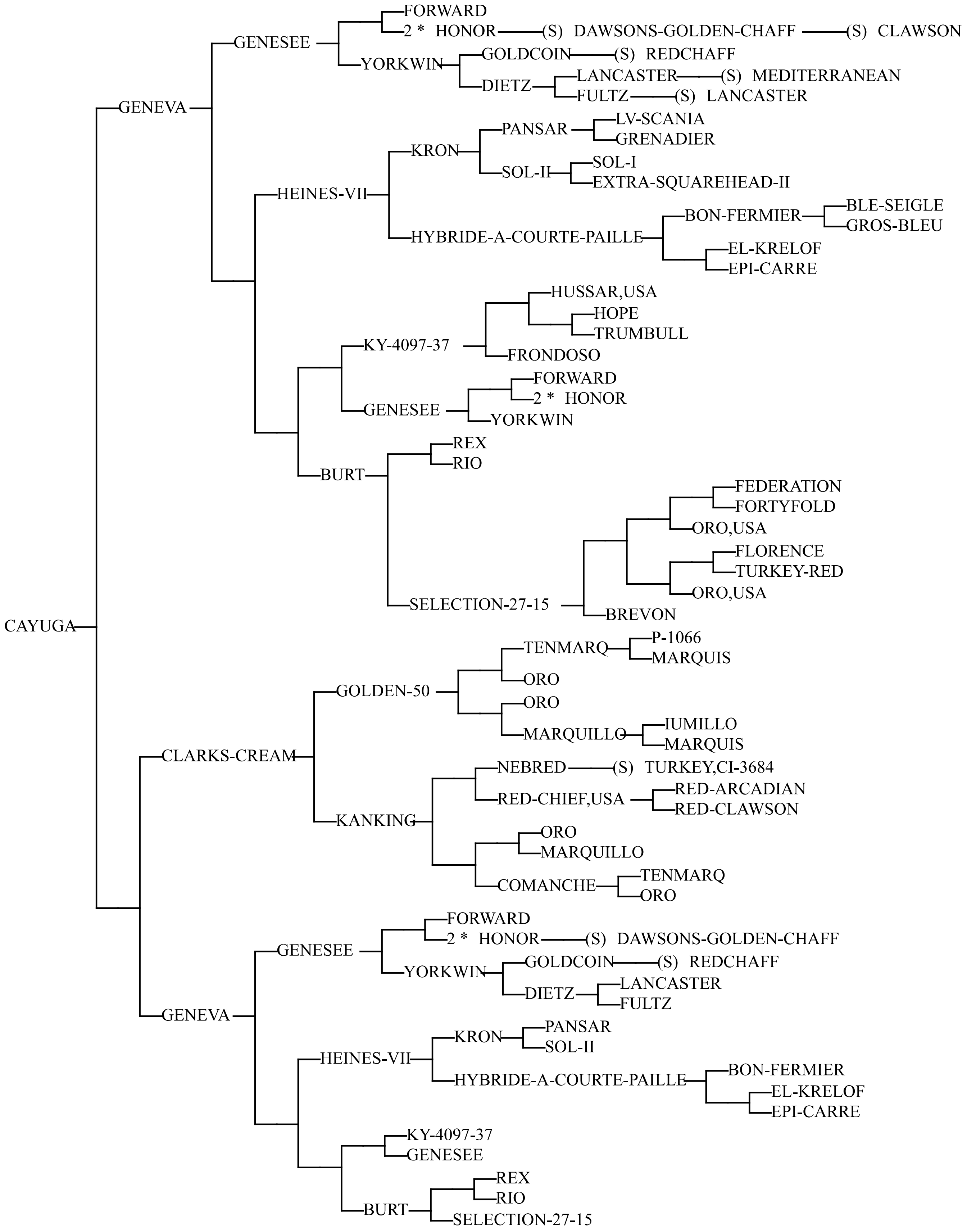
FORWARD
2 * HONOR (S) DAWSONS-GOLDEN-CHAFF (S) CLAWSON
GENESEE
GOLDCOIN (S) REDCHAFF
YORKWIN
LANCASTER (S) MEDITERRANEAN
DIETZ
FULTZ (S) LANCASTER
GENEVA
PANSAR
LV-SCANIA
GRENADIER
KRON
SOL-II
SOL-I
EXTRA-SQUAREHEAD-II
HEINES-VII
BON-FERMIER
BLE-SEIGLE
GROS-BLEU
HYBRIDE-A-COURTE-PAILLE
EL-KRELOF
EPI-CARRE
HUSSAR,USA
HOPE
TRUMBULL
KY-4097-37
FRONDOSO
FORWARD
2 * HONOR
GENESEE
YORKWIN
REX
RIO
BURT
FEDERATION
FORTYFOLD
ORO,USA
FLORENCE
TURKEY-RED
ORO,USA
SELECTION-27-15
BREVON
CAYUGA
P-1066
TENMARQ
MARQUIS
ORO
GOLDEN-50
ORO
IUMILLO
MARQUILLO
MARQUIS
CLARKS-CREAM
NEBRED (S) TURKEY,CI-3684
RED-ARCADIAN
RED-CHIEF,USA
RED-CLAWSON
KANKING
ORO
MARQUILLO
TENMARQ
COMANCHE
ORO
FORWARD
2 * HONOR (S) DAWSONS-GOLDEN-CHAFF
GENESEE
GOLDCOIN (S) REDCHAFF
YORKWIN
LANCASTER
DIETZ
FULTZ
GENEVA
PANSAR
KRON
SOL-II
HEINES-VII
BON-FERMIER
HYBRIDE-A-COURTE-PAILLE
EL-KRELOF
EPI-CARRE
KY-4097-37
GENESEE
REX
RIO
BURT
SELECTION-27-15

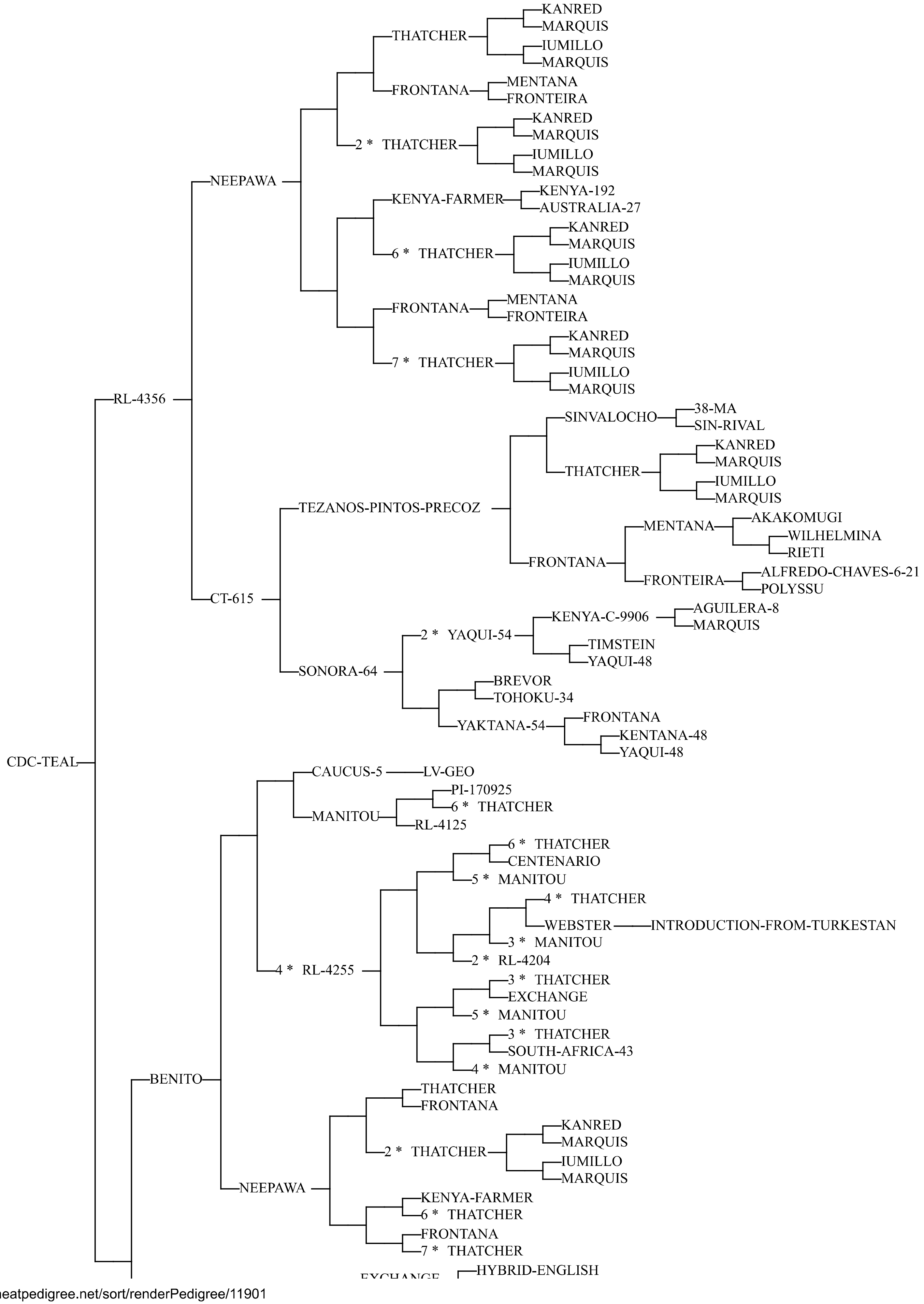
KANRED
THATCHER
MARQUIS
IUMILLO
MARQUIS
MENTANA
FRONTANA
FRONTEIRA
KANRED
MARQUIS
2 * THATCHER
IUMILLO
MARQUIS
NEEPAWA
KENYA-192
KENYA-FARMER
AUSTRALIA-27
KANRED
MARQUIS
6 * THATCHER
IUMILLO
MARQUIS
MENTANA
FRONTANA
FRONTEIRA
KANRED
MARQUIS
7 * THATCHER
IUMILLO
MARQUIS
RL-4356
38-MA
SINVALOCHO
SIN-RIVAL
KANRED
MARQUIS
THATCHER
IUMILLO
MARQUIS
TEZANOS-PINTOS-PRECOZ
AKAKOMUGI
MENTANA
WILHELMINA
RIETI
FRONTANA
ALFREDO-CHAVES-6-21
FRONTEIRA
POLYSSU
CT-615
AGUILERA-8
KENYA-C-9906
MARQUIS
2 * YAQUI-54
TIMSTEIN
YAQUI-48
SONORA-64
BREVOR
TOHOKU-34
FRONTANA
YAKTANA-54
KENTANA-48
YAQUI-48
CDC-TEAL
CAUCUS-5
LV-GEO
PI-170925
6 * THATCHER
MANITOU
RL-4125
6 * THATCHER
CENTENARIO
5 * MANITOU
4 * THATCHER
WEBSTER
INTRODUCTION-FROM-TURKESTAN
3 * MANITOU
4 * RL-4255
2 * RL-4204
3 * THATCHER
EXCHANGE
5 * MANITOU
3 * THATCHER
SOUTH-AFRICA-43
4 * MANITOU
BENITO
THATCHER
FRONTANA
KANRED
MARQUIS
2 * THATCHER
IUMILLO
MARQUIS
NEEPAWA
KENYA-FARMER
6 * THATCHER
FRONTANA
7 * THATCHER
HYBRID-ENGLISH

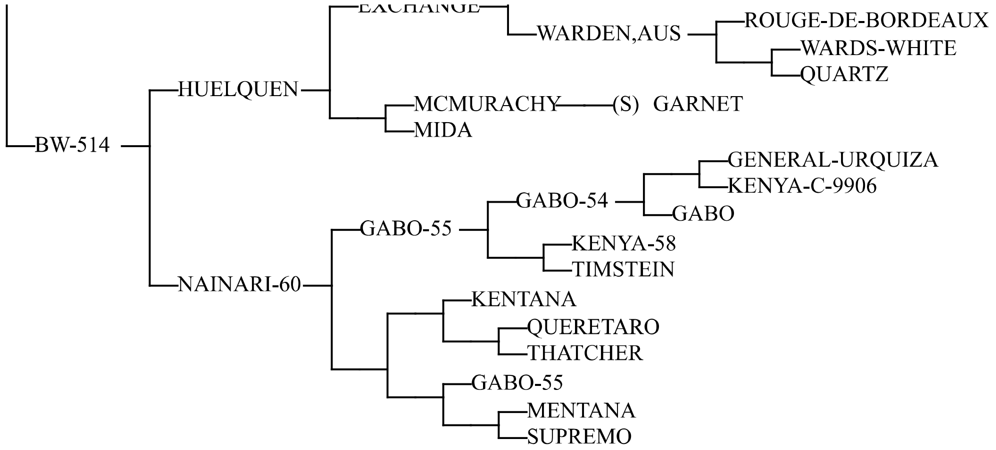
WARDEN,AUS
ROUGE-DE-BORDEAUX
WARDS-WHITE
QUARTZ
HUELQUEN
MCMURACHY
(S) GARNET
MIDA
BW-514
GENERAL-URQUIZA
KENYA-C-9906
GABO-54
GABO
GABO-55
KENYA-58
TIMSTEIN
NAINARI-60
KENTANA
QUERETARO
THATCHER
GABO-55
MENTANA
SUPREMO

CHINESE-SPRING ———LV-WEST-SZECHUAN

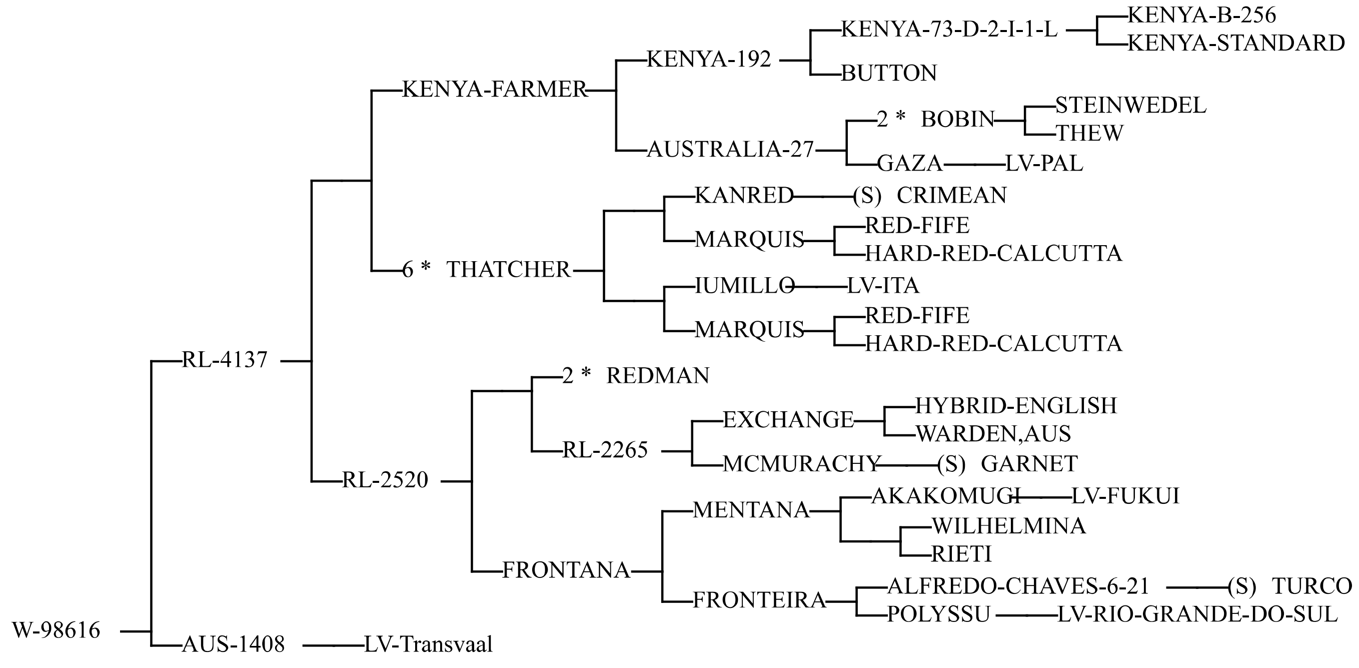
KENYA-73-D-2-I-1-L
KENYA-B-256
KENYA-STANDARD
KENYA-192
BUTTON
KENYA-FARMER
2 * BOBIN
STEINWEDEL
THEW
AUSTRALIA-27
GAZA
LV-PAL
KANRED
(S) CRIMEAN
MARQUIS
RED-FIFE
HARD-RED-CALCUTTA
6 * THATCHER
IUMILLO
LV-ITA
MARQUIS
RED-FIFE
HARD-RED-CALCUTTA
RL-4137
2 * REDMAN
EXCHANGE
HYBRID-ENGLISH
WARDEN,AUS
RL-2265
MCMURACHY
(S) GARNET
RL-2520
MENTANA
AKAKOMUGI
LV-FUKUI
WILHELMINA
RIETI
FRONTANA
FRONTEIRA
ALFREDO-CHAVES-6-21
(S) TURCO
POLYSSU
LV-RIO-GRANDE-DO-SUL
W-98616
AUS-1408
LV-Transvaal

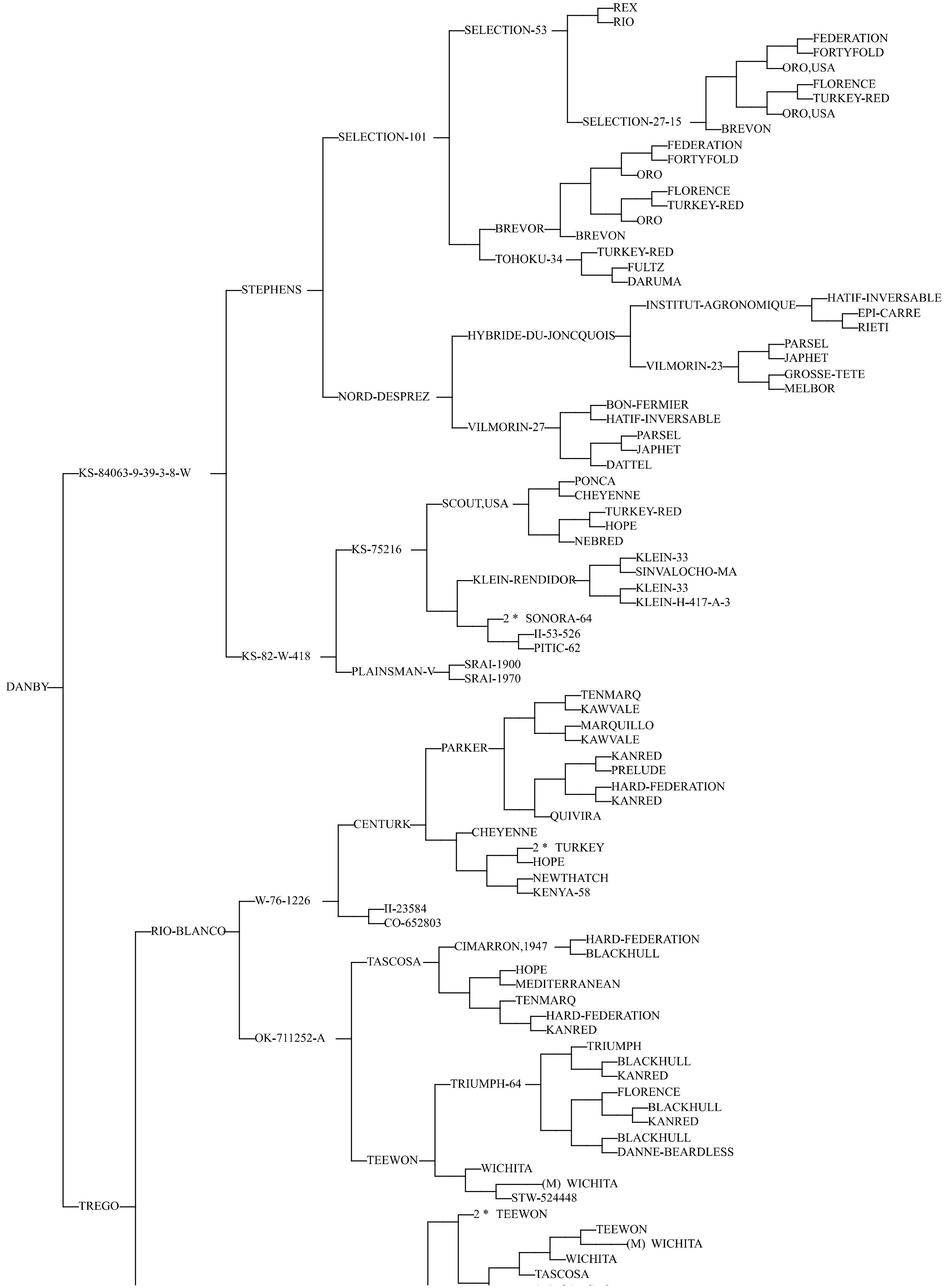
DANBY
KS-84063-9-39-3-8-W
STEPHENS
SELECTION-101
SELECTION-53
REX
RIO
SELECTION-27-15
FEDERATION
FORTYFOLD
ORO,USA
FLORENCE
TURKEY-RED
ORO,USA
BREVON
BREVOR
FEDERATION
FORTYFOLD
ORO
FLORENCE
TURKEY-RED
ORO
BREVON
TOHOKU-34
TURKEY-RED
FULTZ
DARUMA
NORD-DESPREZ
HYBRIDE-DU-JONCQUOIS
INSTITUT-AGRONOMIQUE
HATIF-INVERSABLE
EPI-CARRE
RIETI
VILMORIN-23
PARSEL
JAPHET
GROSSE-TETE
MELBOR
VILMORIN-27
BON-FERMIER
HATIF-INVERSABLE
PARSEL
JAPHET
DATTEL
KS-82-W-418
KS-75216
SCOUT,USA
PONCA
CHEYENNE
TURKEY-RED
HOPE
NEBRED
KLEIN-RENDIDOR
KLEIN-33
SINVALOCHO-MA
KLEIN-33
KLEIN-H-417-A-3
2 * SONORA-64
II-53-526
PITIC-62
PLAINSMAN-V
SRAI-1900
SRAI-1970
TREGO
RIO-BLANCO
W-76-1226
CENTURK
PARKER
TENMARQ
KAWVALE
MARQUILLO
KAWVALE
KANRED
PRELUDE
HARD-FEDERATION
KANRED
QUIVIRA
CHEYENNE
2 * TURKEY
HOPE
NEWTHATCH
KENYA-58
II-23584
CO-652803
OK-711252-A
TASCOSA
CIMARRON,1947
HARD-FEDERATION
BLACKHULL
HOPE
MEDITERRANEAN
TENMARQ
HARD-FEDERATION
KANRED
TEEWON
TRIUMPH-64
TRIUMPH
BLACKHULL
KANRED
FLORENCE
BLACKHULL
KANRED
BLACKHULL
DANNE-BEARDLESS
WICHITA
(M) WICHITA
STW-524448
2 * TEEWON
TEEWON
(M) WICHITA
WICHITA
TASCOSA

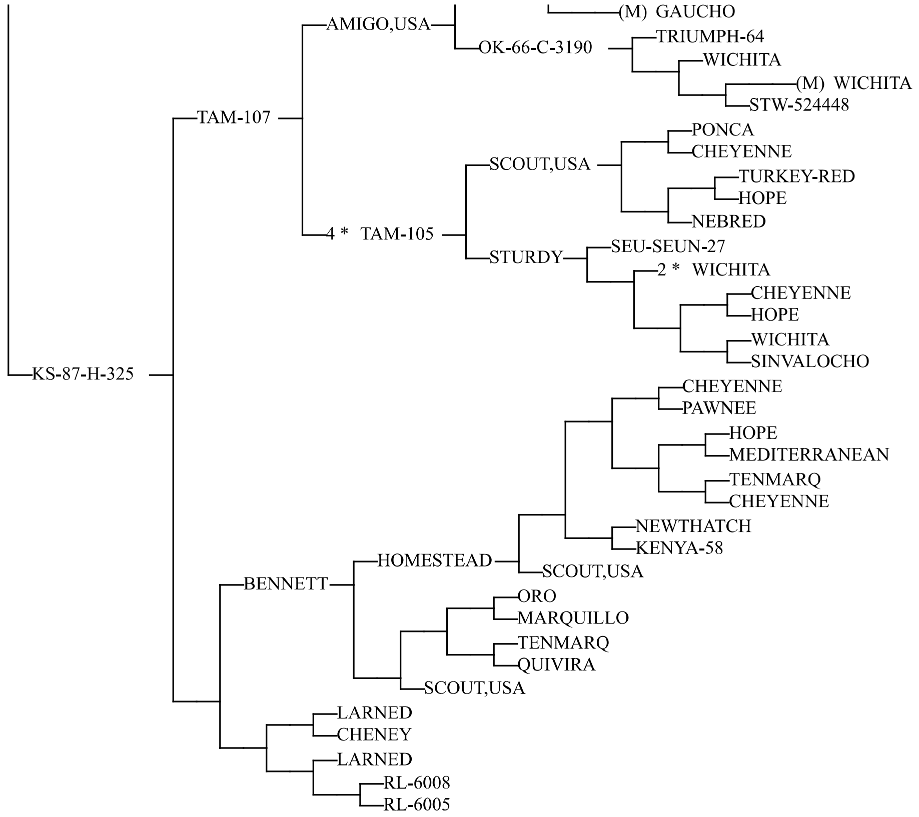
(M) GAUCHO
AMIGO,USA
OK-66-C-3190
TRIUMPH-64
WICHITA
(M) WICHITA
STW-524448
TAM-107
SCOUT,USA
PONCA
CHEYENNE
TURKEY-RED
HOPE
NEBRED
4 * TAM-105
SEU-SEUN-27
STURDY
2 * WICHITA
CHEYENNE
HOPE
WICHITA
SINVALOCHO
KS-87-H-325
CHEYENNE
PAWNEE
HOPE
MEDITERRANEAN
TENMARQ
CHEYENNE
NEWTHATCH
KENYA-58
HOMESTEAD
SCOUT,USA
BENNETT
ORO
MARQUILLO
TENMARQ
QUIVIRA
SCOUT,USA
LARNED
CHENEY
LARNED
RL-6008
RL-6005

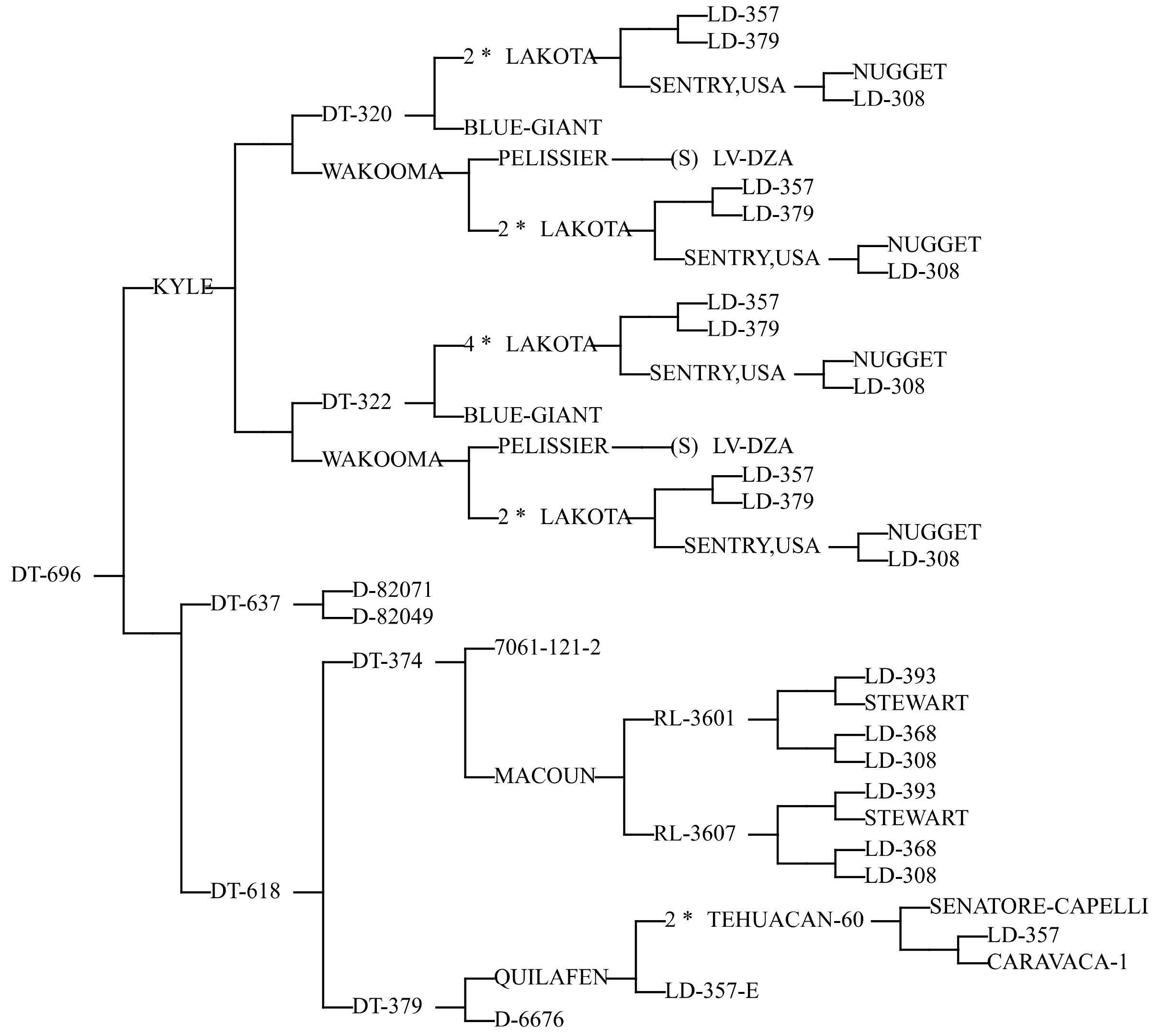
LD-357
LD-379
2 * LAKOTA
SENTRY,USA
NUGGET
LD-308
DT-320
BLUE-GIANT
WAKOOMA
PELISSIER
(S) LV-DZA
LD-357
LD-379
2 * LAKOTA
SENTRY,USA
NUGGET
LD-308
KYLE
LD-357
LD-379
4 * LAKOTA
SENTRY,USA
NUGGET
LD-308
DT-322
BLUE-GIANT
WAKOOMA
PELISSIER
(S) LV-DZA
LD-357
LD-379
2 * LAKOTA
SENTRY,USA
NUGGET
LD-308
DT-696
D-82071
DT-637
D-82049
DT-374
7061-121-2
LD-393
STEWART
RL-3601
LD-368
LD-308
MACOUN
LD-393
STEWART
RL-3607
LD-368
LD-308
DT-618
SENATORE-CAPELLI
2 * TEHUACAN-60
LD-357
CARAVACA-1
QUILAFEN
LD-357-E
DT-379
D-6676

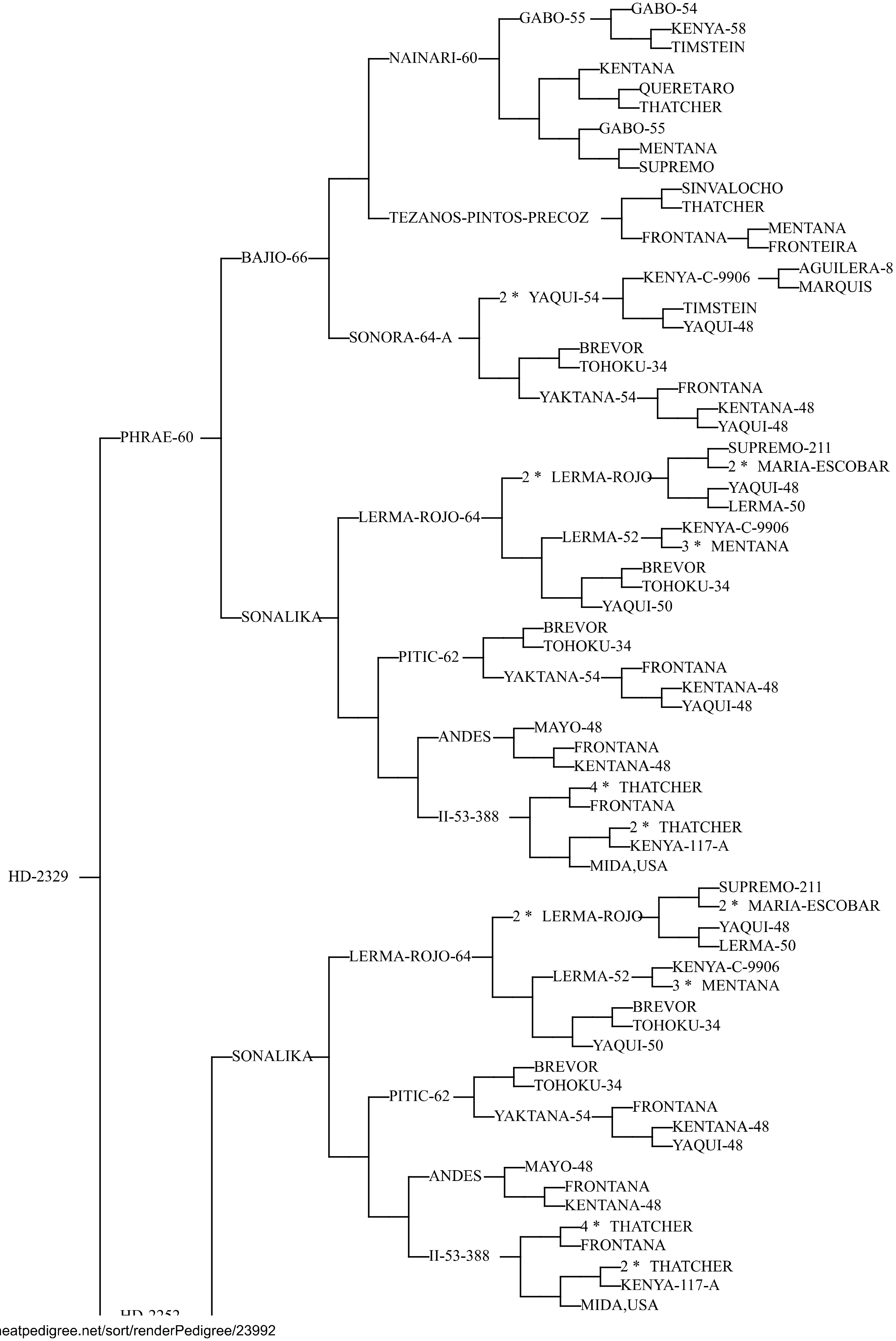
GABO-55
GABO-54
KENYA-58
TIMSTEIN
NAINARI-60
KENTANA
QUERETARO
THATCHER
GABO-55
MENTANA
SUPREMO
SINVALOCHO
THATCHER
TEZANOS-PINTOS-PRECOZ
FRONTANA
MENTANA
FRONTEIRA
BAJIO-66
KENYA-C-9906
AGUILERA-8
MARQUIS
2 * YAQUI-54
TIMSTEIN
YAQUI-48
SONORA-64-A
BREVOR
TOHOKU-34
FRONTANA
YAKTANA-54
KENTANA-48
YAQUI-48
PHRAE-60
SUPREMO-211
2 * MARIA-ESCOBAR
2 * LERMA-ROJO
YAQUI-48
LERMA-50
LERMA-ROJO-64
KENYA-C-9906
LERMA-52
3 * MENTANA
BREVOR
TOHOKU-34
YAQUI-50
SONALIKA
BREVOR
PITIC-62
TOHOKU-34
FRONTANA
YAKTANA-54
KENTANA-48
YAQUI-48
MAYO-48
ANDES
FRONTANA
KENTANA-48
4 * THATCHER
FRONTANA
II-53-388
2 * THATCHER
KENYA-117-A
MIDA,USA
HD-2329
SUPREMO-211
2 * MARIA-ESCOBAR
2 * LERMA-ROJO
YAQUI-48
LERMA-50
LERMA-ROJO-64
KENYA-C-9906
LERMA-52
3 * MENTANA
BREVOR
TOHOKU-34
YAQUI-50
SONALIKA
BREVOR
TOHOKU-34
PITIC-62
FRONTANA
YAKTANA-54
KENTANA-48
YAQUI-48
MAYO-48
ANDES
FRONTANA
KENTANA-48
4 * THATCHER
FRONTANA
II-53-388
2 * THATCHER
KENYA-117-A
MIDA,USA

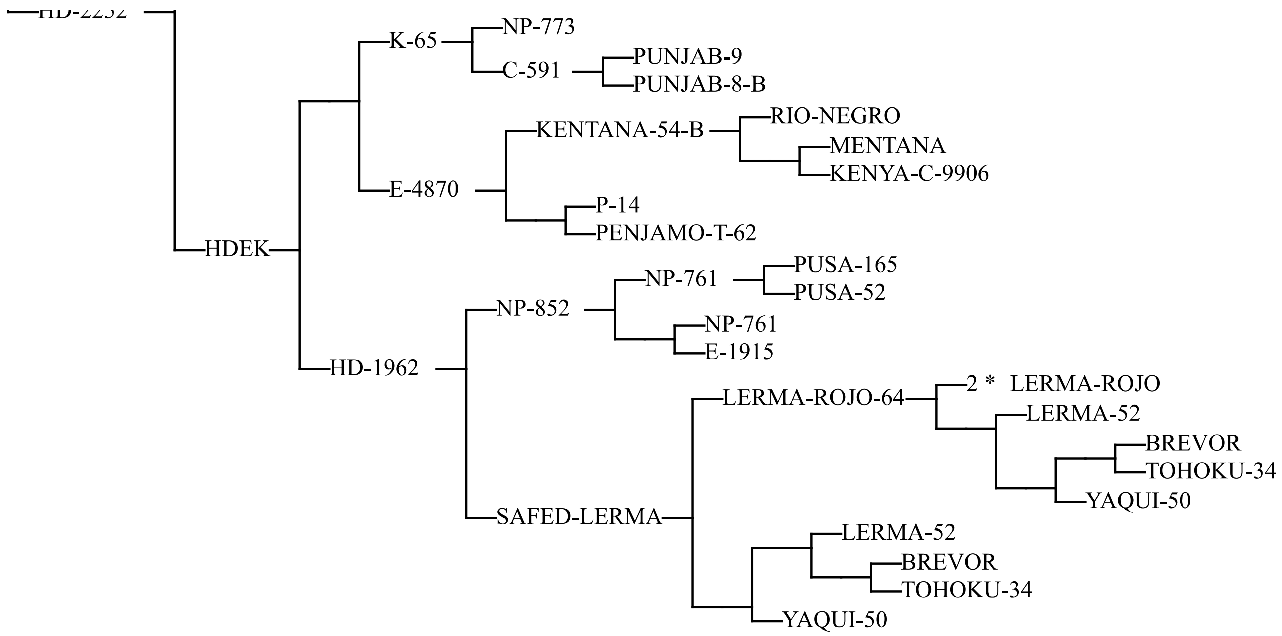
HDEK
K-65
NP-773
C-591
PUNJAB-9
PUNJAB-8-B
E-4870
KENTANA-54-B
RIO-NEGRO
MENTANA
KENYA-C-9906
P-14
PENJAMO-T-62
HD-1962
NP-852
NP-761
PUSA-165
PUSA-52
NP-761
E-1915
SAFED-LERMA
LERMA-ROJO-64
2 * LERMA-ROJO
LERMA-52
BREVOR
TOHOKU-34
YAQUI-50
LERMA-52
BREVOR
TOHOKU-34
YAQUI-50

HOPE
- MARQUIS
  - RED-FIFE — (S) OSTKA-GALICYJSKA — LV-POL
  - HARD-RED-CALCUTTA — LV-IND
- YAROSLAV-EMMER — LV-RUS,YAROSLAVL

KENYA-C-9906
- AGUILERA-8 — (S) AGUILERA — LV-MEX
- MARQUIS
  - RED-FIFE — (S) OSTKA-GALICYJSKA — LV-POL
  - HARD-RED-CALCUTTA — LV-IND

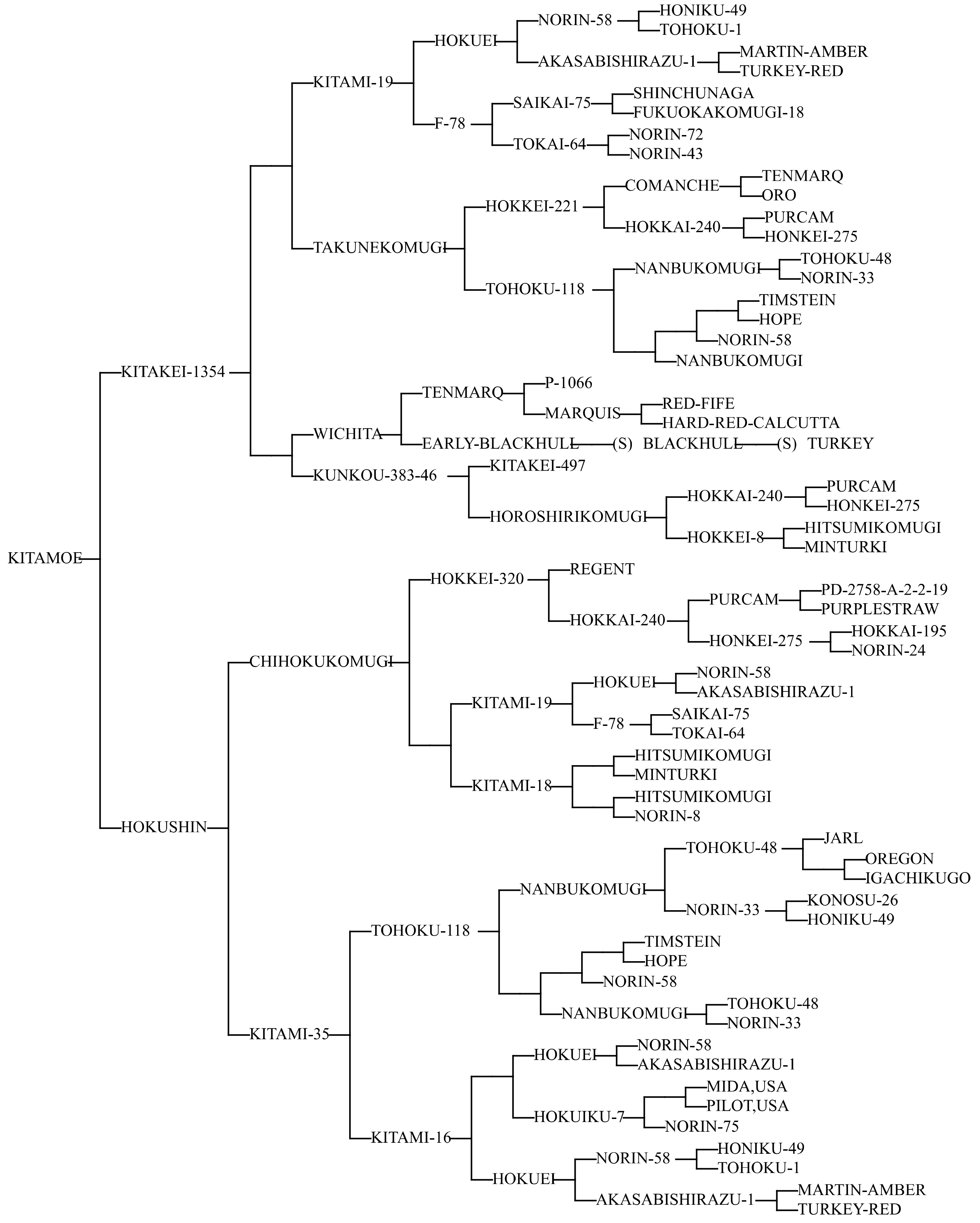
KITAMOE
KITAKEI-1354
KITAMI-19
HOKUEI
NORIN-58
HONIKU-49
TOHOKU-1
AKASABISHIRAZU-1
MARTIN-AMBER
TURKEY-RED
F-78
SAIKAI-75
SHINCHUNAGA
FUKUOKAKOMUGI-18
TOKAI-64
NORIN-72
NORIN-43
TAKUNEKOMUGI
HOKKEI-221
COMANCHE
TENMARQ
ORO
HOKKAI-240
PURCAM
HONKEI-275
TOHOKU-118
NANBUKOMUGI
TOHOKU-48
NORIN-33
TIMSTEIN
HOPE
NORIN-58
NANBUKOMUGI
WICHITA
TENMARQ
P-1066
MARQUIS
RED-FIFE
HARD-RED-CALCUTTA
EARLY-BLACKHULL
(S) BLACKHULL
(S) TURKEY
KUNKOU-383-46
KITAKEI-497
HOROSHIRIKOMUGI
HOKKAI-240
PURCAM
HONKEI-275
HOKKEI-8
HITSUMIKOMUGI
MINTURKI
HOKUSHIN
CHIHOKUKOMUGI
HOKKEI-320
REGENT
HOKKAI-240
PURCAM
PD-2758-A-2-2-19
PURPLESTRAW
HONKEI-275
HOKKAI-195
NORIN-24
KITAMI-19
HOKUEI
NORIN-58
AKASABISHIRAZU-1
F-78
SAIKAI-75
TOKAI-64
KITAMI-18
HITSUMIKOMUGI
MINTURKI
HITSUMIKOMUGI
NORIN-8
KITAMI-35
TOHOKU-118
NANBUKOMUGI
TOHOKU-48
JARL
OREGON
IGACHIKUGO
NORIN-33
KONOSU-26
HONIKU-49
TIMSTEIN
HOPE
NORIN-58
NANBUKOMUGI
TOHOKU-48
NORIN-33
KITAMI-16
HOKUEI
NORIN-58
AKASABISHIRAZU-1
HOKUIKU-7
MIDA,USA
PILOT,USA
NORIN-75
HOKUEI
NORIN-58
HONIKU-49
TOHOKU-1
AKASABISHIRAZU-1
MARTIN-AMBER
TURKEY-RED

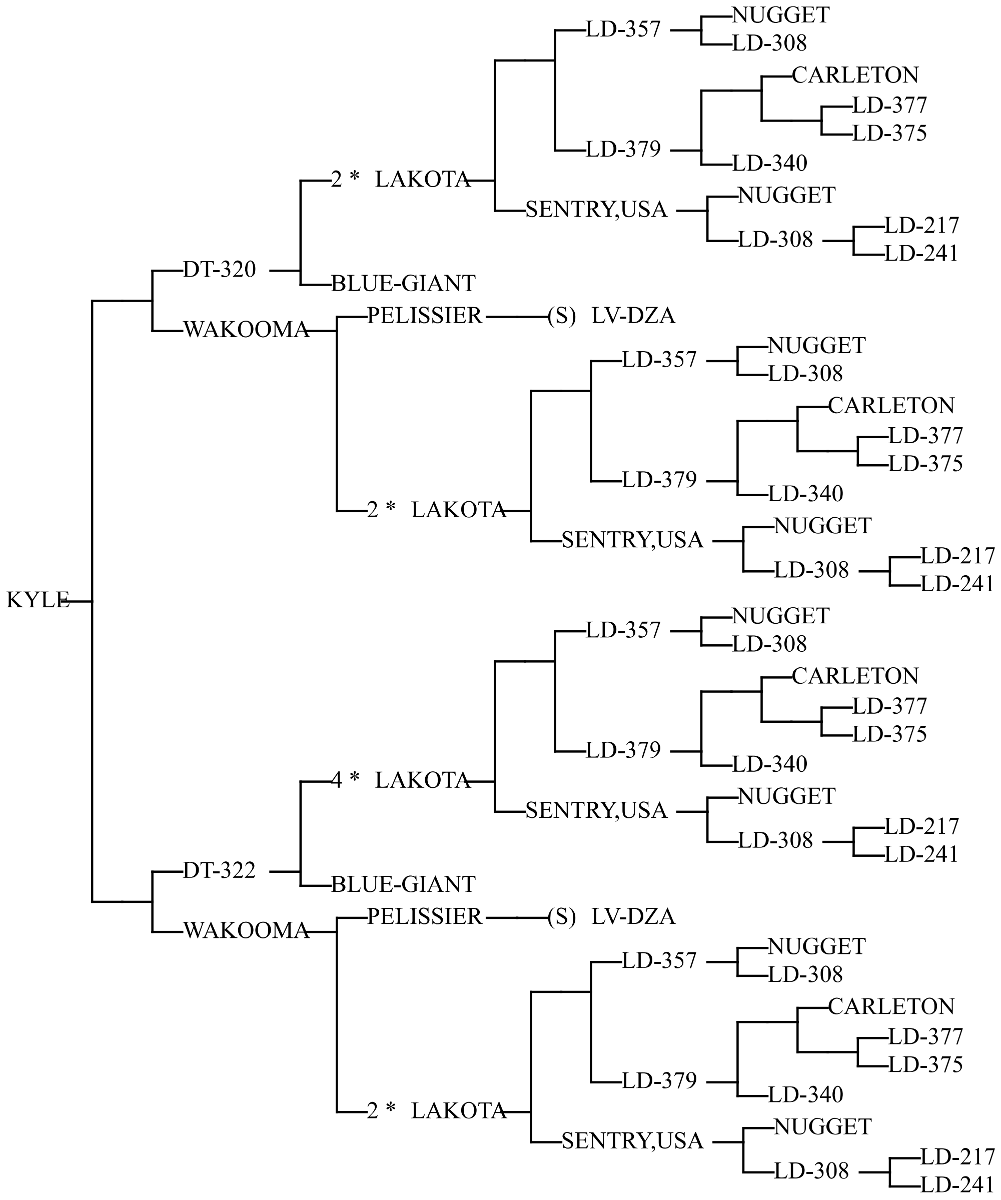
KYLE
DT-320
2 * LAKOTA
LD-357
NUGGET
LD-308
LD-379
CARLETON
LD-377
LD-375
LD-340
SENTRY,USA
NUGGET
LD-308
LD-217
LD-241
BLUE-GIANT
WAKOOMA
PELISSIER
(S) LV-DZA
2 * LAKOTA
LD-357
NUGGET
LD-308
LD-379
CARLETON
LD-377
LD-375
LD-340
SENTRY,USA
NUGGET
LD-308
LD-217
LD-241
DT-322
4 * LAKOTA
LD-357
NUGGET
LD-308
LD-379
CARLETON
LD-377
LD-375
LD-340
SENTRY,USA
NUGGET
LD-308
LD-217
LD-241
BLUE-GIANT
WAKOOMA
PELISSIER
(S) LV-DZA
2 * LAKOTA
LD-357
NUGGET
LD-308
LD-379
CARLETON
LD-377
LD-375
LD-340
SENTRY,USA
NUGGET
LD-308
LD-217
LD-241

- LEADER
  - CHRIS
    - 2 * THATCHER
      - KANRED —— (S) CRIMEAN
      - MARQUIS
        - RED-FIFE
        - HARD-RED-CALCUTTA
      - IUMILLO —— LV-ITA
      - MARQUIS
        - RED-FIFE
        - HARD-RED-CALCUTTA
    - NEWTHATCH
      - 2 * THATCHER
        - KANRED
        - MARQUIS
        - IUMILLO
        - MARQUIS
      - THATCHER
      - HOPE
    - KENYA-58
      - KENYA-BF-4-3-B-10-V-1
      - RED-EGYPTIAN —— LV-EGY
    - 3 * THATCHER
      - KANRED —— (S) CRIMEAN
      - MARQUIS
        - RED-FIFE
        - HARD-RED-CALCUTTA
      - IUMILLO —— LV-ITA
      - MARQUIS
        - RED-FIFE
        - HARD-RED-CALCUTTA
    - FRONTANA
      - MENTANA
        - AKAKOMUGI —— LV-FUKUI
        - WILHELMINA
        - RIETI
      - FRONTEIRA
        - ALFREDO-CHAVES-6-21 —— (S) TURCO
        - POLYSSU —— LV-RIO-GRANDE-DO-SUL
  - FORTUNA
    - SORATNITSA
      - PARTIZANKA
        - NS-116
          - HEINES-VII
            - KRON
            - HYBRIDE-A-COURTE-PAILLE
          - CAMPODORO,TR.AE
            - MARA,ITA
            - FRECCIA
              - AQUILA,ITA
              - AUTONOMIA
        - BEZOSTAYA-1
          - SKOROSPELKA-2
            - KLEIN-33
            - FULCASTER
            - KANRED
          - LUTESCENS-17,UKR
            - UKRAINKA
            - T-389
      - ODESSKAYA-66
        - ODESSKAYA-51
          - BEZOSTAYA-1
            - SKOROSPELKA-2
            - LUTESCENS-17,UKR
          - ODESSKAYA-16 —— (S) ODESSKAYA-12
        - KAVKAZ
          - BEZOSTAYA-1
            - SKOROSPELKA-2
            - LUTESCENS-17,UKR
          - LUTESCENS-314-h-147
            - BEZOSTAYA-1
            - BEZOSTAYA-4
            - NEUZUCHT-14-44
    - KN-1985-h-124
      - KN-6203-M-691
      - KN-2574-G-250-sm-11
        - RUBIN,BGR
          - BEZOSTAYA-1
            - SKOROSPELKA-2
            - LUTESCENS-17,UKR
          - NS-171-2
            - MARA
            - SAN-PASTORE
            - SELKIRK
            - U-1
        - LUTESCENS-501-h-1
          - KRASNODARSKAYA-39
            - BEZOSTAYA-1
            - SARATOVSKAYA-3
          - LUTESCENS-820-h-745
          - STEPNAYA-40

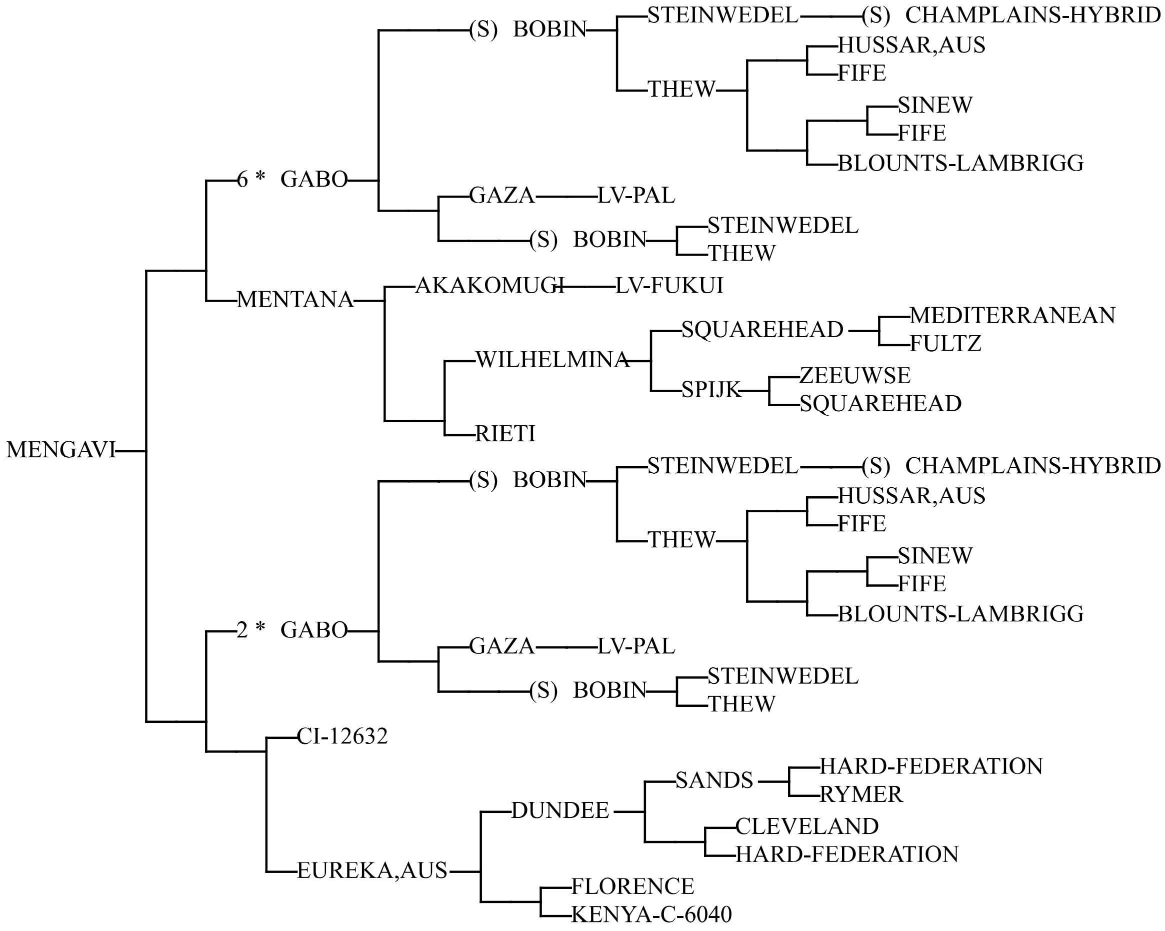
MENGAVI
6 * GABO
(S) BOBIN
STEINWEDEL
(S) CHAMPLAINS-HYBRID
THEW
HUSSAR,AUS
FIFE
SINEW
FIFE
BLOUNTS-LAMBRIGG
GAZA
LV-PAL
(S) BOBIN
STEINWEDEL
THEW
MENTANA
AKAKOMUGI
LV-FUKUI
WILHELMINA
SQUAREHEAD
MEDITERRANEAN
FULTZ
SPIJK
ZEEUWSE
SQUAREHEAD
RIETI
2 * GABO
(S) BOBIN
STEINWEDEL
(S) CHAMPLAINS-HYBRID
THEW
HUSSAR,AUS
FIFE
SINEW
FIFE
BLOUNTS-LAMBRIGG
GAZA
LV-PAL
(S) BOBIN
STEINWEDEL
THEW
CI-12632
EUREKA,AUS
DUNDEE
SANDS
HARD-FEDERATION
RYMER
CLEVELAND
HARD-FEDERATION
FLORENCE
KENYA-C-6040

- NEWTHATCH
  - 2 * THATCHER
    - KANRED —— (S) CRIMEAN —— LV-RUS
    - MARQUIS
      - RED-FIFE —— (S) OSTKA-GALICYJSKA —— LV-POL
      - HARD-RED-CALCUTTA —— LV-IND
    - IUMILLO —— LV-ITA
    - MARQUIS
      - RED-FIFE —— (S) OSTKA-GALICYJSKA —— LV-POL
      - HARD-RED-CALCUTTA —— LV-IND
  - THATCHER
    - KANRED —— (S) CRIMEAN —— LV-RUS
    - MARQUIS
      - RED-FIFE —— (S) OSTKA-GALICYJSKA
      - HARD-RED-CALCUTTA —— LV-IND
    - IUMILLO —— LV-ITA
    - MARQUIS
      - RED-FIFE —— (S) OSTKA-GALICYJSKA
      - HARD-RED-CALCUTTA —— LV-IND
  - HOPE
    - MARQUIS
      - RED-FIFE —— (S) OSTKA-GALICYJSKA —— LV-POL
      - HARD-RED-CALCUTTA —— LV-IND
    - YAROSLAV-EMMER —— LV-RUS,YAROSLAVL

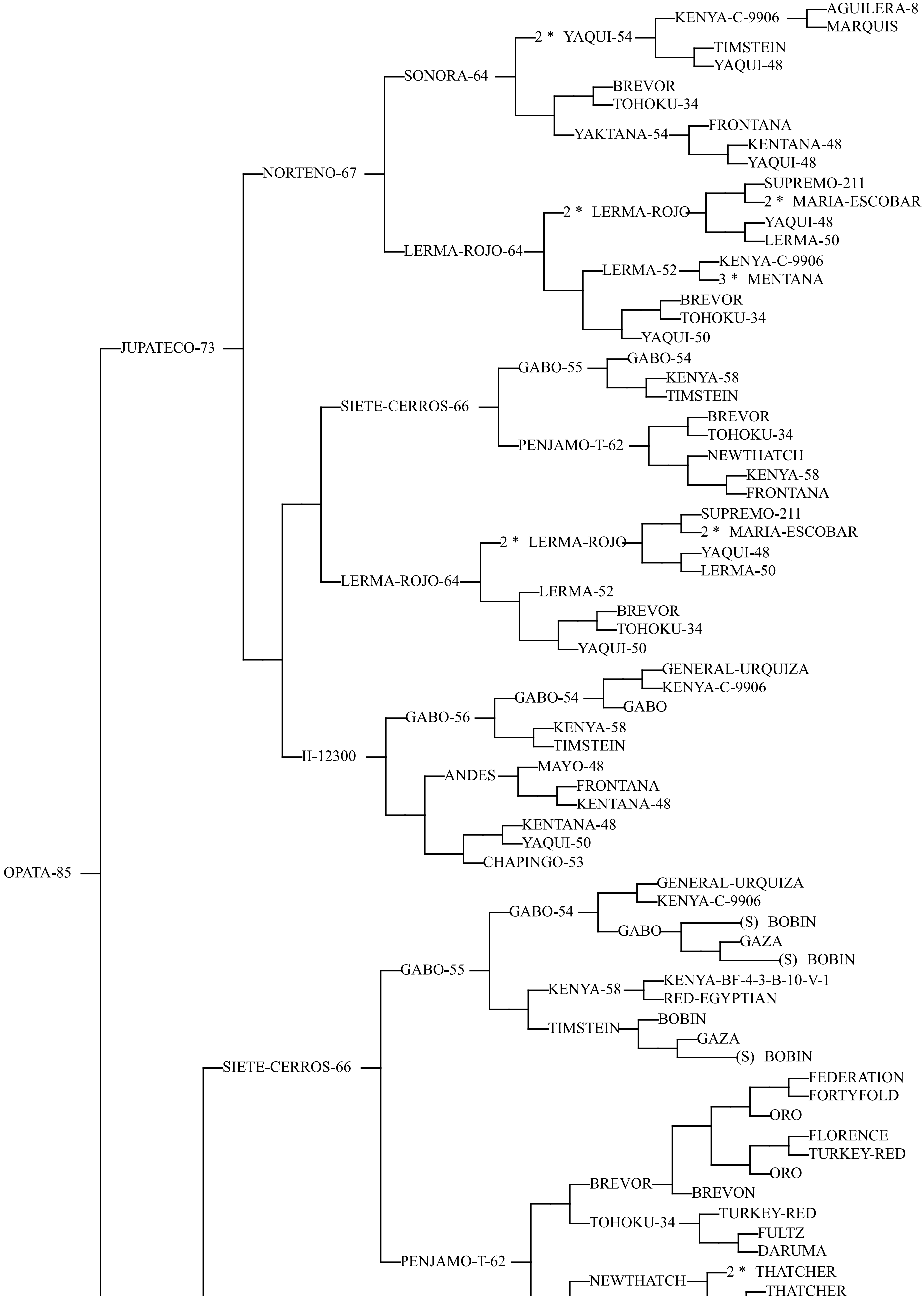
AGUILERA-8
KENYA-C-9906
MARQUIS
2 * YAQUI-54
TIMSTEIN
YAQUI-48
SONORA-64
BREVOR
TOHOKU-34
FRONTANA
YAKTANA-54
KENTANA-48
YAQUI-48
NORTENO-67
SUPREMO-211
2 * MARIA-ESCOBAR
2 * LERMA-ROJO
YAQUI-48
LERMA-50
LERMA-ROJO-64
KENYA-C-9906
LERMA-52
3 * MENTANA
BREVOR
TOHOKU-34
YAQUI-50
JUPATECO-73
GABO-54
GABO-55
KENYA-58
TIMSTEIN
SIETE-CERROS-66
BREVOR
TOHOKU-34
PENJAMO-T-62
NEWTHATCH
KENYA-58
FRONTANA
SUPREMO-211
2 * MARIA-ESCOBAR
2 * LERMA-ROJO
YAQUI-48
LERMA-50
LERMA-ROJO-64
LERMA-52
BREVOR
TOHOKU-34
YAQUI-50
GENERAL-URQUIZA
KENYA-C-9906
GABO-54
GABO
GABO-56
KENYA-58
TIMSTEIN
II-12300
ANDES
MAYO-48
FRONTANA
KENTANA-48
KENTANA-48
YAQUI-50
CHAPINGO-53
OPATA-85
GENERAL-URQUIZA
KENYA-C-9906
GABO-54
GABO
(S) BOBIN
GAZA
(S) BOBIN
GABO-55
KENYA-58
KENYA-BF-4-3-B-10-V-1
RED-EGYPTIAN
TIMSTEIN
BOBIN
GAZA
(S) BOBIN
SIETE-CERROS-66
FEDERATION
FORTYFOLD
ORO
FLORENCE
TURKEY-RED
ORO
BREVOR
BREVON
TOHOKU-34
TURKEY-RED
FULTZ
DARUMA
PENJAMO-T-62
NEWTHATCH
2 * THATCHER
THATCHER

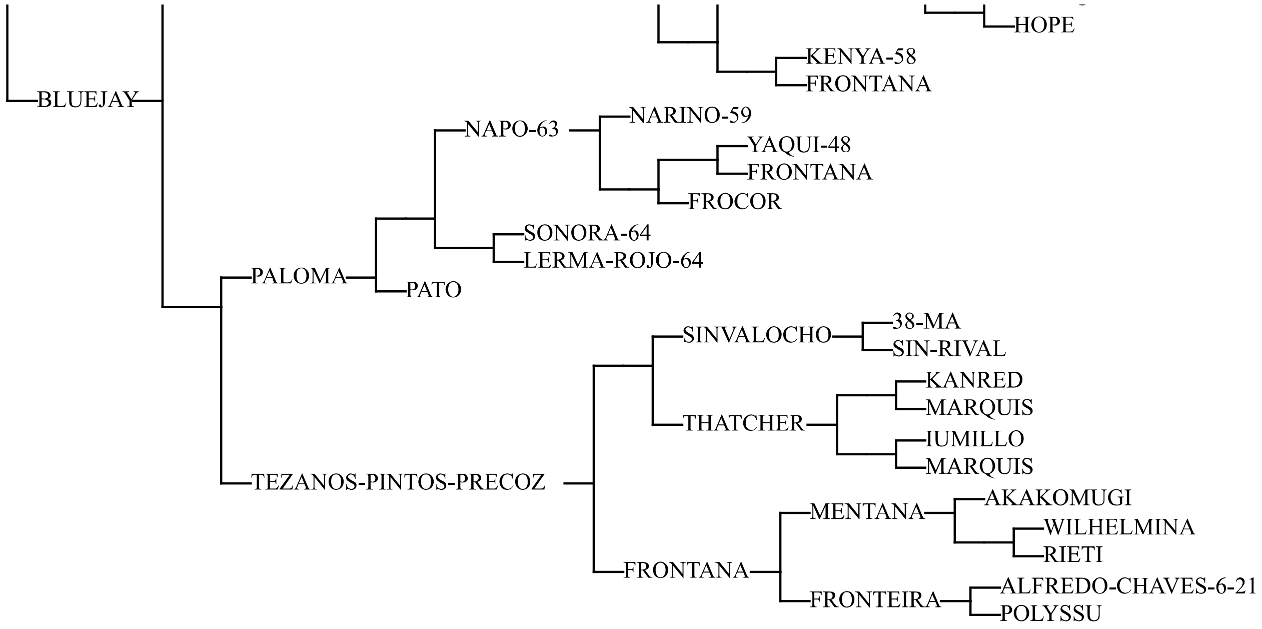
HOPE
KENYA-58
FRONTANA
BLUEJAY
NAPO-63
NARINO-59
YAQUI-48
FRONTANA
FROCOR
SONORA-64
LERMA-ROJO-64
PALOMA
PATO
SINVALOCHO
38-MA
SIN-RIVAL
KANRED
MARQUIS
THATCHER
IUMILLO
MARQUIS
TEZANOS-PINTOS-PRECOZ
MENTANA
AKAKOMUGI
WILHELMINA
RIETI
FRONTANA
FRONTEIRA
ALFREDO-CHAVES-6-21
POLYSSU

OS21-5:

Tordo/Zenkojikomugi
or
Tordo/Zenkoji

Osanai et al. 2015

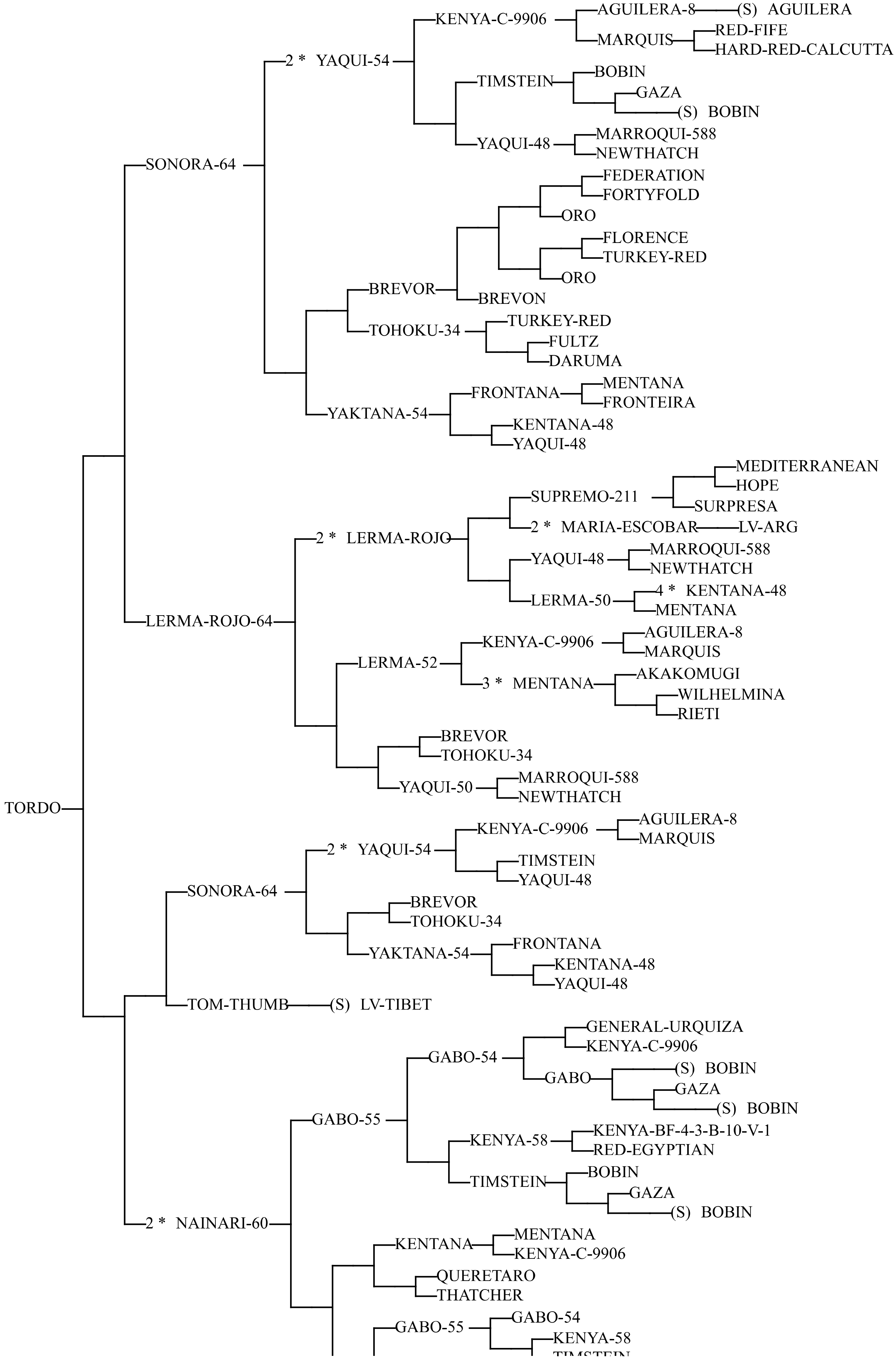
TORDO
SONORA-64
2 * YAQUI-54
KENYA-C-9906
AGUILERA-8
(S) AGUILERA
MARQUIS
RED-FIFE
HARD-RED-CALCUTTA
TIMSTEIN
BOBIN
GAZA
(S) BOBIN
YAQUI-48
MARROQUI-588
NEWTHATCH
FEDERATION
FORTYFOLD
ORO
FLORENCE
TURKEY-RED
ORO
BREVOR
BREVON
TOHOKU-34
TURKEY-RED
FULTZ
DARUMA
FRONTANA
MENTANA
FRONTEIRA
YAKTANA-54
KENTANA-48
YAQUI-48
LERMA-ROJO-64
2 * LERMA-ROJO
SUPREMO-211
MEDITERRANEAN
HOPE
SURPRESA
2 * MARIA-ESCOBAR
LV-ARG
YAQUI-48
MARROQUI-588
NEWTHATCH
LERMA-50
4 * KENTANA-48
MENTANA
LERMA-52
KENYA-C-9906
AGUILERA-8
MARQUIS
3 * MENTANA
AKAKOMUGI
WILHELMINA
RIETI
BREVOR
TOHOKU-34
YAQUI-50
MARROQUI-588
NEWTHATCH
SONORA-64
2 * YAQUI-54
KENYA-C-9906
AGUILERA-8
MARQUIS
TIMSTEIN
YAQUI-48
BREVOR
TOHOKU-34
YAKTANA-54
FRONTANA
KENTANA-48
YAQUI-48
TOM-THUMB
(S) LV-TIBET
2 * NAINARI-60
GABO-55
GABO-54
GENERAL-URQUIZA
KENYA-C-9906
GABO
(S) BOBIN
GAZA
(S) BOBIN
KENYA-58
KENYA-BF-4-3-B-10-V-1
RED-EGYPTIAN
TIMSTEIN
BOBIN
GAZA
(S) BOBIN
KENTANA
MENTANA
KENYA-C-9906
QUERETARO
THATCHER
GABO-55
GABO-54
KENYA-58

TIMSTEIN

MENTANA

SUPREMO

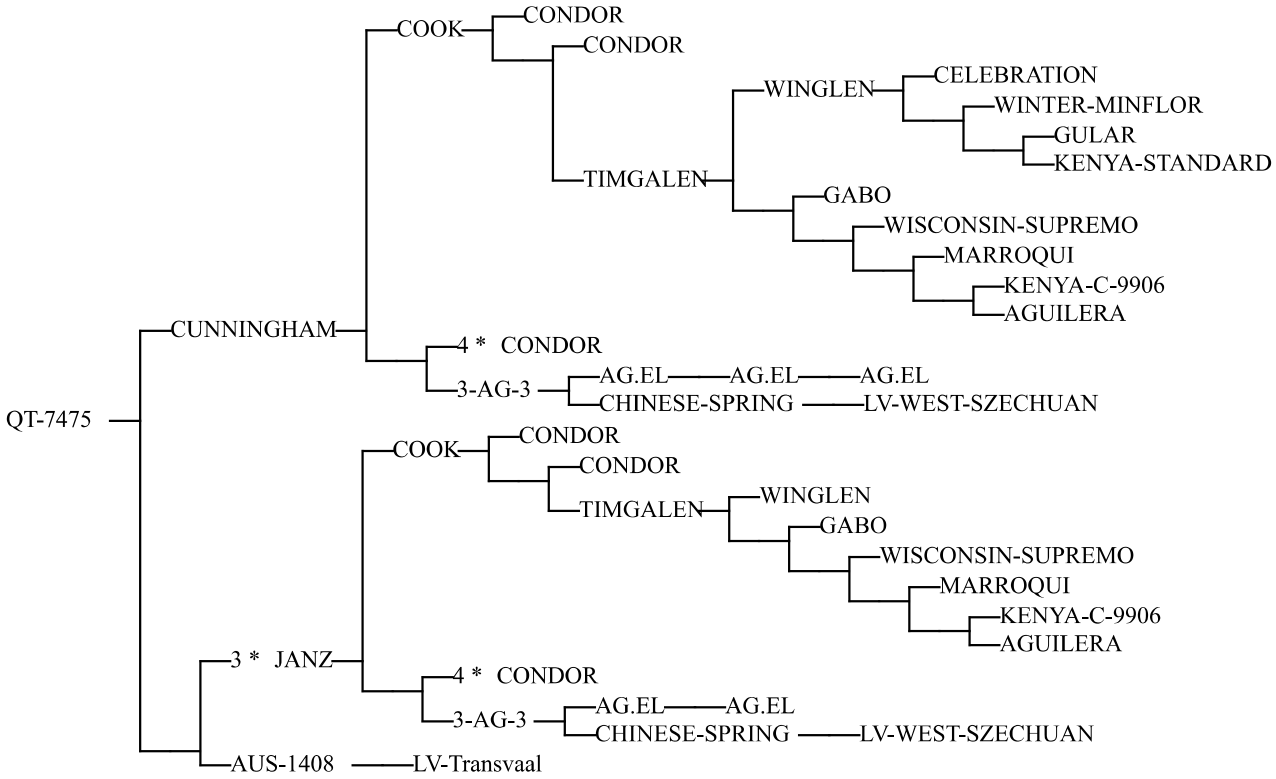
QT-7475
CUNNINGHAM
COOK
CONDOR
CONDOR
TIMGALEN
WINGLEN
CELEBRATION
WINTER-MINFLOR
GULAR
KENYA-STANDARD
GABO
WISCONSIN-SUPREMO
MARROQUI
KENYA-C-9906
AGUILERA
4 * CONDOR
3-AG-3
AG.EL
AG.EL
AG.EL
CHINESE-SPRING
LV-WEST-SZECHUAN
3 * JANZ
COOK
CONDOR
CONDOR
TIMGALEN
WINGLEN
GABO
WISCONSIN-SUPREMO
MARROQUI
KENYA-C-9906
AGUILERA
4 * CONDOR
3-AG-3
AG.EL
AG.EL
CHINESE-SPRING
LV-WEST-SZECHUAN
AUS-1408
LV-Transvaal

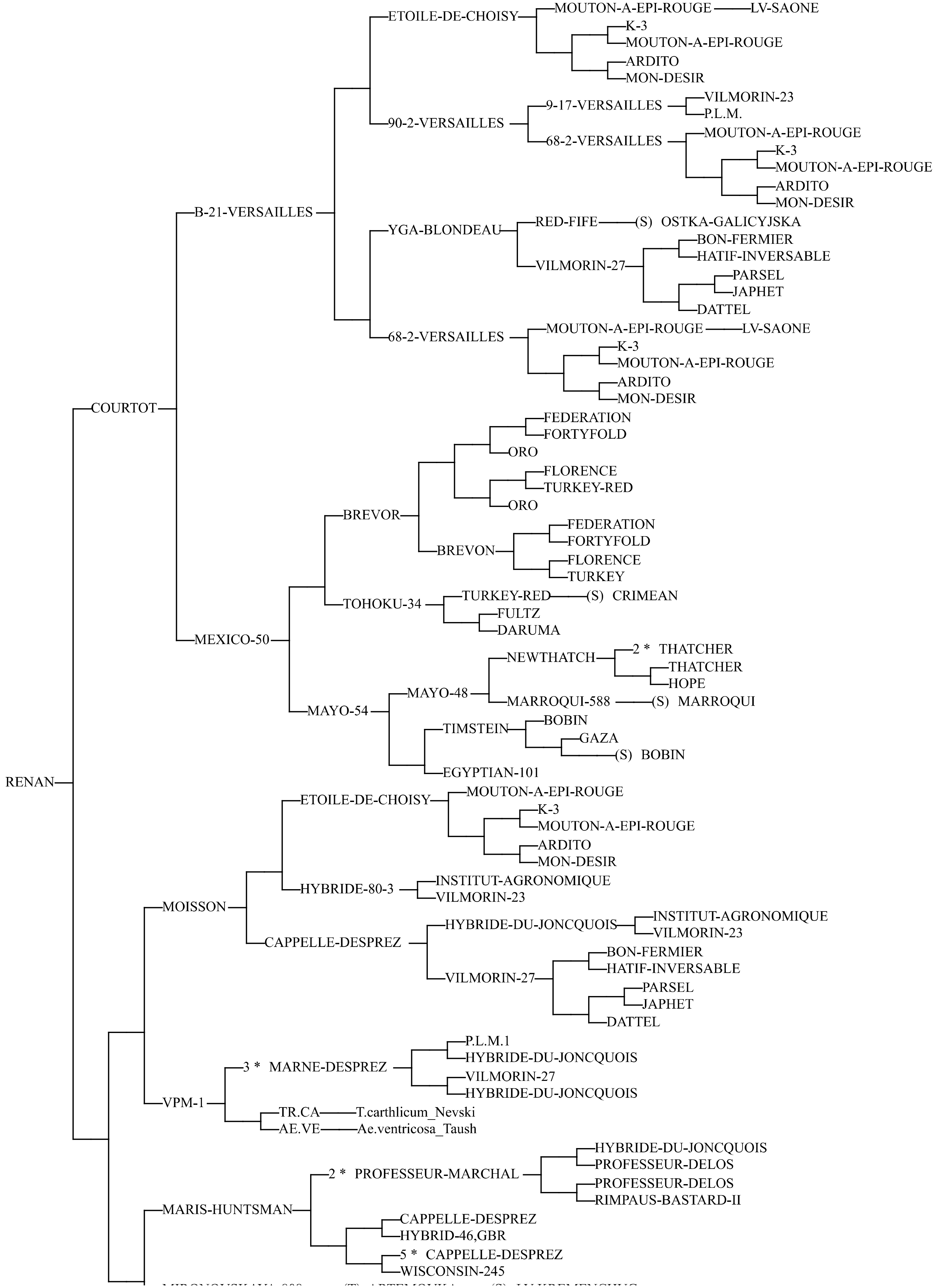
RENAN
COURTOT
B-21-VERSAILLES
ETOILE-DE-CHOISY
MOUTON-A-EPI-ROUGE
LV-SAONE
K-3
MOUTON-A-EPI-ROUGE
ARDITO
MON-DESIR
90-2-VERSAILLES
9-17-VERSAILLES
VILMORIN-23
P.L.M.
68-2-VERSAILLES
MOUTON-A-EPI-ROUGE
K-3
MOUTON-A-EPI-ROUGE
ARDITO
MON-DESIR
YGA-BLONDEAU
RED-FIFE
(S) OSTKA-GALICYJSKA
VILMORIN-27
BON-FERMIER
HATIF-INVERSABLE
PARSEL
JAPHET
DATTEL
68-2-VERSAILLES
MOUTON-A-EPI-ROUGE
LV-SAONE
K-3
MOUTON-A-EPI-ROUGE
ARDITO
MON-DESIR
MEXICO-50
BREVOR
FEDERATION
FORTYFOLD
ORO
FLORENCE
TURKEY-RED
ORO
BREVON
FEDERATION
FORTYFOLD
FLORENCE
TURKEY
TOHOKU-34
TURKEY-RED
(S) CRIMEAN
FULTZ
DARUMA
MAYO-54
MAYO-48
NEWTHATCH
2 * THATCHER
THATCHER
HOPE
MARROQUI-588
(S) MARROQUI
TIMSTEIN
BOBIN
GAZA
(S) BOBIN
EGYPTIAN-101
MOISSON
ETOILE-DE-CHOISY
MOUTON-A-EPI-ROUGE
K-3
MOUTON-A-EPI-ROUGE
ARDITO
MON-DESIR
HYBRIDE-80-3
INSTITUT-AGRONOMIQUE
VILMORIN-23
CAPPELLE-DESPREZ
HYBRIDE-DU-JONCQUOIS
INSTITUT-AGRONOMIQUE
VILMORIN-23
VILMORIN-27
BON-FERMIER
HATIF-INVERSABLE
PARSEL
JAPHET
DATTEL
VPM-1
3 * MARNE-DESPREZ
P.L.M.1
HYBRIDE-DU-JONCQUOIS
VILMORIN-27
HYBRIDE-DU-JONCQUOIS
TR.CA
T.carthlicum_Nevski
AE.VE
Ae.ventricosa_Tausch
MARIS-HUNTSMAN
2 * PROFESSEUR-MARCHAL
HYBRIDE-DU-JONCQUOIS
PROFESSEUR-DELOS
PROFESSEUR-DELOS
RIMPAUS-BASTARD-II
CAPPELLE-DESPREZ
HYBRID-46,GBR
5 * CAPPELLE-DESPREZ
WISCONSIN-245

└─MIRONOVSKAYA-808 ─── (T) ARTEMOVKA ─── (S) LV-KREMENCHUG

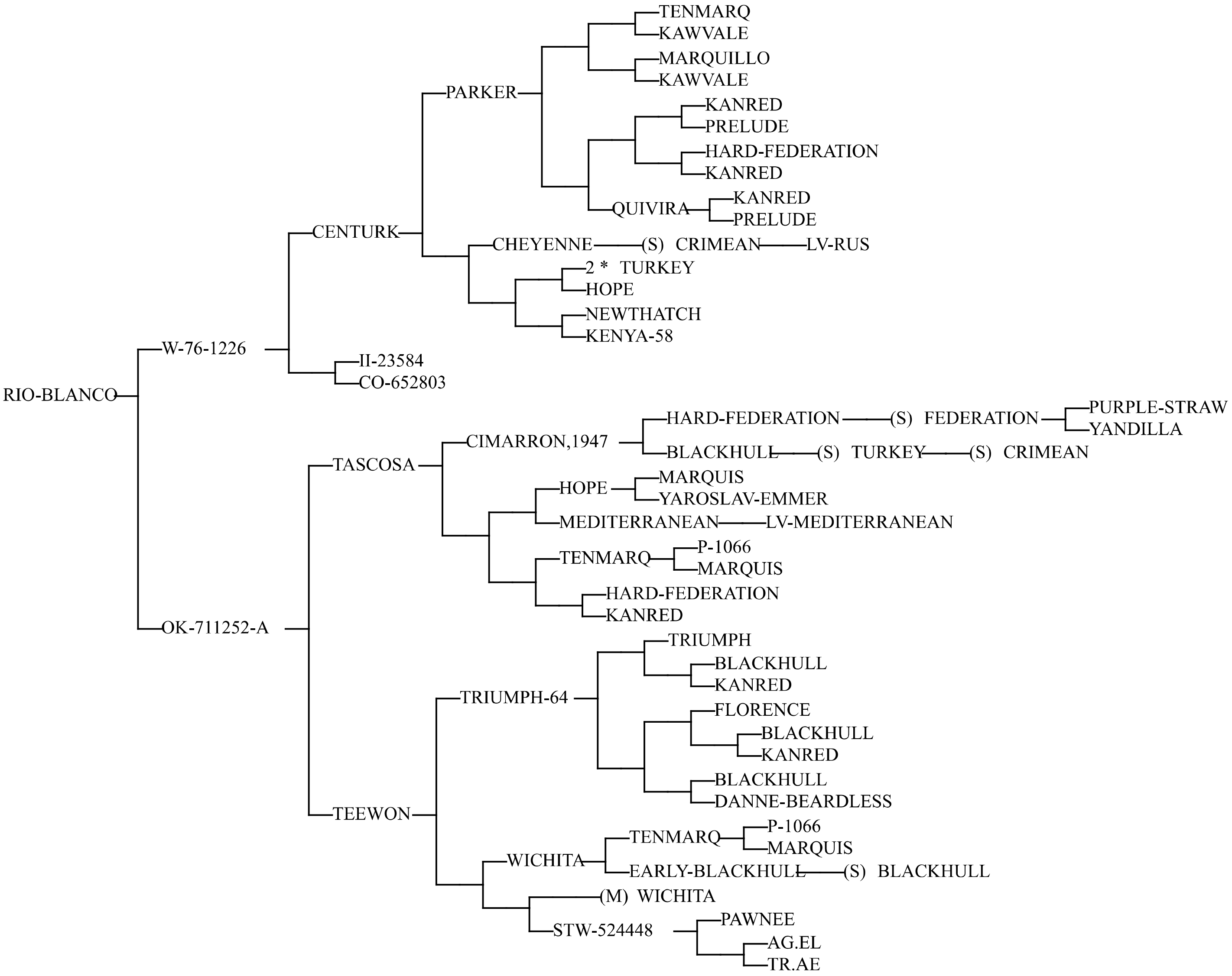
TENMARQ
KAWVALE
MARQUILLO
KAWVALE
PARKER
KANRED
PRELUDE
HARD-FEDERATION
KANRED
QUIVIRA
KANRED
PRELUDE
CENTURK
CHEYENNE
(S) CRIMEAN
LV-RUS
2 * TURKEY
HOPE
NEWTHATCH
KENYA-58
W-76-1226
II-23584
CO-652803
RIO-BLANCO
HARD-FEDERATION
(S) FEDERATION
PURPLE-STRAW
YANDILLA
CIMARRON,1947
BLACKHULL
(S) TURKEY
(S) CRIMEAN
TASCOSA
HOPE
MARQUIS
YAROSLAV-EMMER
MEDITERRANEAN
LV-MEDITERRANEAN
TENMARQ
P-1066
MARQUIS
HARD-FEDERATION
KANRED
OK-711252-A
TRIUMPH
BLACKHULL
KANRED
TRIUMPH-64
FLORENCE
BLACKHULL
KANRED
BLACKHULL
DANNE-BEARDLESS
TEEWON
TENMARQ
P-1066
MARQUIS
WICHITA
EARLY-BLACKHULL
(S) BLACKHULL
(M) WICHITA
STW-524448
PAWNEE
AG.EL
TR.AE

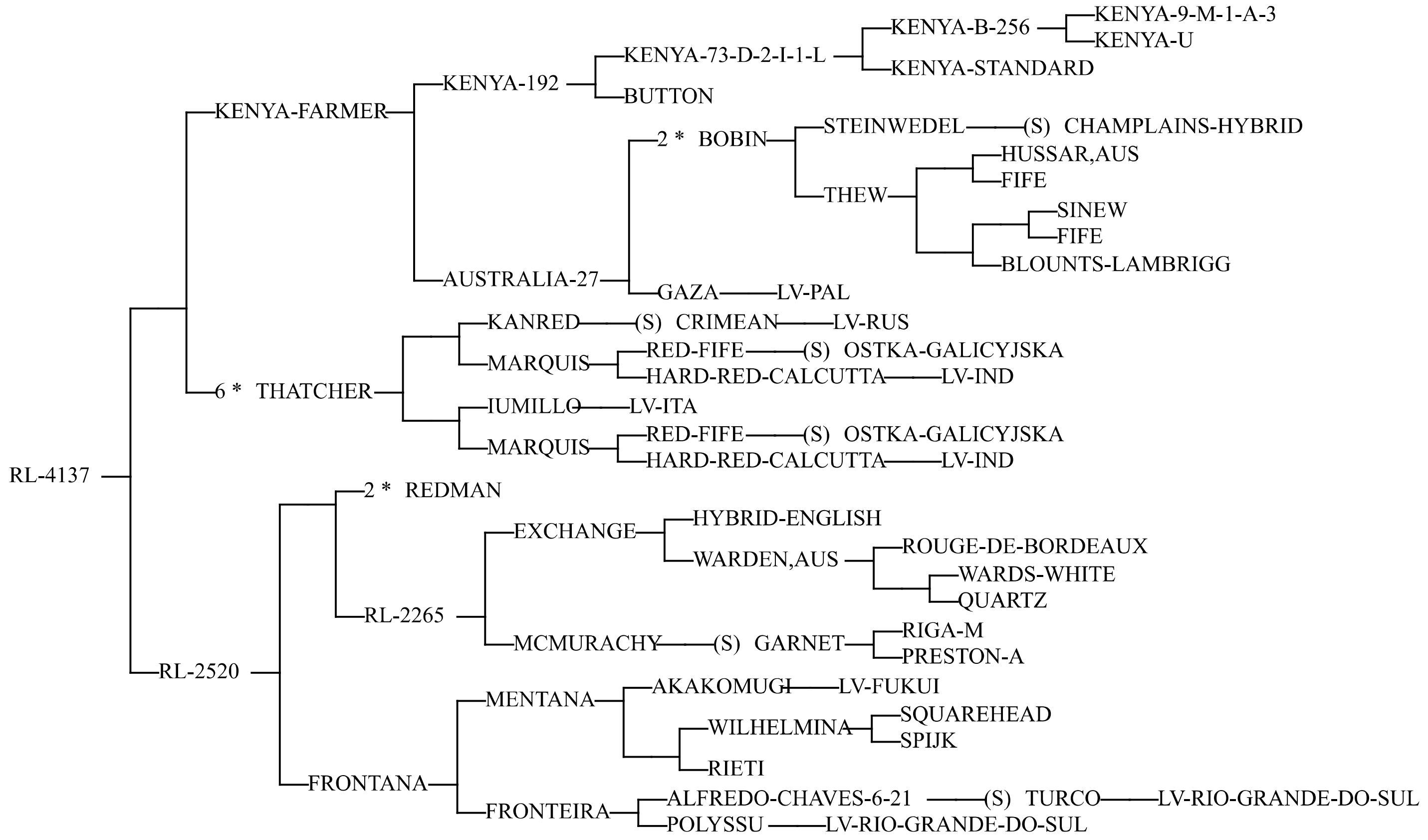
RL-4137
KENYA-FARMER
KENYA-192
KENYA-73-D-2-I-1-L
KENYA-B-256
KENYA-9-M-1-A-3
KENYA-U
KENYA-STANDARD
BUTTON
AUSTRALIA-27
2 * BOBIN
STEINWEDEL
(S) CHAMPLAINS-HYBRID
THEW
HUSSAR,AUS
FIFE
SINEW
FIFE
BLOUNTS-LAMBRIGG
GAZA
LV-PAL
6 * THATCHER
KANRED
(S) CRIMEAN
LV-RUS
MARQUIS
RED-FIFE
(S) OSTKA-GALICYJSKA
HARD-RED-CALCUTTA
LV-IND
IUMILLO
LV-ITA
MARQUIS
RED-FIFE
(S) OSTKA-GALICYJSKA
HARD-RED-CALCUTTA
LV-IND
RL-2520
2 * REDMAN
RL-2265
EXCHANGE
HYBRID-ENGLISH
WARDEN,AUS
ROUGE-DE-BORDEAUX
WARDS-WHITE
QUARTZ
MCMURACHY
(S) GARNET
RIGA-M
PRESTON-A
FRONTANA
MENTANA
AKAKOMUGI
LV-FUKUI
WILHELMINA
SQUAREHEAD
SPIJK
RIETI
FRONTEIRA
ALFREDO-CHAVES-6-21
(S) TURCO
LV-RIO-GRANDE-DO-SUL
POLYSSU
LV-RIO-GRANDE-DO-SUL

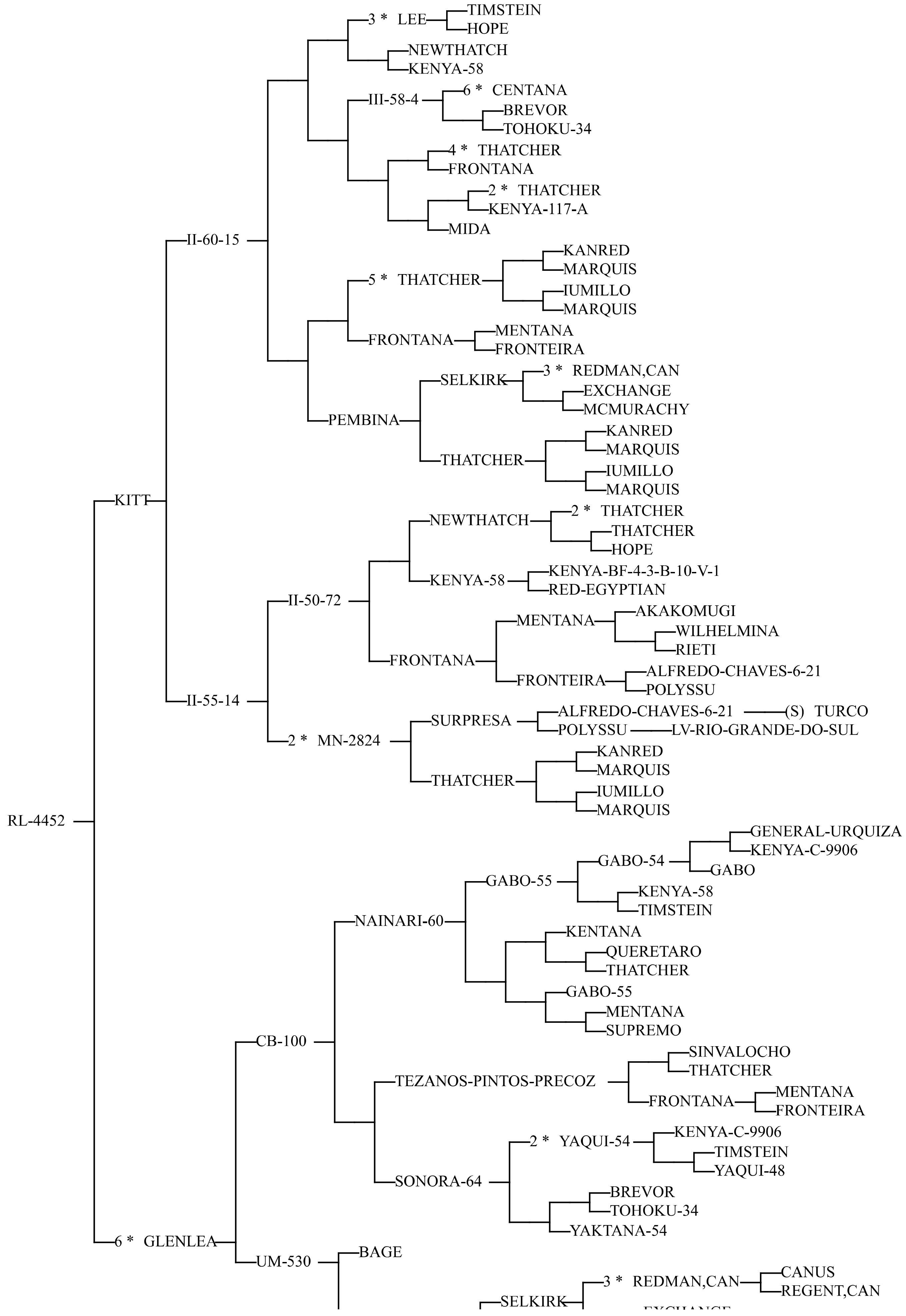
RL-4452
KITT
II-60-15
3 * LEE
TIMSTEIN
HOPE
NEWTHATCH
KENYA-58
III-58-4
6 * CENTANA
BREVOR
TOHOKU-34
4 * THATCHER
FRONTANA
2 * THATCHER
KENYA-117-A
MIDA
5 * THATCHER
KANRED
MARQUIS
IUMILLO
MARQUIS
FRONTANA
MENTANA
FRONTEIRA
PEMBINA
SELKIRK
3 * REDMAN,CAN
EXCHANGE
MCMURACHY
THATCHER
KANRED
MARQUIS
IUMILLO
MARQUIS
II-55-14
II-50-72
NEWTHATCH
2 * THATCHER
THATCHER
HOPE
KENYA-58
KENYA-BF-4-3-B-10-V-1
RED-EGYPTIAN
FRONTANA
MENTANA
AKAKOMUGI
WILHELMINA
RIETI
FRONTEIRA
ALFREDO-CHAVES-6-21
POLYSSU
2 * MN-2824
SURPRESA
ALFREDO-CHAVES-6-21
(S) TURCO
POLYSSU
LV-RIO-GRANDE-DO-SUL
THATCHER
KANRED
MARQUIS
IUMILLO
MARQUIS
6 * GLENLEA
CB-100
NAINARI-60
GABO-55
GABO-54
GENERAL-URQUIZA
KENYA-C-9906
GABO
KENYA-58
TIMSTEIN
KENTANA
QUERETARO
THATCHER
GABO-55
MENTANA
SUPREMO
TEZANOS-PINTOS-PRECOZ
SINVALOCHO
THATCHER
FRONTANA
MENTANA
FRONTEIRA
SONORA-64
2 * YAQUI-54
KENYA-C-9906
TIMSTEIN
YAQUI-48
BREVOR
TOHOKU-34
YAKTANA-54
UM-530
BAGE
SELKIRK
3 * REDMAN,CAN
CANUS
REGENT,CAN

EXCHANGE
MCMURACHY
2 * PEMBINA
KANRED
MARQUIS
THATCHER
IUMILLO
MARQUIS

RSP ─┬─ LV-HENAN
     └─ AILANMAI ─── LV-Sichuan

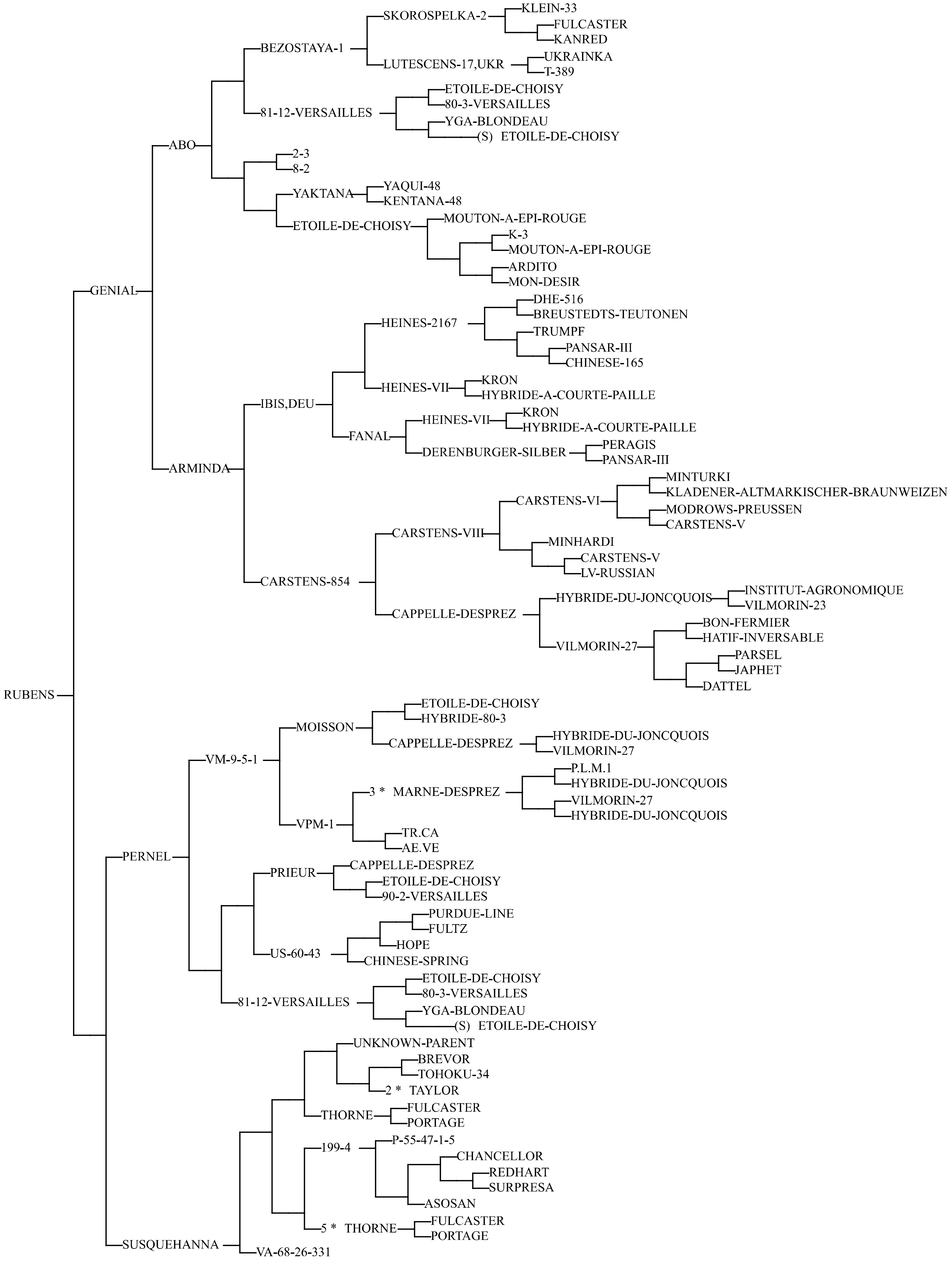
RUBENS
GENIAL
ABO
BEZOSTAYA-1
SKOROSPELKA-2
KLEIN-33
FULCASTER
KANRED
LUTESCENS-17,UKR
UKRAINKA
T-389
81-12-VERSAILLES
ETOILE-DE-CHOISY
80-3-VERSAILLES
YGA-BLONDEAU
(S) ETOILE-DE-CHOISY
2-3
8-2
YAKTANA
YAQUI-48
KENTANA-48
ETOILE-DE-CHOISY
MOUTON-A-EPI-ROUGE
K-3
MOUTON-A-EPI-ROUGE
ARDITO
MON-DESIR
ARMINDA
IBIS,DEU
HEINES-2167
DHE-516
BREUSTEDTS-TEUTONEN
TRUMPF
PANSAR-III
CHINESE-165
HEINES-VII
KRON
HYBRIDE-A-COURTE-PAILLE
FANAL
HEINES-VII
KRON
HYBRIDE-A-COURTE-PAILLE
DERENBURGER-SILBER
PERAGIS
PANSAR-III
CARSTENS-854
CARSTENS-VIII
CARSTENS-VI
MINTURKI
KLADENER-ALTMARKISCHER-BRAUNWEIZEN
MODROWS-PREUSSEN
CARSTENS-V
MINHARDI
CARSTENS-V
LV-RUSSIAN
CAPPELLE-DESPREZ
HYBRIDE-DU-JONCQUOIS
INSTITUT-AGRONOMIQUE
VILMORIN-23
VILMORIN-27
BON-FERMIER
HATIF-INVERSABLE
PARSEL
JAPHET
DATTEL
PERNEL
VM-9-5-1
MOISSON
ETOILE-DE-CHOISY
HYBRIDE-80-3
CAPPELLE-DESPREZ
HYBRIDE-DU-JONCQUOIS
VILMORIN-27
VPM-1
3 * MARNE-DESPREZ
P.L.M.1
HYBRIDE-DU-JONCQUOIS
VILMORIN-27
HYBRIDE-DU-JONCQUOIS
TR.CA
AE.VE
PRIEUR
CAPPELLE-DESPREZ
ETOILE-DE-CHOISY
90-2-VERSAILLES
US-60-43
PURDUE-LINE
FULTZ
HOPE
CHINESE-SPRING
81-12-VERSAILLES
ETOILE-DE-CHOISY
80-3-VERSAILLES
YGA-BLONDEAU
(S) ETOILE-DE-CHOISY
SUSQUEHANNA
UNKNOWN-PARENT
BREVOR
TOHOKU-34
2 * TAYLOR
THORNE
FULCASTER
PORTAGE
199-4
P-55-47-1-5
CHANCELLOR
REDHART
SURPRESA
ASOSAN
5 * THORNE
FULCASTER
PORTAGE
VA-68-26-331

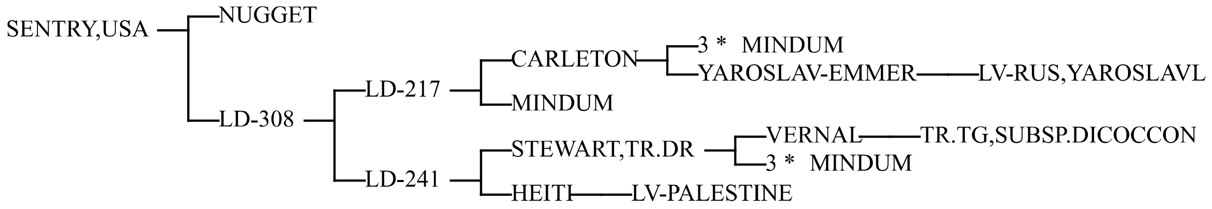
SENTRY,USA
NUGGET
LD-308
LD-217
CARLETON
3 * MINDUM
YAROSLAV-EMMER
LV-RUS,YAROSLAVL
MINDUM
LD-241
STEWART,TR.DR
VERNAL
TR.TG,SUBSP.DICOCCON
3 * MINDUM
HEITI
LV-PALESTINE

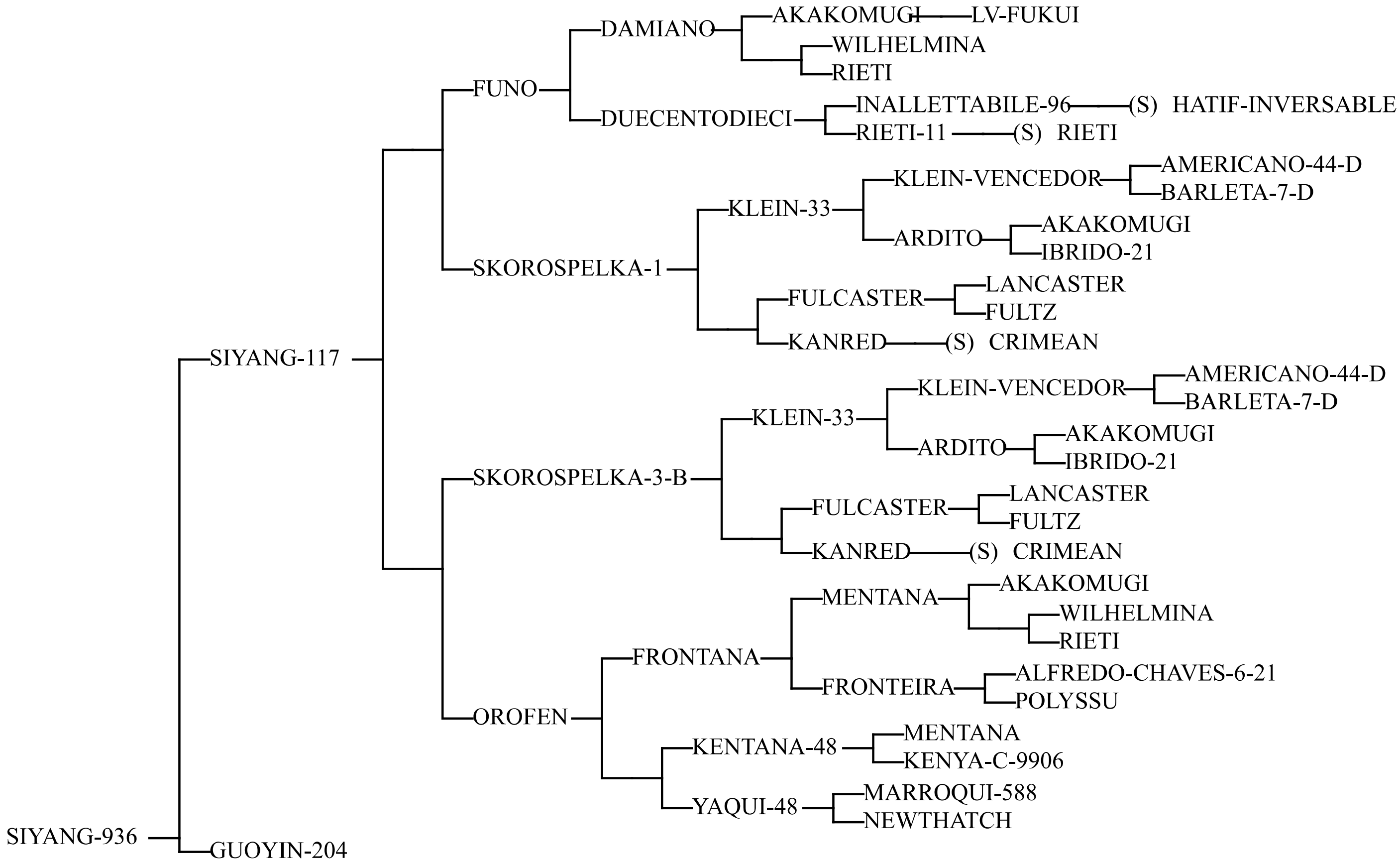
SIYANG-936
SIYANG-117
GUOYIN-204
FUNO
SKOROSPELKA-1
SKOROSPELKA-3-B
OROFEN
DAMIANO
DUECENTODIECI
AKAKOMUGI
LV-FUKUI
WILHELMINA
RIETI
INALLETTABILE-96
(S) HATIF-INVERSABLE
RIETI-11
(S) RIETI
KLEIN-33
KLEIN-VENCEDOR
AMERICANO-44-D
BARLETA-7-D
ARDITO
AKAKOMUGI
IBRIDO-21
FULCASTER
LANCASTER
FULTZ
KANRED
(S) CRIMEAN
KLEIN-33
KLEIN-VENCEDOR
AMERICANO-44-D
BARLETA-7-D
ARDITO
AKAKOMUGI
IBRIDO-21
FULCASTER
LANCASTER
FULTZ
KANRED
(S) CRIMEAN
FRONTANA
MENTANA
AKAKOMUGI
WILHELMINA
RIETI
FRONTEIRA
ALFREDO-CHAVES-6-21
POLYSSU
KENTANA-48
MENTANA
KENYA-C-9906
YAQUI-48
MARROQUI-588
NEWTHATCH

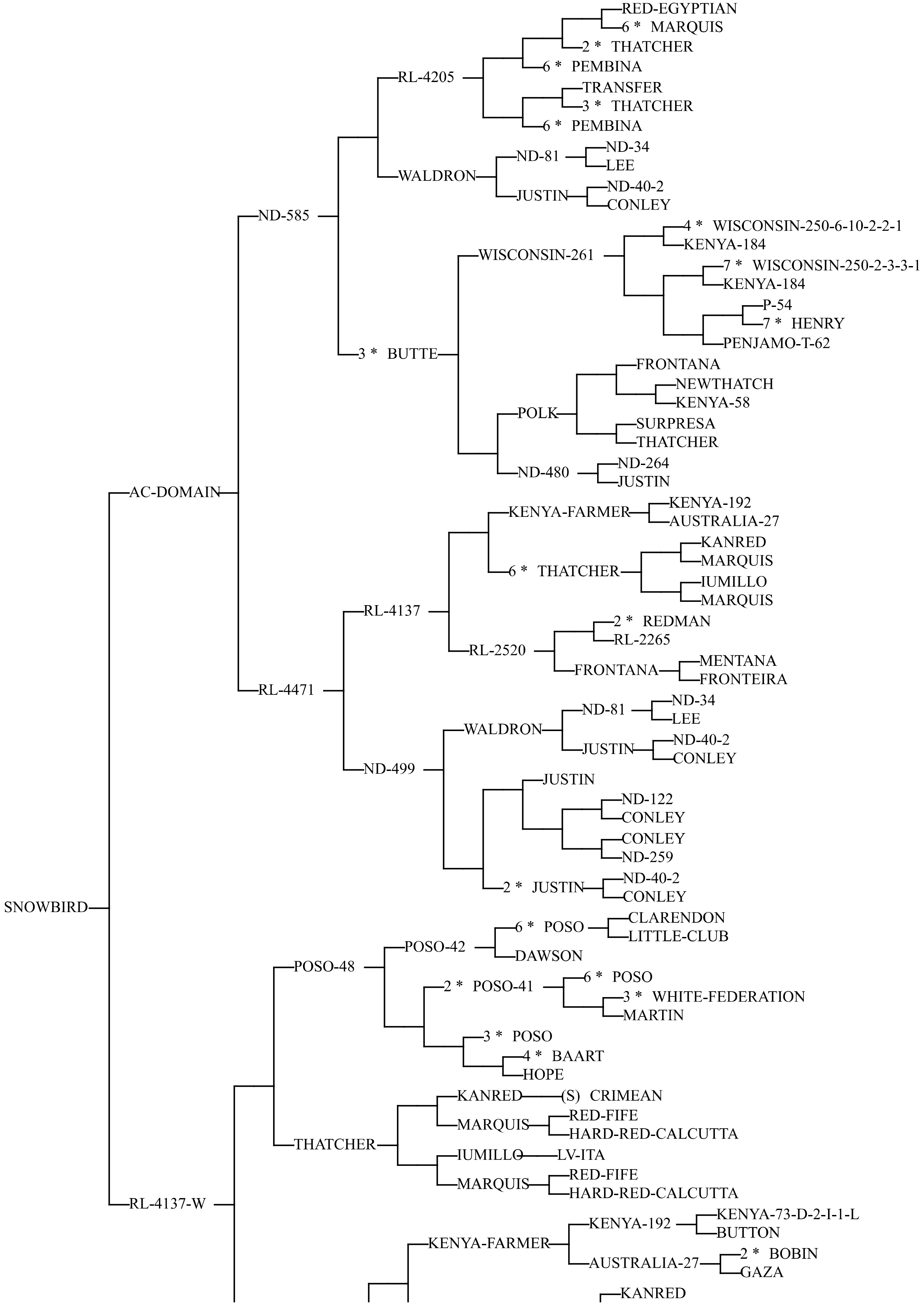
RED-EGYPTIAN
6 * MARQUIS
2 * THATCHER
6 * PEMBINA
RL-4205
TRANSFER
3 * THATCHER
6 * PEMBINA
ND-81
ND-34
LEE
WALDRON
JUSTIN
ND-40-2
CONLEY
ND-585
4 * WISCONSIN-250-6-10-2-2-1
KENYA-184
WISCONSIN-261
7 * WISCONSIN-250-2-3-3-1
KENYA-184
P-54
7 * HENRY
PENJAMO-T-62
3 * BUTTE
FRONTANA
NEWTHATCH
KENYA-58
POLK
SURPRESA
THATCHER
ND-480
ND-264
JUSTIN
AC-DOMAIN
KENYA-FARMER
KENYA-192
AUSTRALIA-27
KANRED
MARQUIS
6 * THATCHER
IUMILLO
MARQUIS
RL-4137
2 * REDMAN
RL-2265
RL-2520
FRONTANA
MENTANA
FRONTEIRA
RL-4471
ND-81
ND-34
LEE
WALDRON
JUSTIN
ND-40-2
CONLEY
ND-499
JUSTIN
ND-122
CONLEY
CONLEY
ND-259
2 * JUSTIN
ND-40-2
CONLEY
SNOWBIRD
6 * POSO
CLARENDON
LITTLE-CLUB
POSO-42
DAWSON
POSO-48
2 * POSO-41
6 * POSO
3 * WHITE-FEDERATION
MARTIN
3 * POSO
4 * BAART
HOPE
KANRED
(S) CRIMEAN
MARQUIS
RED-FIFE
HARD-RED-CALCUTTA
THATCHER
IUMILLO
LV-ITA
MARQUIS
RED-FIFE
HARD-RED-CALCUTTA
RL-4137-W
KENYA-192
KENYA-73-D-2-I-1-L
BUTTON
KENYA-FARMER
AUSTRALIA-27
2 * BOBIN
GAZA
KANRED

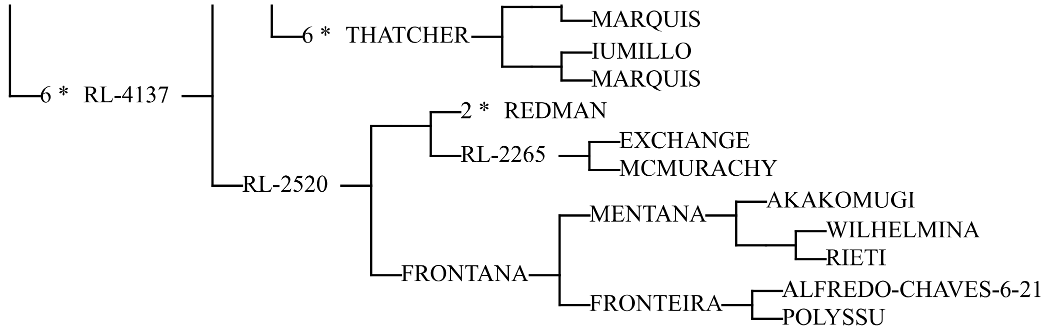
6 * THATCHER
MARQUIS
IUMILLO
MARQUIS
6 * RL-4137
2 * REDMAN
RL-2265
EXCHANGE
MCMURACHY
RL-2520
MENTANA
AKAKOMUGI
WILHELMINA
RIETI
FRONTANA
FRONTEIRA
ALFREDO-CHAVES-6-21
POLYSSU

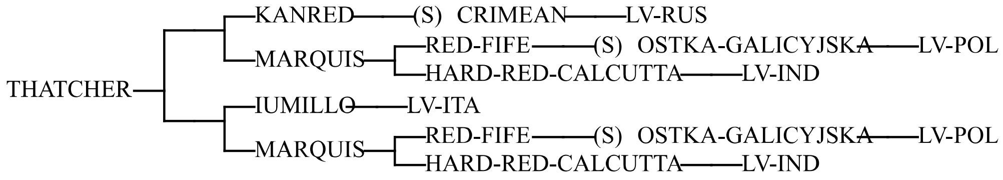
THATCHER
KANRED
(S) CRIMEAN
LV-RUS
MARQUIS
RED-FIFE
(S) OSTKA-GALICYJSKA
LV-POL
HARD-RED-CALCUTTA
LV-IND
IUMILLO
LV-ITA
MARQUIS
RED-FIFE
(S) OSTKA-GALICYJSKA
LV-POL
HARD-RED-CALCUTTA
LV-IND

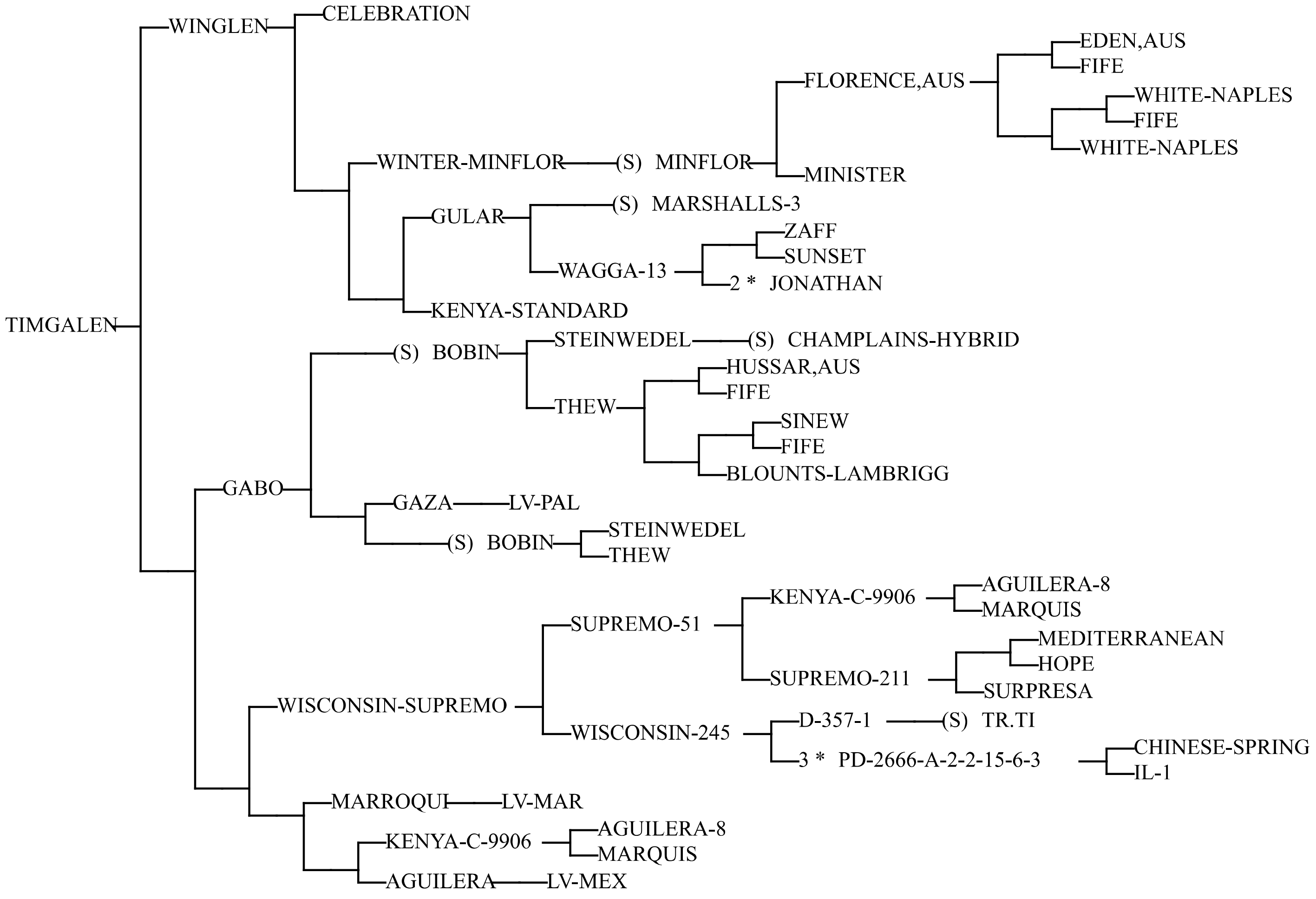
TIMGALEN
WINGLEN
CELEBRATION
WINTER-MINFLOR
(S) MINFLOR
FLORENCE,AUS
EDEN,AUS
FIFE
WHITE-NAPLES
FIFE
WHITE-NAPLES
MINISTER
GULAR
(S) MARSHALLS-3
WAGGA-13
ZAFF
SUNSET
2 * JONATHAN
KENYA-STANDARD
GABO
(S) BOBIN
STEINWEDEL
(S) CHAMPLAINS-HYBRID
THEW
HUSSAR,AUS
FIFE
SINEW
FIFE
BLOUNTS-LAMBRIGG
GAZA
LV-PAL
(S) BOBIN
STEINWEDEL
THEW
WISCONSIN-SUPREMO
SUPREMO-51
KENYA-C-9906
AGUILERA-8
MARQUIS
SUPREMO-211
MEDITERRANEAN
HOPE
SURPRESA
WISCONSIN-245
D-357-1
(S) TR.TI
3 * PD-2666-A-2-2-15-6-3
CHINESE-SPRING
IL-1
MARROQUI
LV-MAR
KENYA-C-9906
AGUILERA-8
MARQUIS
AGUILERA
LV-MEX

YANDA-1817 ——(S) PINGYAO-XIAOBAIMAI——LV-SHANXI

YANGXIAOMAI——LV-CHANG-AN

Yitpi:

Pedigree: (Chamlein* 8156)* (Mengavi*Site Corros) (Chamlein*8156)*Hron)* (Mengavi*Siete Cerros)* Frame.

Garlinge, J. (2005).

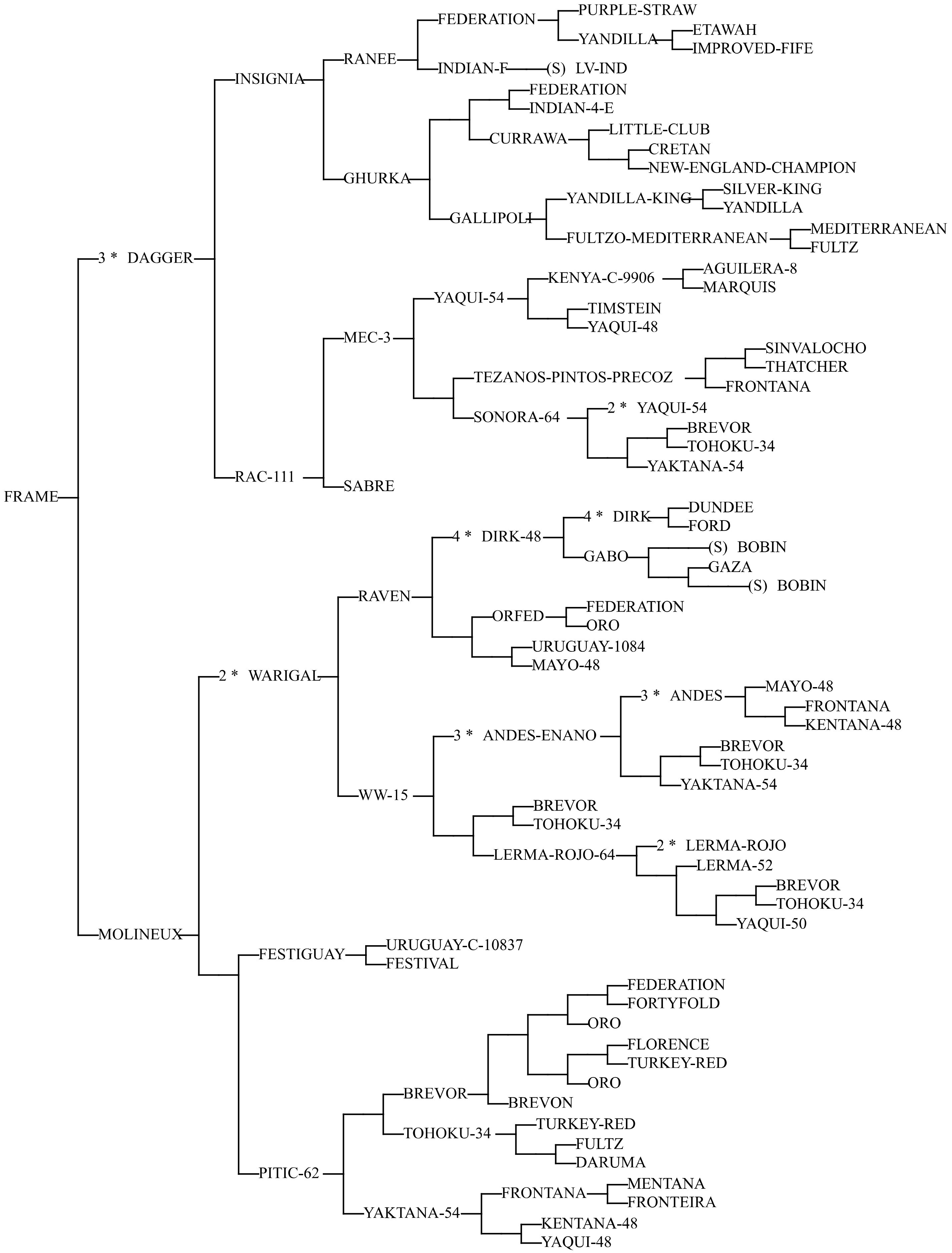
PURPLE-STRAW
FEDERATION
YANDILLA
ETAWAH
IMPROVED-FIFE
RANEE
INDIAN-F
(S) LV-IND
INSIGNIA
FEDERATION
INDIAN-4-E
LITTLE-CLUB
CURRAWA
CRETAN
NEW-ENGLAND-CHAMPION
GHURKA
SILVER-KING
YANDILLA-KING
YANDILLA
GALLIPOLI
MEDITERRANEAN
FULTZO-MEDITERRANEAN
FULTZ
3 * DAGGER
AGUILERA-8
KENYA-C-9906
MARQUIS
YAQUI-54
TIMSTEIN
YAQUI-48
MEC-3
SINVALOCHO
THATCHER
TEZANOS-PINTOS-PRECOZ
FRONTANA
2 * YAQUI-54
SONORA-64
BREVOR
TOHOKU-34
YAKTANA-54
RAC-111
SABRE
FRAME
DUNDEE
4 * DIRK
FORD
4 * DIRK-48
(S) BOBIN
GABO
GAZA
(S) BOBIN
RAVEN
FEDERATION
ORFED
ORO
URUGUAY-1084
MAYO-48
2 * WARIGAL
MAYO-48
3 * ANDES
FRONTANA
KENTANA-48
3 * ANDES-ENANO
BREVOR
TOHOKU-34
YAKTANA-54
WW-15
BREVOR
TOHOKU-34
2 * LERMA-ROJO
LERMA-ROJO-64
LERMA-52
BREVOR
TOHOKU-34
YAQUI-50
MOLINEUX
URUGUAY-C-10837
FESTIGUAY
FESTIVAL
FEDERATION
FORTYFOLD
ORO
FLORENCE
TURKEY-RED
ORO
BREVOR
BREVON
TURKEY-RED
TOHOKU-34
FULTZ
DARUMA
PITIC-62
MENTANA
FRONTANA
FRONTEIRA
YAKTANA-54
KENTANA-48
YAQUI-48

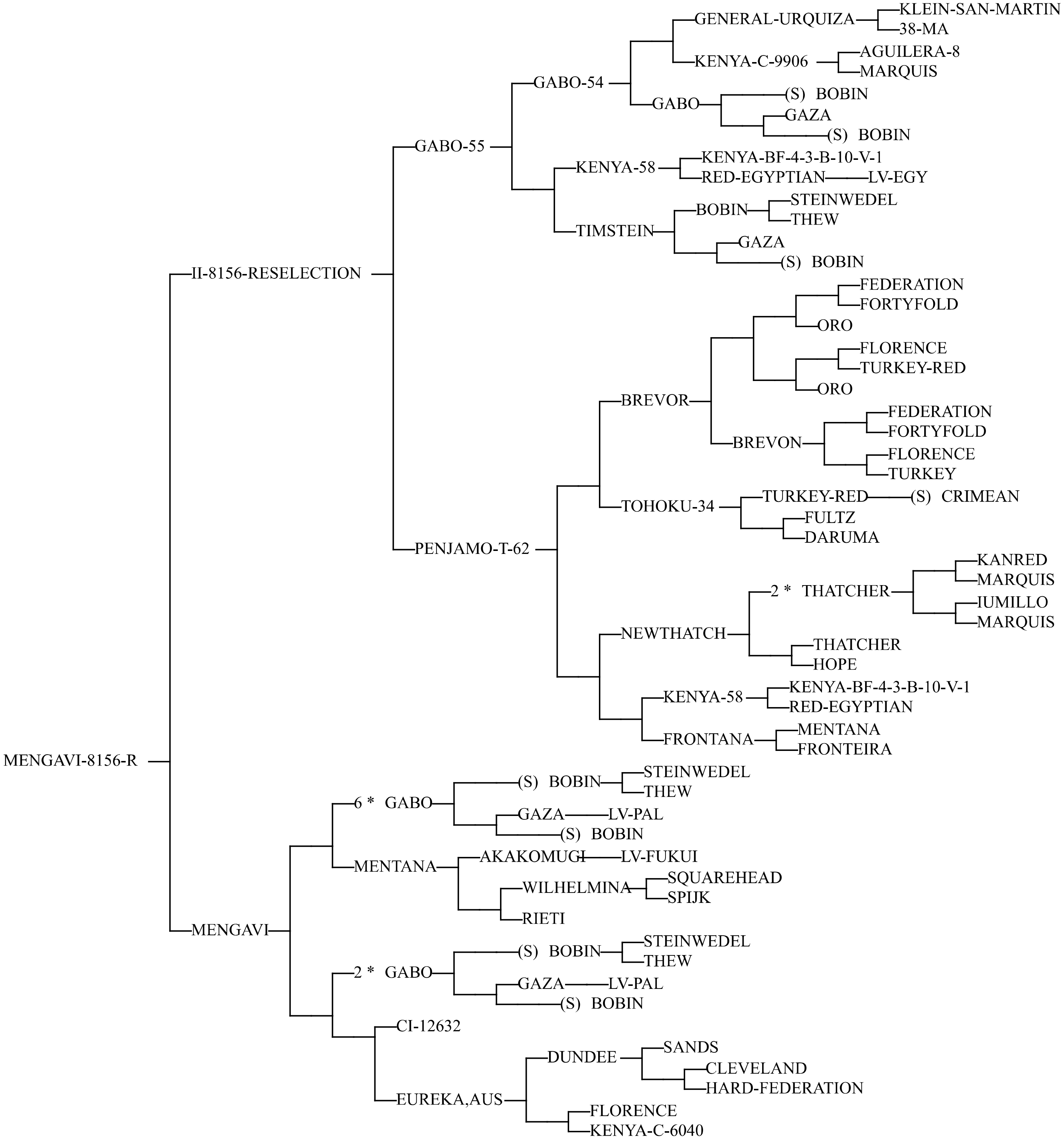
GENERAL-URQUIZA
KLEIN-SAN-MARTIN
38-MA
KENYA-C-9906
AGUILERA-8
MARQUIS
GABO-54
GABO
(S) BOBIN
GAZA
(S) BOBIN
GABO-55
KENYA-58
KENYA-BF-4-3-B-10-V-1
RED-EGYPTIAN
LV-EGY
BOBIN
STEINWEDEL
THEW
TIMSTEIN
GAZA
(S) BOBIN
II-8156-RESELECTION
FEDERATION
FORTYFOLD
ORO
FLORENCE
TURKEY-RED
ORO
BREVOR
FEDERATION
FORTYFOLD
BREVON
FLORENCE
TURKEY
TOHOKU-34
TURKEY-RED
(S) CRIMEAN
FULTZ
DARUMA
PENJAMO-T-62
KANRED
MARQUIS
2 * THATCHER
IUMILLO
MARQUIS
NEWTHATCH
THATCHER
HOPE
KENYA-58
KENYA-BF-4-3-B-10-V-1
RED-EGYPTIAN
FRONTANA
MENTANA
FRONTEIRA
MENGAVI-8156-R
(S) BOBIN
STEINWEDEL
THEW
6 * GABO
GAZA
LV-PAL
(S) BOBIN
AKAKOMUGI
LV-FUKUI
MENTANA
WILHELMINA
SQUAREHEAD
SPIJK
RIETI
MENGAVI
(S) BOBIN
STEINWEDEL
THEW
2 * GABO
GAZA
LV-PAL
(S) BOBIN
CI-12632
DUNDEE
SANDS
CLEVELAND
HARD-FEDERATION
EUREKA,AUS
FLORENCE
KENYA-C-6040

ZENKOJIKOMUGI —— (M) IGACHIKUGO-OREGON
- OREGON —— (S) SURPRISE
  - MICHIGAN-CLUB
  - CHILE-CLUB
- IGACHIKUGO —— LV-SAGA

ZHONGYOU-9507 ——(S) ZHONGZUO-8131-1
